# Supplementary material for: Diversity patterns in terrestrial tetrapod clades are governed by equilibrium dynamics
Source: PLoS Biol. 2026 Apr 13;24(4):e3003730. doi: 10.1371/journal.pbio.3003730 (PMC13075687; doi:10.1371/journal.pbio.3003730)
Supplement: S1 Appendix — A Fig. Species richness patterns for (a) amphibians (n = 5,235 species), (b) reptiles (n = 9,129 species), (c) birds (n = 9,324 species), and (d) mammals (n = 5,145 species). Species richness was estimated by aggregating species distribution range maps into equal-area grid cells at a spatial resolution of ~1° × 1°, and summing the number of species whose geographic ranges overlapped each grid cell. The maps shown here illustrate overall richness patterns for each tetrapod class, however, all subsequent analyses were conducted on species richness patterns calculated separately for each focal clade. Continental boundaries were obtained from Natural Earth Admin 0 Country Boundaries (https://www.naturalearthdata.com), which is in the public domain (CC0) and compatible with the CC BY 4.0 license. The map was generated in R using this base layer. The data underlying this figure can be found in https://doi.org/10.5281/zenodo.14008084. B Fig. Global spatial distribution of environmental predictors used in the analyses: (a) mean annual temperature, (b) mean annual precipitation, and (c) net primary productivity (NPP). Continental boundaries were obtained from Natural Earth Admin 0 Country Boundaries (https://www.naturalearthdata.com), which is in the public domain (CC0) and compatible with the CC BY 4.0 license. The map was generated in R using this base layer. The data underlying this figure can be found in https://doi.org/10.5281/zenodo.14008084. C Fig. Conceptual illustration of the workflow used to estimate evolutionary time (assemblage age) across tetrapod clades. The example shows a hypothetical clade and summarizes the main analytical steps. (a) Spatial variation in species richness across grid cells within the clade. (b) Phylogenetic turnover among assemblages is quantified using species distributions and phylogenetic relationships to delineate evolutionary regions defined by shared evolutionary history. (c) Ancestral geographic ranges are reconstructed along the phy [file pbio.3003730.s001.doc]

**S1 Appendix: Supporting Information**

**Diversity patterns in terrestrial tetrapod clades are governed by equilibrium dynamics**

Felipe O. Cerezer^1,2,*^, Antonin Machac^2,4^, Jan Smyčka^1,3^, Iñigo Rubio-López^2,4^, Maxime Quétin^2,4^ & David Storch^1,4,*^

^1^Center for Theoretical Study, Charles University, Prague, Czech Republic

^2^Laboratory of Environmental Microbiology, Institute of Microbiology of the Czech Academy of Sciences, Prague, Czech Republic

^3^Department of Biological Sciences, Simon Fraser University, Burnaby, Canada

^4^Department of Ecology, Faculty of Science, Charles University, Prague, Czech Republic

This appendix contains all supporting figures and tables referenced in the main text. Figures are labeled Fig A–Fig AW and tables as Table A–Table B.


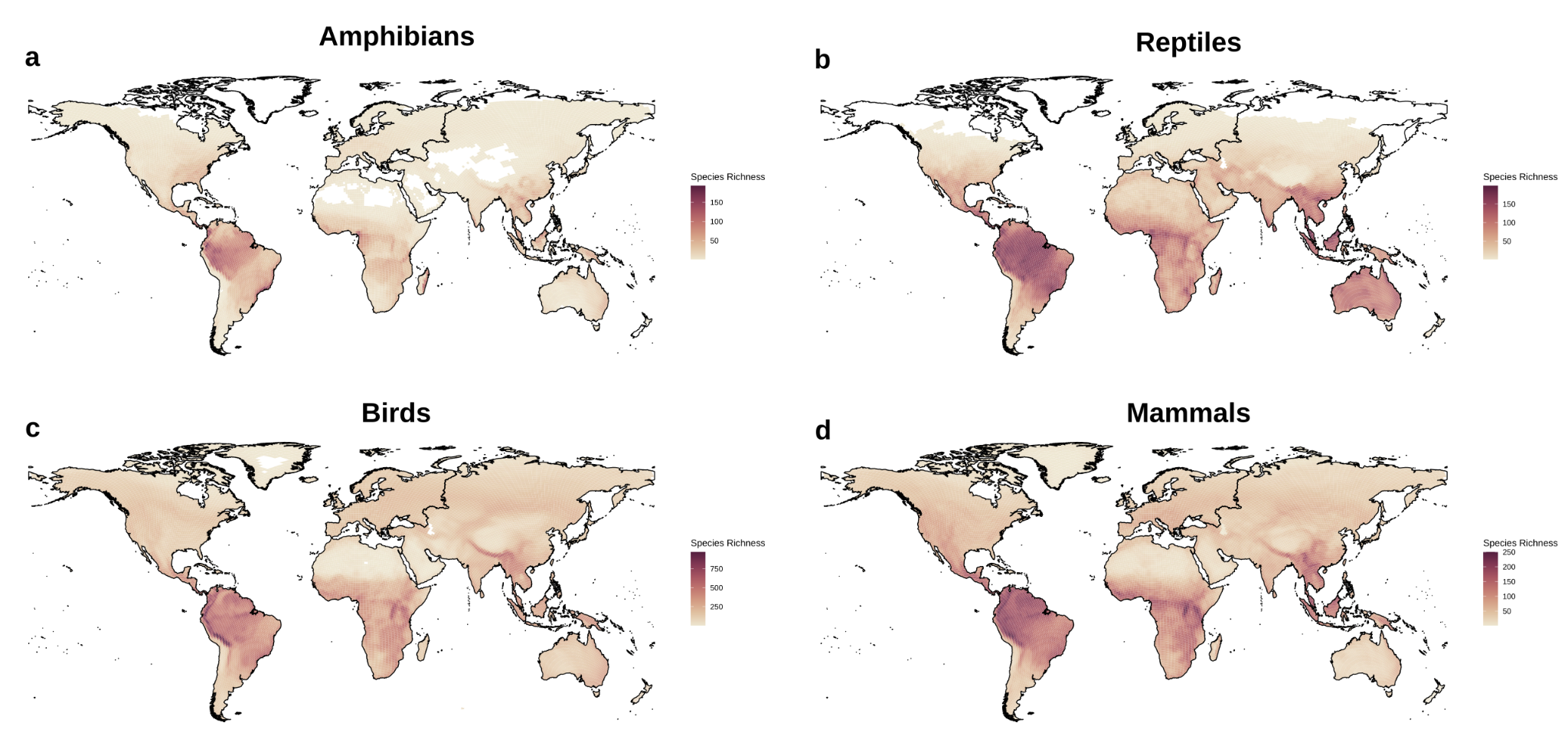


**Fig A. Species richness patterns for (a) amphibians (n = 5,235 species), (b) reptiles (n = 9,129 species), (c) birds (n = 9,324 species), and (d) mammals (n = 5,145 species).** Species richness was estimated by aggregating species distribution range maps into equal-area grid cells at a spatial resolution of approximately 1° × 1°, and summing the number of species whose geographic ranges overlapped each grid cell. The maps shown here illustrate overall richness patterns for each tetrapod class, however, all subsequent analyses were conducted on species richness patterns calculated separately for each focal clade. Continental boundaries were obtained from Natural Earth Admin 0 Country Boundaries ([https://www.naturalearthdata.com](https://www.naturalearthdata.com/)), which is in the public domain (CC0) and compatible with the CC BY 4.0 license. The map was generated in R using this base layer. The data underlying this figure can be found in <https://doi.org/10.5281/zenodo.14008084>


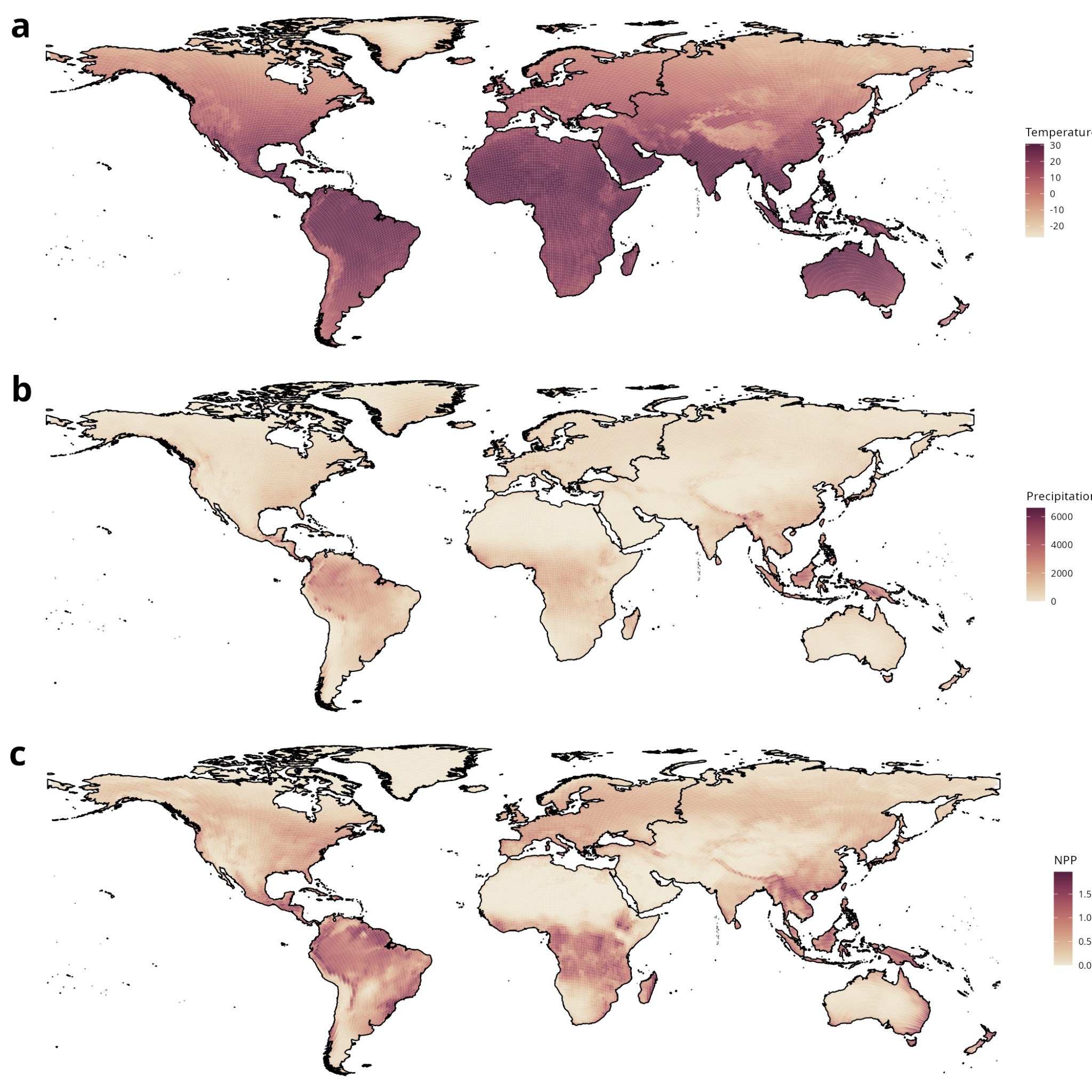


**Fig B. Global spatial distribution of environmental predictors used in the analyses:** (a) mean annual temperature, (b) mean annual precipitation, and (c) net primary productivity (NPP). Continental boundaries were obtained from Natural Earth Admin 0 Country Boundaries ([https://www.naturalearthdata.com](https://www.naturalearthdata.com/)), which is in the public domain (CC0) and compatible with the CC BY 4.0 license. The map was generated in R using this base layer. The data underlying this figure can be found in <https://doi.org/10.5281/zenodo.14008084>


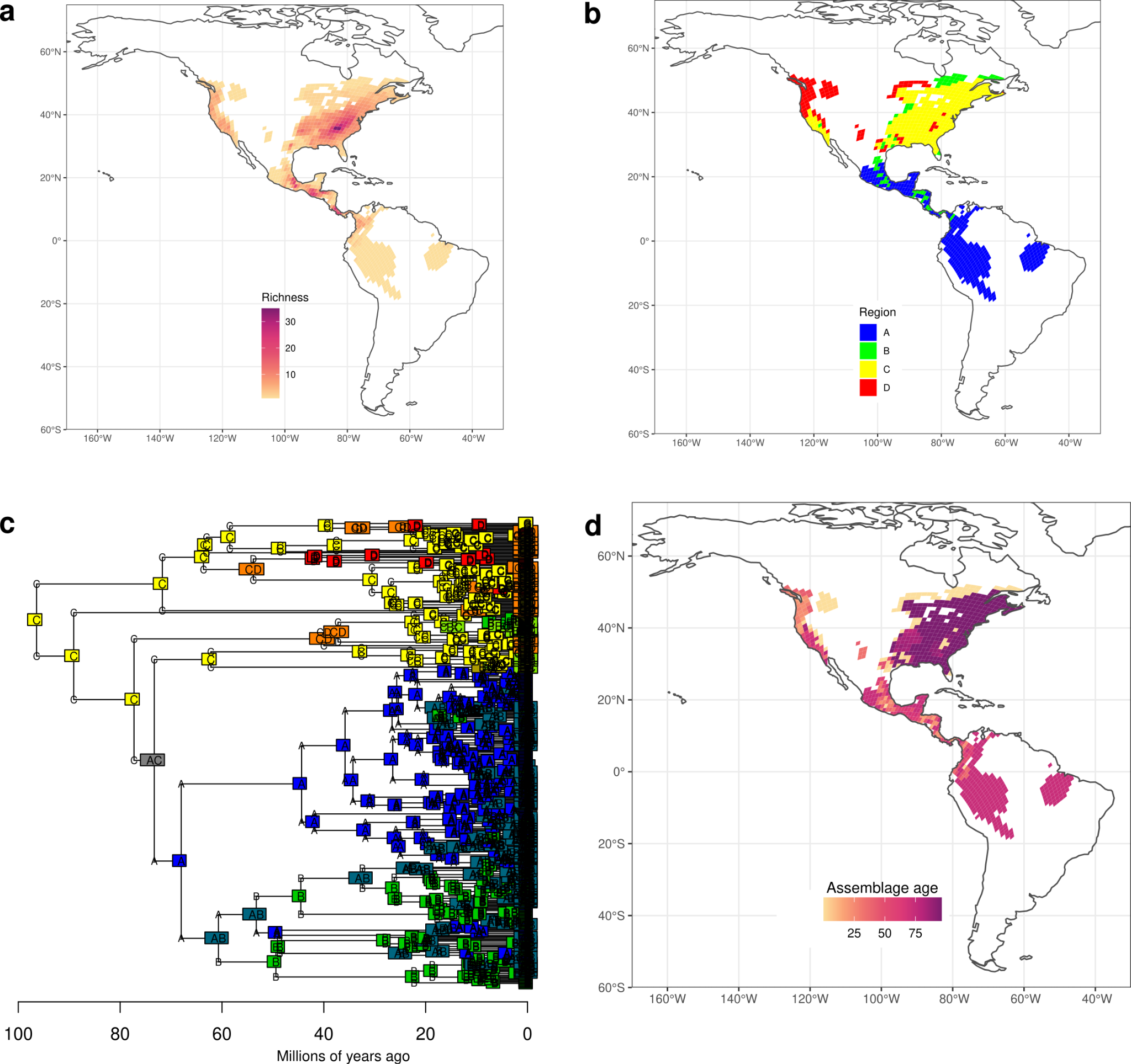


**Fig C. Conceptual illustration of the workflow used to estimate evolutionary time (assemblage age) across tetrapod clades.** The example shows a hypothetical clade and summarizes the main analytical steps. (a) Spatial variation in species richness across grid cells within the clade. (b) Phylogenetic turnover among assemblages is quantified using species distributions and phylogenetic relationships to delineate evolutionary regions defined by shared evolutionary history. (c) Ancestral geographic ranges are reconstructed along the phylogeny using the DEC model implemented in *BioGeoBEARS*, yielding probabilistic estimates of ancestral area for each node. (d) For each species present in a given grid cell, the phylogeny is traced back to the deepest ancestral node whose reconstructed range includes the focal evolutionary region. The age of this node is interpreted as the species’ arrival time into the assemblage (grid cell). Assemblage age is then calculated as the mean arrival time across all species occurring within a grid cell. Continental boundaries were obtained from Natural Earth Admin 0 Country Boundaries ([https://www.naturalearthdata.com](https://www.naturalearthdata.com/)), which is in the public domain (CC0) and compatible with the CC BY 4.0 license. The map was generated in R using this base layer. The data underlying this figure can be found in <https://doi.org/10.5281/zenodo.14008084>


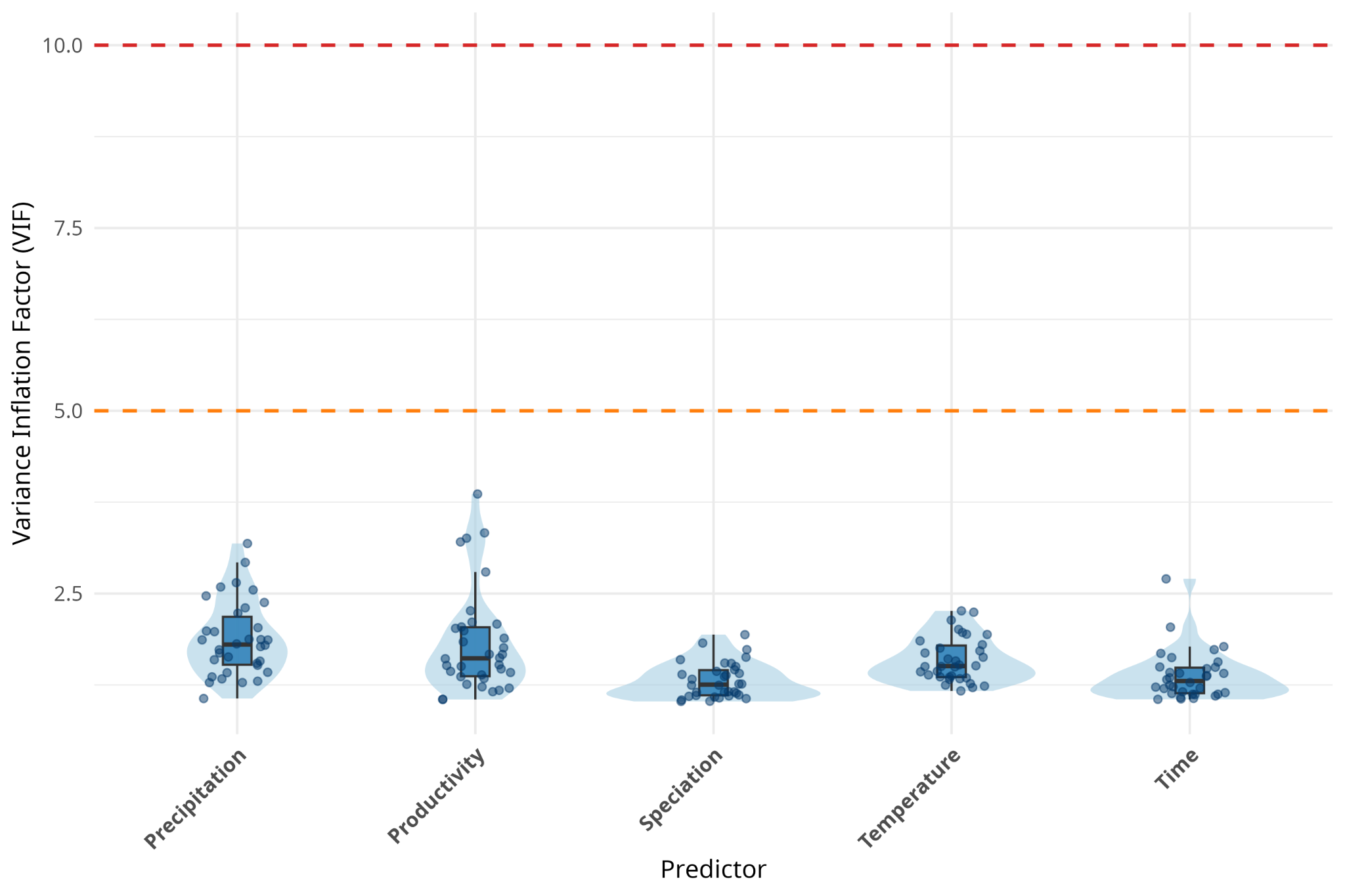


**Fig D. Variance inflation factors (VIF) for predictors of species richness across amphibian clades.** Violins show the distribution of VIF values across clades for each predictor, with embedded boxplots indicating medians and interquartile ranges. Points represent individual clades. Predictors include evolutionary time (assemblage age), speciation rate (DR), temperature, precipitation, and productivity (NPP). Dashed horizontal lines indicate VIF thresholds of 5 (moderate multicollinearity) and 10 (high multicollinearity). The data underlying this figure can be found in <https://doi.org/10.5281/zenodo.14008084>


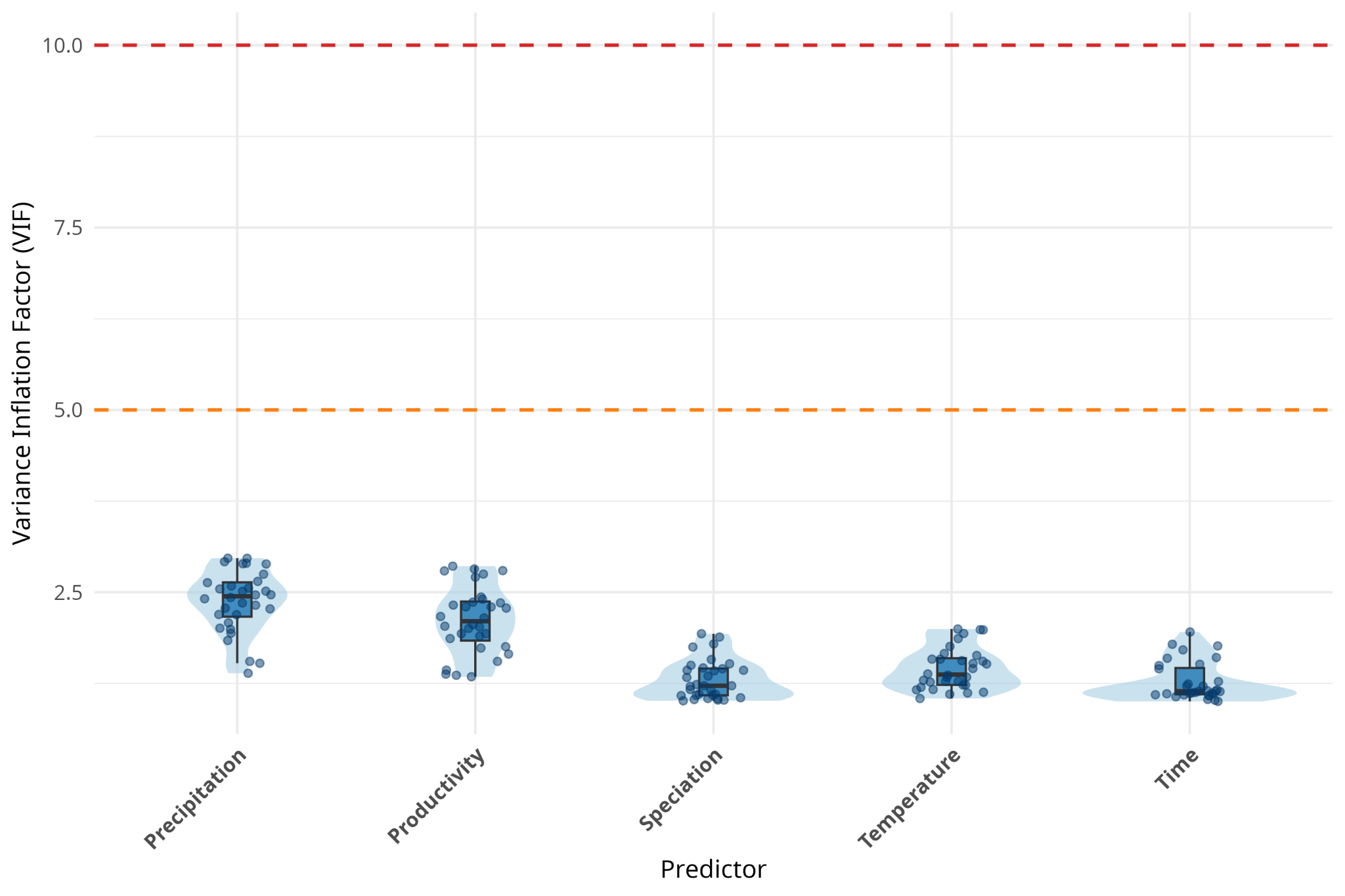


**Fig E. Variance inflation factors (VIF) for predictors of species richness across reptile clades.** Violins show the distribution of VIF values across clades for each predictor, with embedded boxplots indicating medians and interquartile ranges. Points represent individual clades. Predictors include evolutionary time (assemblage age), speciation rate (DR), temperature, precipitation, and productivity (NPP). Dashed horizontal lines indicate VIF thresholds of 5 (moderate multicollinearity) and 10 (high multicollinearity). The data underlying this figure can be found in <https://doi.org/10.5281/zenodo.14008084>


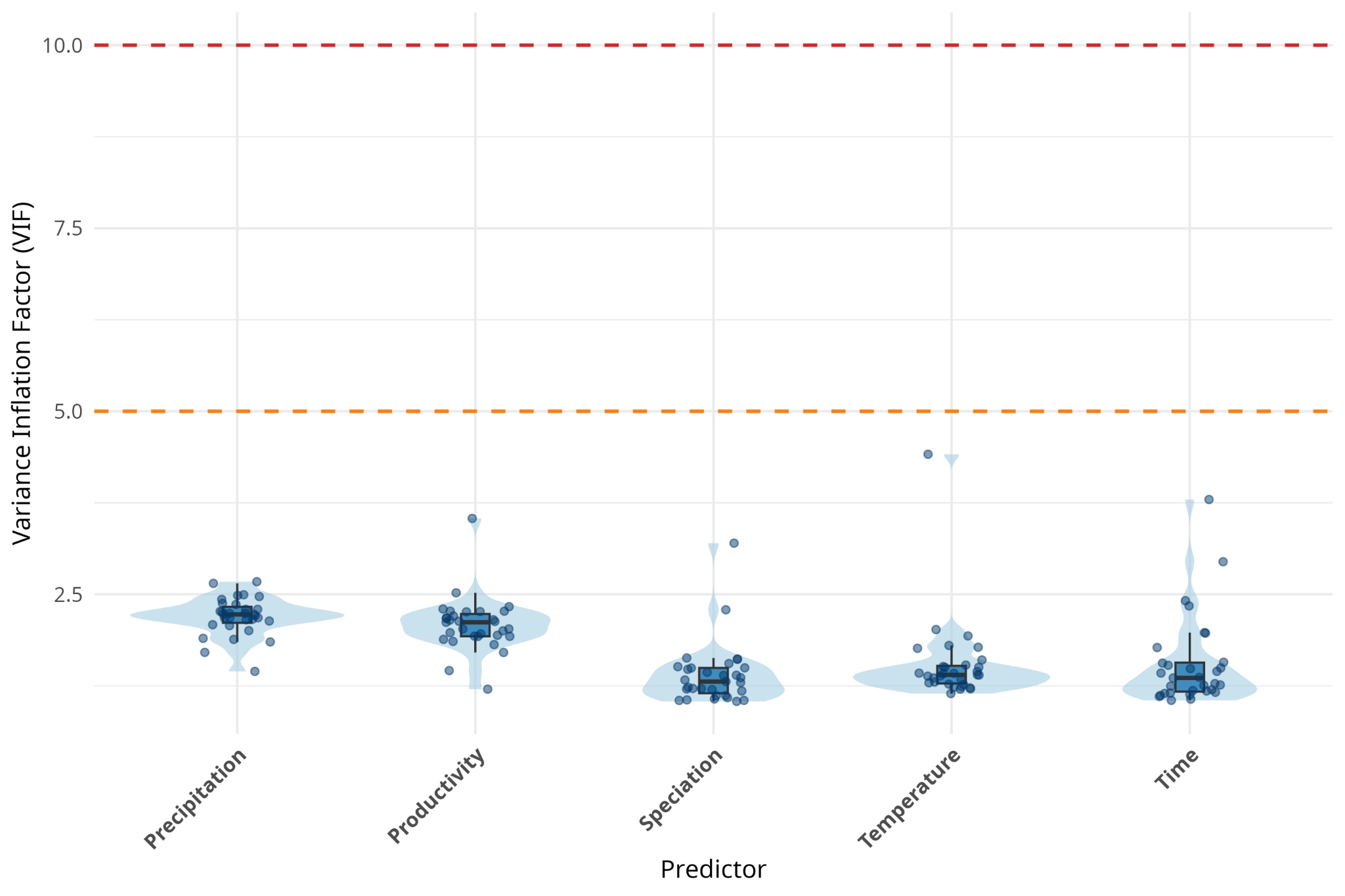


**Fig F. Variance inflation factors (VIF) for predictors of species richness across bird clades.** Violins show the distribution of VIF values across clades for each predictor, with embedded boxplots indicating medians and interquartile ranges. Points represent individual clades. Predictors include evolutionary time (assemblage age), speciation rate (DR), temperature, precipitation, and productivity (NPP). Dashed horizontal lines indicate VIF thresholds of 5 (moderate multicollinearity) and 10 (high multicollinearity). The data underlying this figure can be found in <https://doi.org/10.5281/zenodo.14008084>


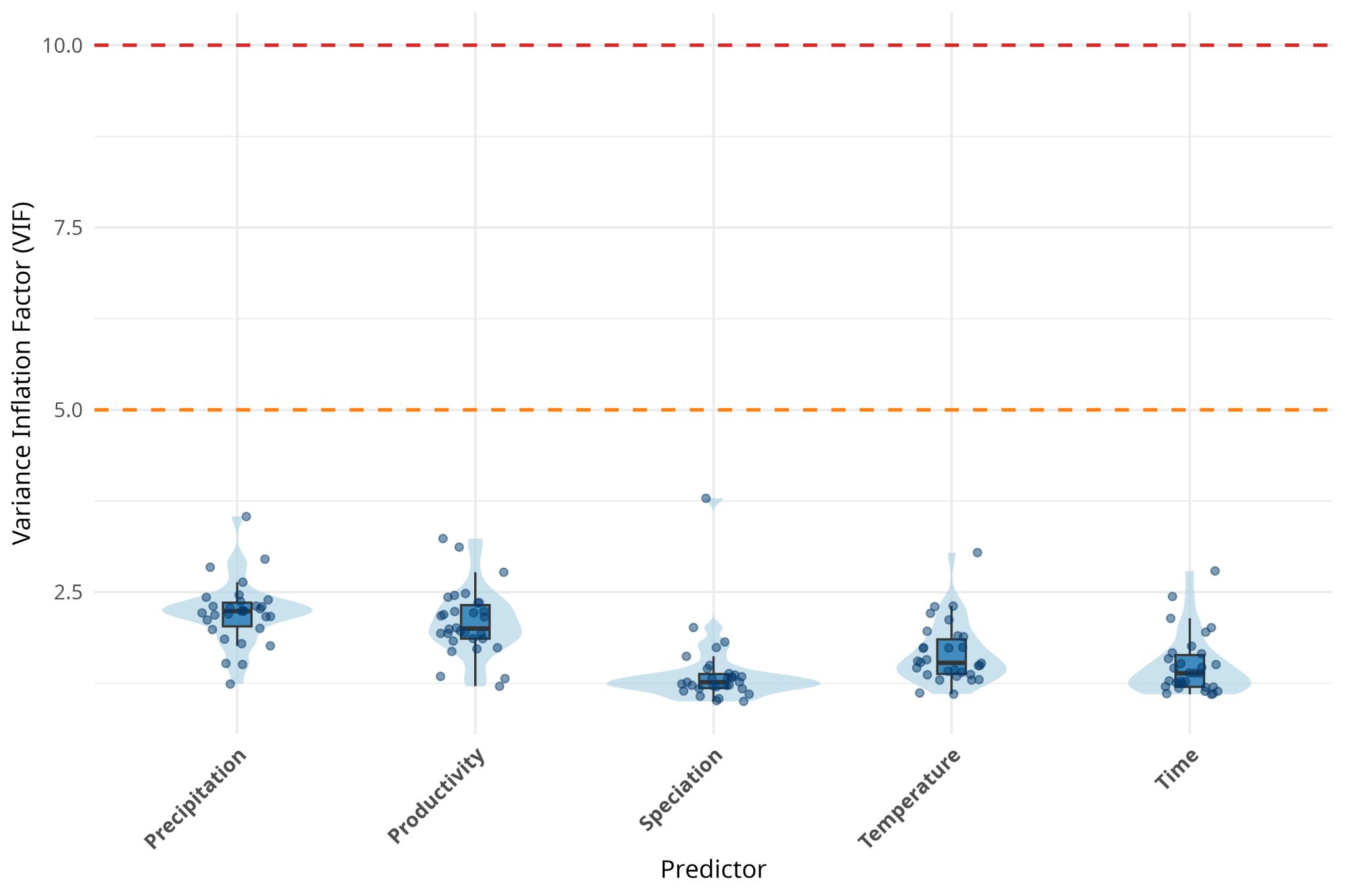


**Fig G. Variance inflation factors (VIF) for predictors of species richness across mammal clades.** Violins show the distribution of VIF values across clades for each predictor, with embedded boxplots indicating medians and interquartile ranges. Points represent individual clades. Predictors include evolutionary time (assemblage age), speciation rate (DR), temperature, precipitation, and productivity (NPP). Dashed horizontal lines indicate VIF thresholds of 5 (moderate multicollinearity) and 10 (high multicollinearity). The data underlying this figure can be found in <https://doi.org/10.5281/zenodo.14008084>


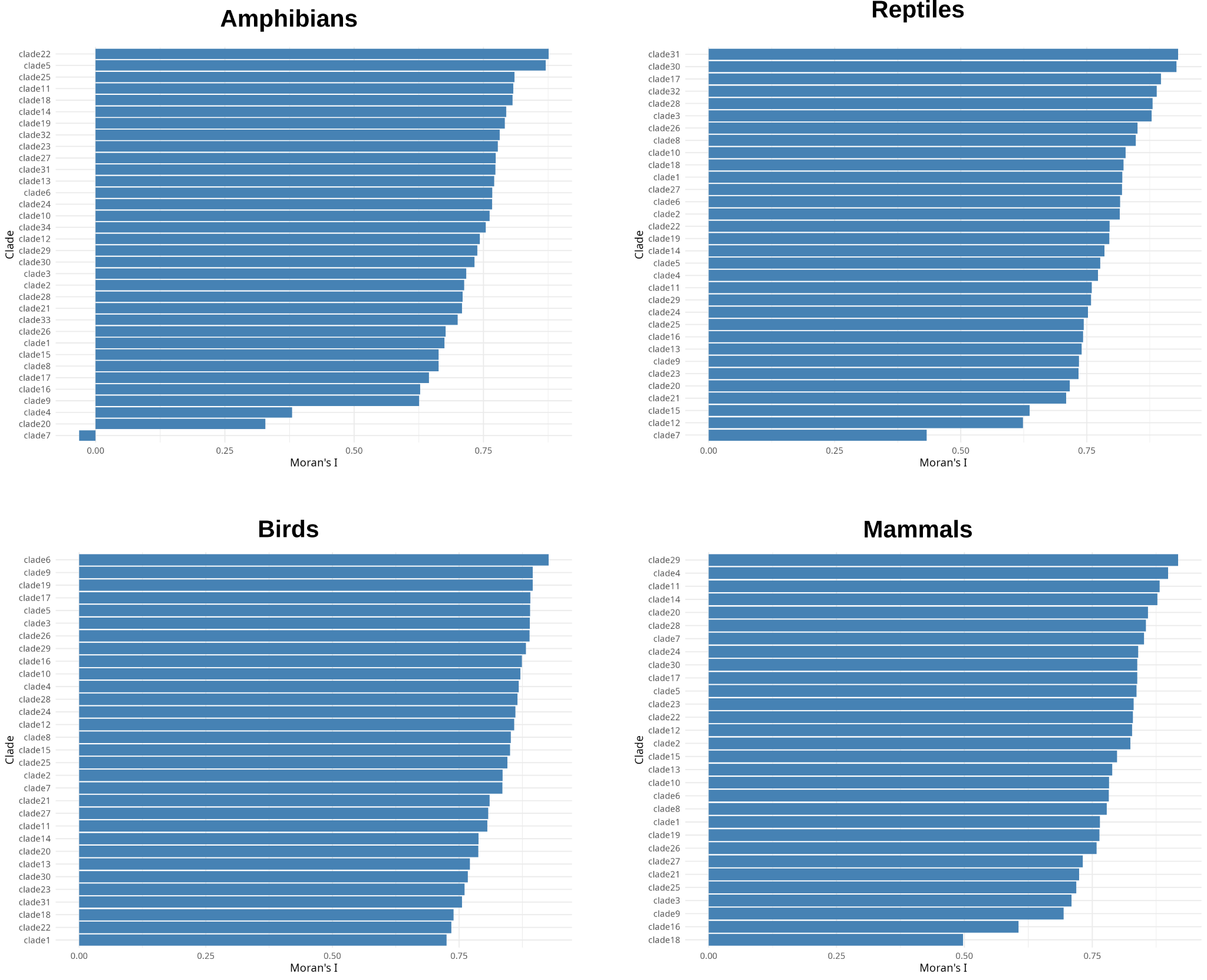


**Fig H. Spatial autocorrelation of residuals from clade-specific path models across tetrapod classes.** Bars show Moran’s I values calculated for model residuals within each clade for amphibians, reptiles, birds, and mammals. The data underlying this figure can be found in <https://doi.org/10.5281/zenodo.14008084>


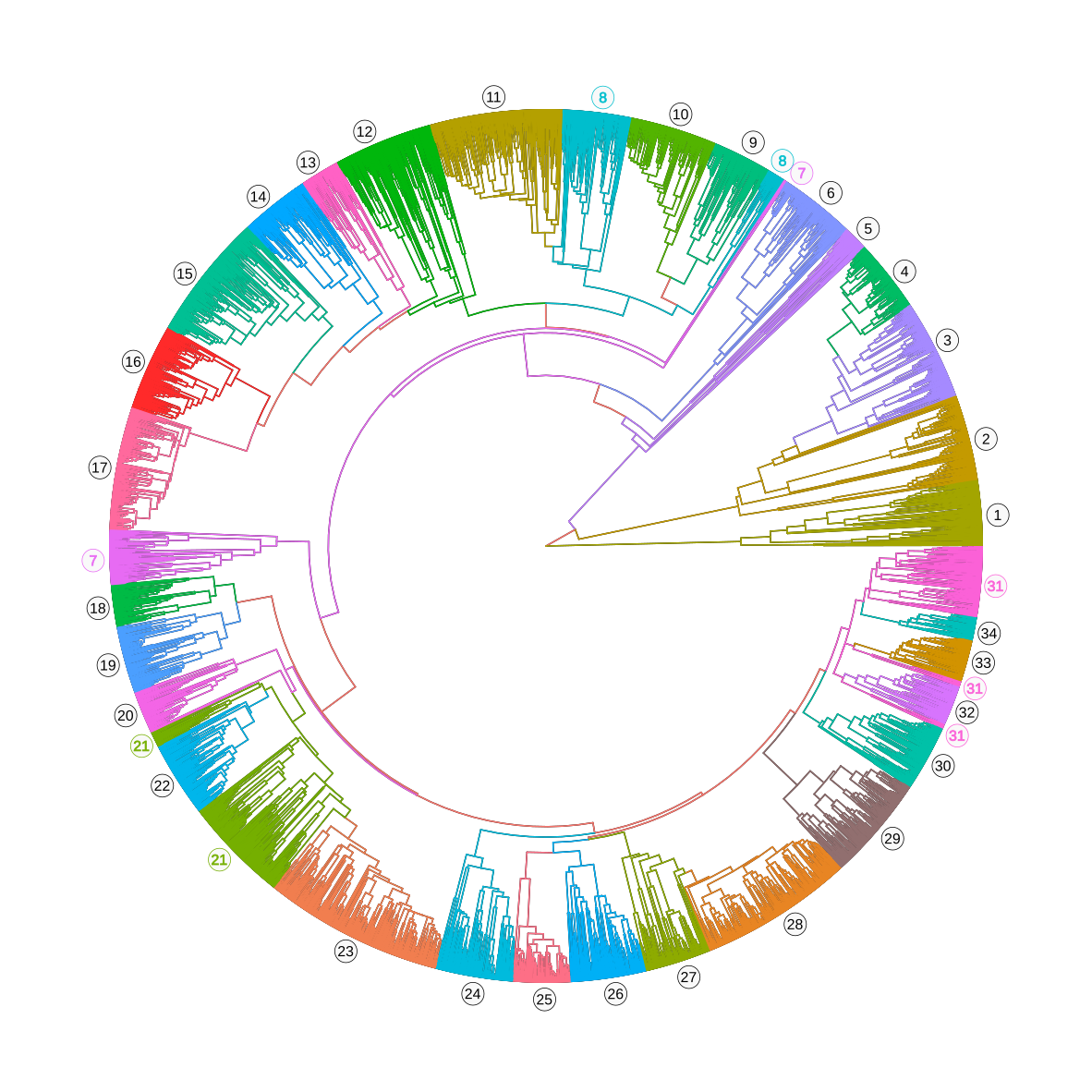


**Fig I. Amphibian phylogeny showing clades delineated using the Laplacian spectrum approach.** This procedure identified 34 amphibian clades with more than 50 species each, totaling 6,361 species. Clades with fewer than 50 species were excluded from downstream analyses. Colours and associated numbers denote distinct clades. Numbers highlighted in colour (e.g., clade 31) indicate paraphyletic clades. Paraphyletic clades represent a minority of cases, and their exclusion did not alter the main results. The data underlying this figure can be found in <https://doi.org/10.5281/zenodo.14008084>


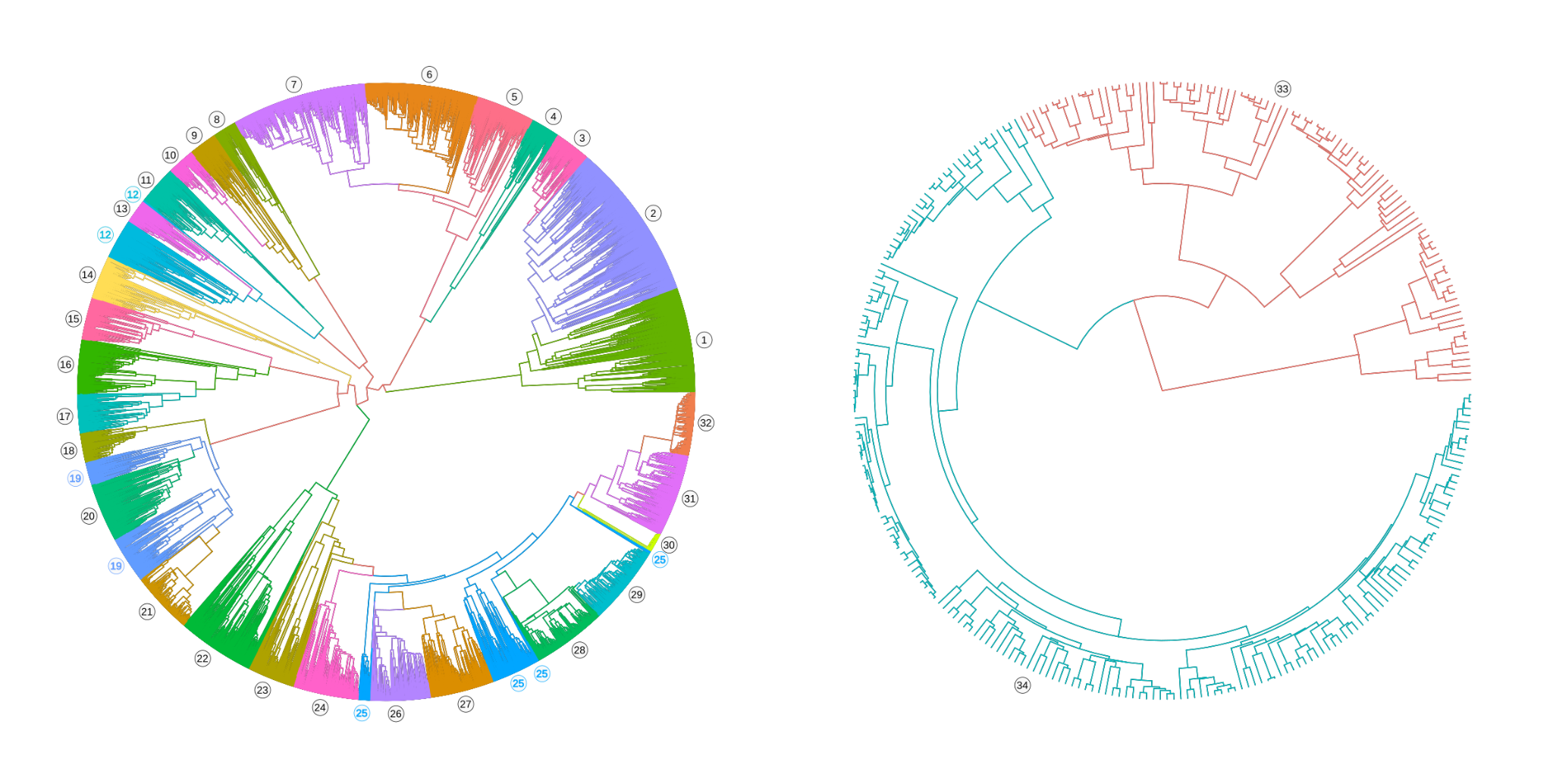


**Fig J. Reptile phylogeny showing clades delineated using the Laplacian spectrum approach.** The analysis identified 34 reptile clades with more than 50 species each, comprising 32 squamate clades (left panel) and 2 turtle–crocodilian clades (right panel), totaling 9,081 species. Clades with fewer than 50 species were excluded from downstream analyses. Colours and associated numbers denote distinct clades. Numbers highlighted in colour (e.g., clade 25) indicate paraphyletic clades. Paraphyletic clades were rare, and their exclusion did not affect the main conclusions. The data underlying this figure can be found in <https://doi.org/10.5281/zenodo.14008084>


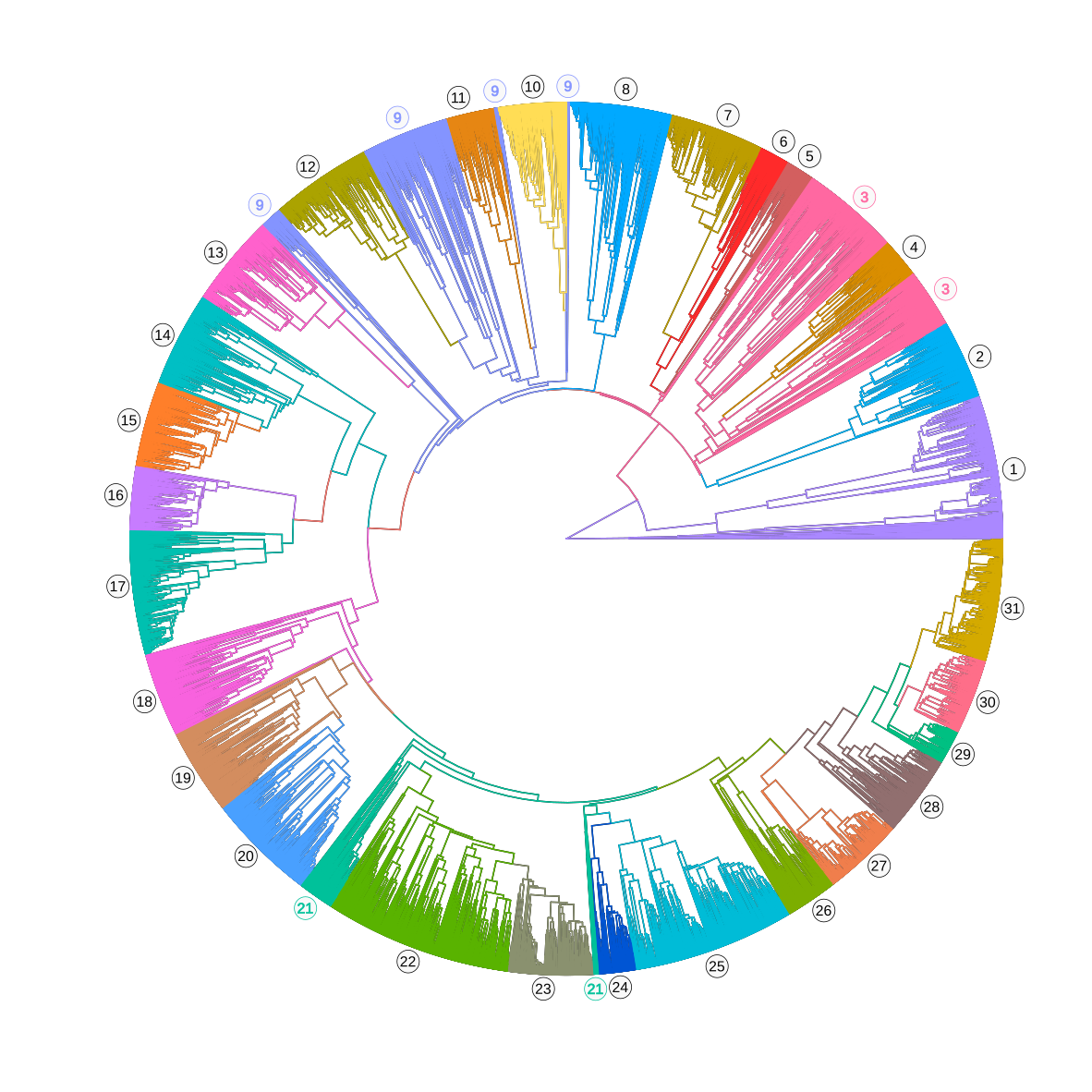


**Fig K. Bird phylogeny showing clades delineated using the Laplacian spectrum approach.** This method identified 31 bird clades with more than 50 species each, totaling 9,232 species. Clades with fewer than 50 species were excluded from downstream analyses. Colours and associated numbers denote distinct clades. Numbers highlighted in colour (e.g., clade 3) indicate paraphyletic clades. These clades represent a small fraction of the dataset and do not influence the overall results. The data underlying this figure can be found in <https://doi.org/10.5281/zenodo.14008084>


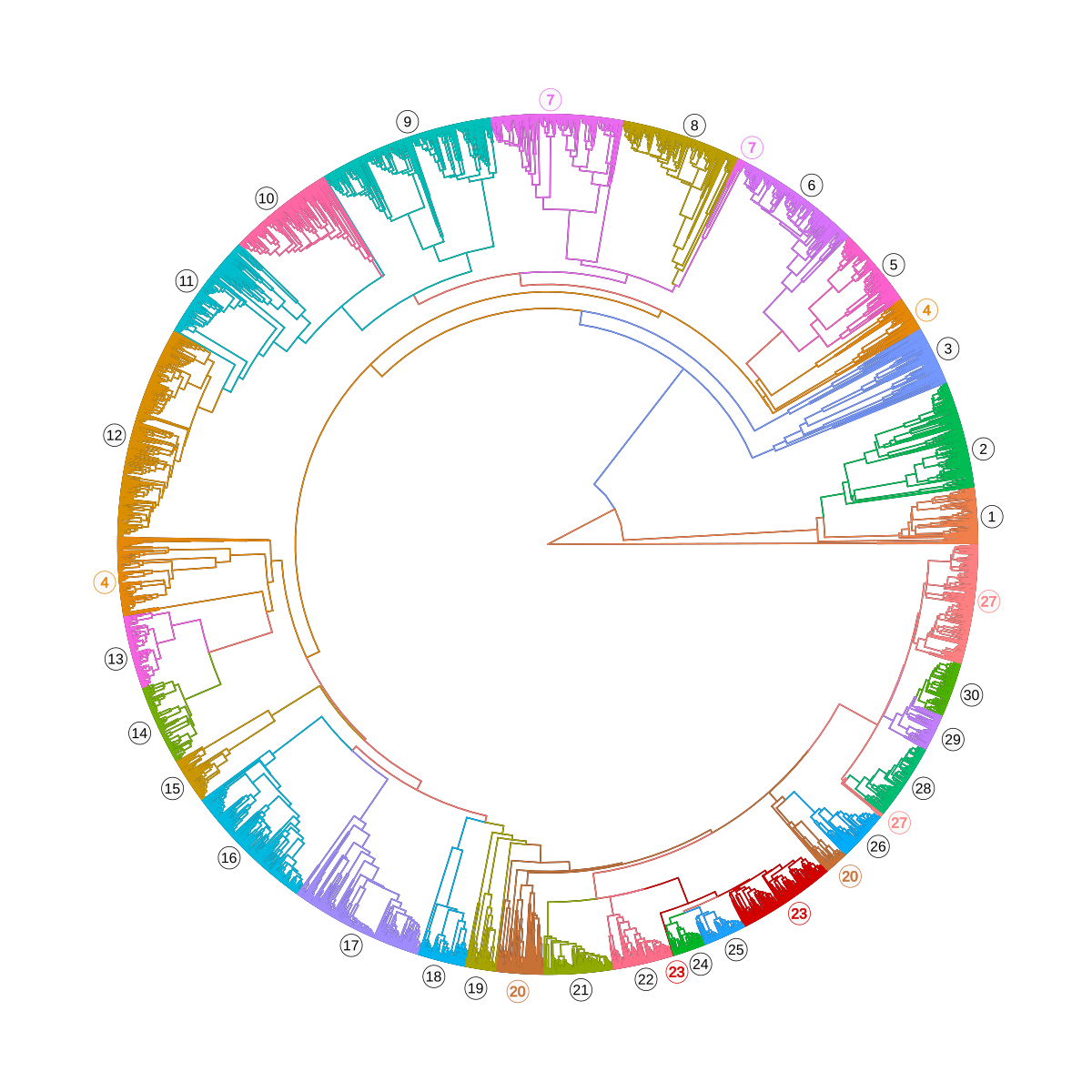


**Fig L. Mammal phylogeny showing clades delineated using the Laplacian spectrum approach.** The analysis identified 30 mammal clades with more than 50 species each, totaling 5,032 species. Clades with fewer than 50 species were excluded from downstream analyses. Colours and associated numbers denote distinct clades. Numbers highlighted in colour (e.g., clade 7) indicate paraphyletic clades. The exclusion of these clades did not change the main findings. The data underlying this figure can be found in <https://doi.org/10.5281/zenodo.14008084>


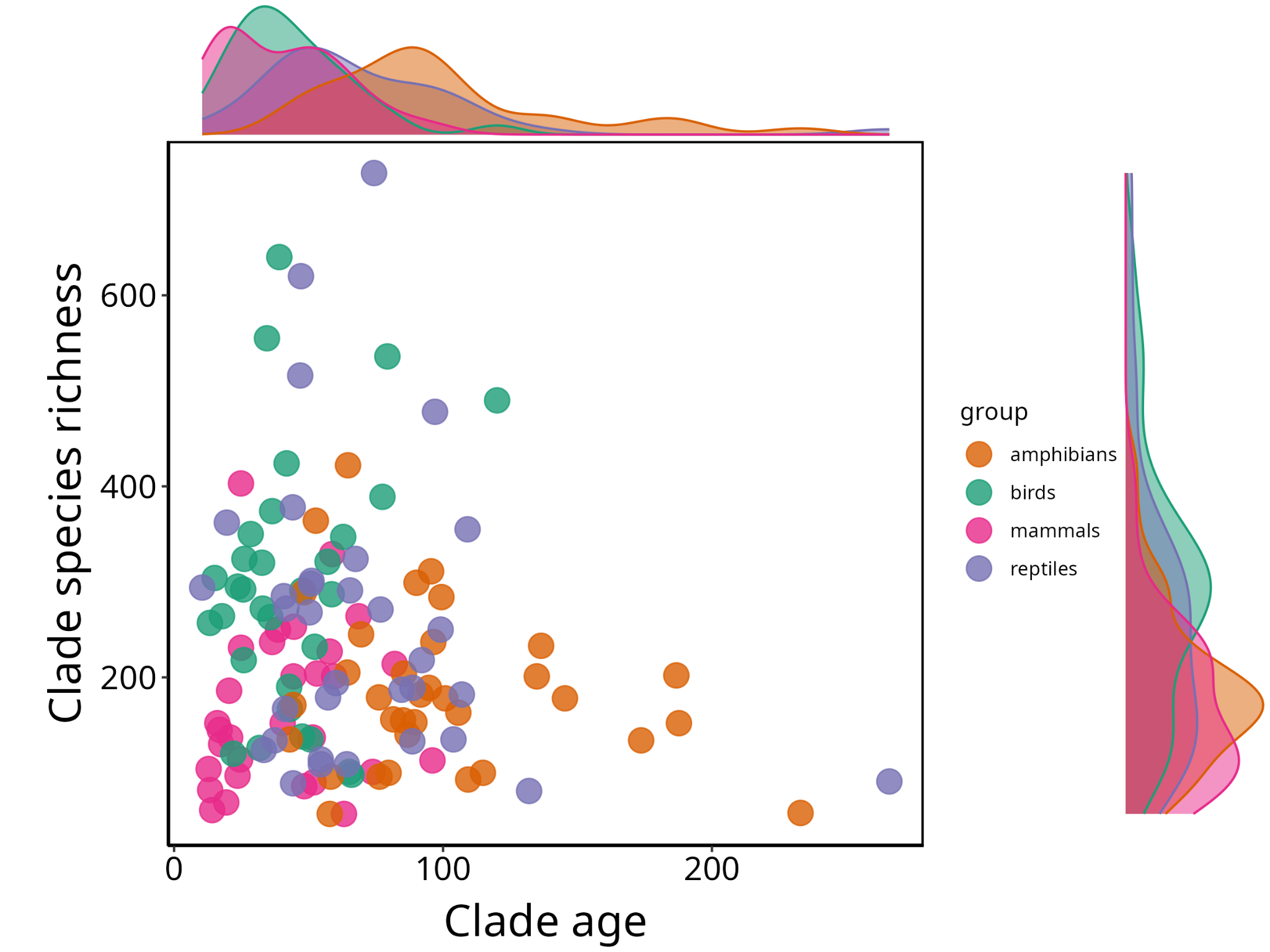


**Fig M. Variation in species richness and crown age across tetrapod clades.** Each circle represents an individual clade, with colours indicating tetrapod class (34 amphibian clades, 34 reptile clades, 31 bird clades, and 30 mammal clades). Marginal density plots show the distributions of species richness and clade age within each tetrapod class. The data underlying this figure can be found in <https://doi.org/10.5281/zenodo.14008084>


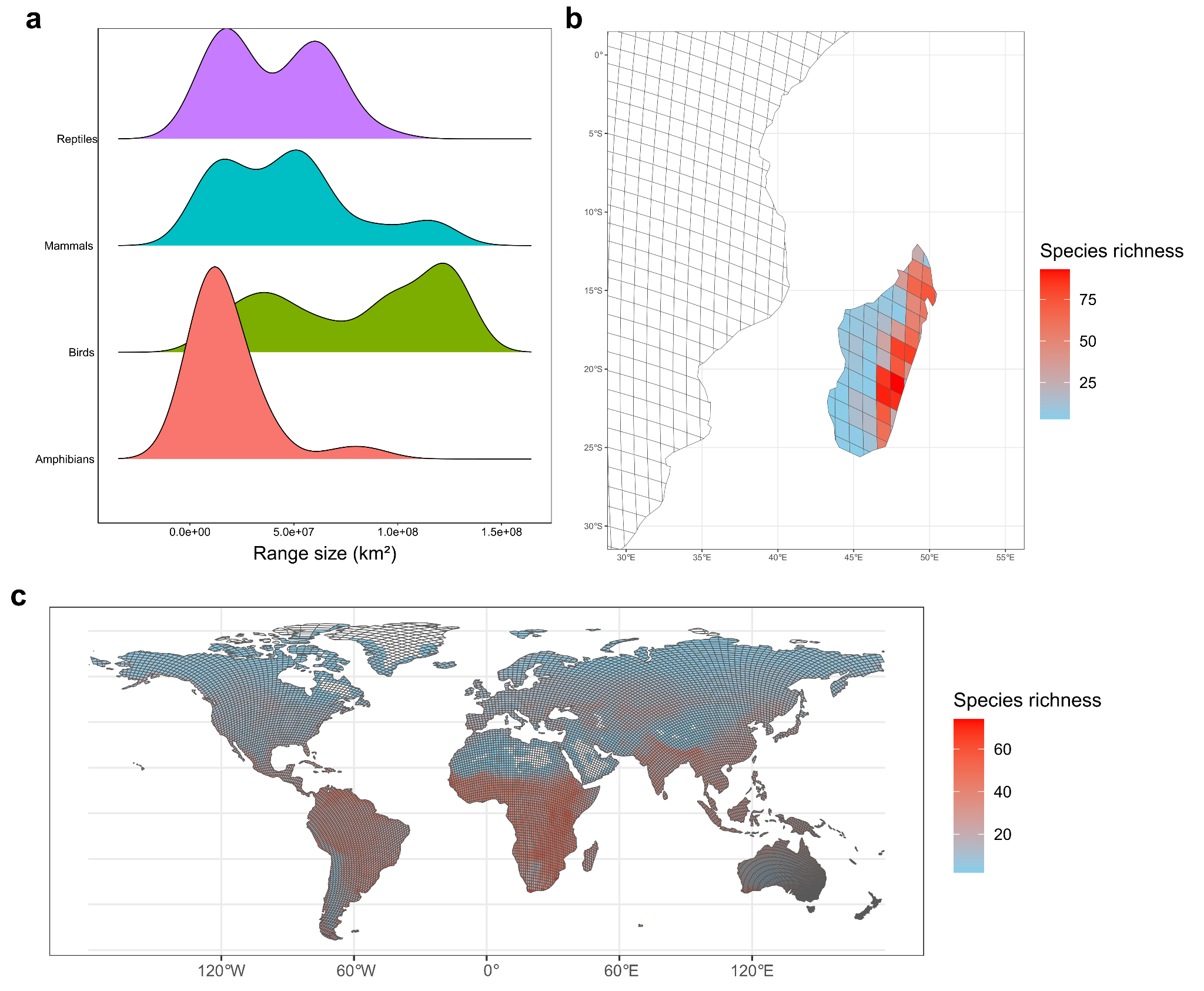


**Fig N. Geographic extent of terrestrial tetrapod clades included in the analyses.** (a) Density distributions of clade geographic extent (range size) for amphibians, reptiles, birds, and mammals. (b) Spatial distribution of the smallest clade geographic extent observed, corresponding to an amphibian clade. (c) Spatial distribution of the largest clade geographic extent observed, corresponding to a bird clade. Continental boundaries were obtained from Natural Earth Admin 0 Country Boundaries ([https://www.naturalearthdata.com](https://www.naturalearthdata.com/)), which is in the public domain (CC0) and compatible with the CC BY 4.0 license. The map was generated in R using this base layer. The data underlying this figure can be found in <https://doi.org/10.5281/zenodo.14008084>


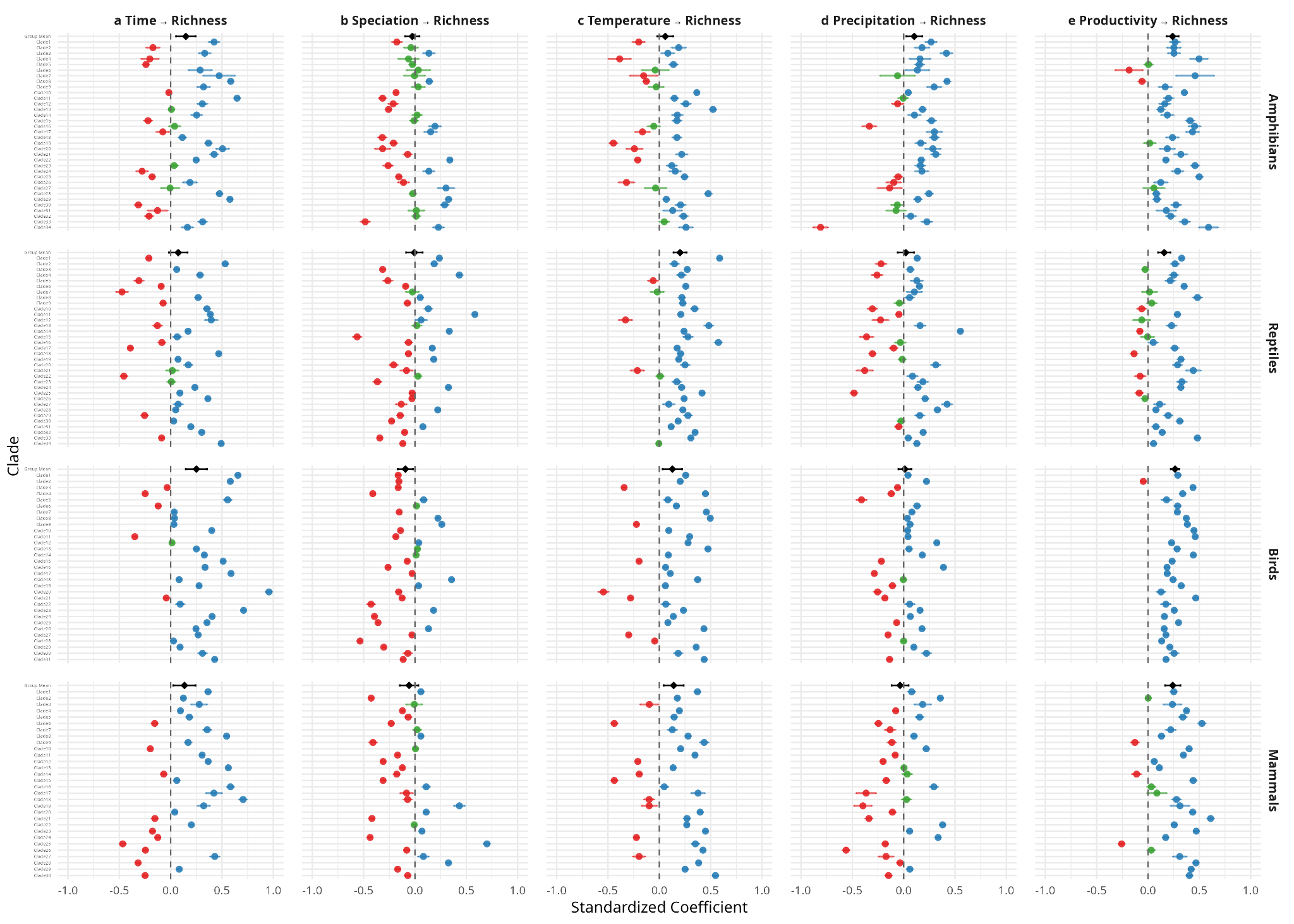


**Fig O. Direct effects of environmental factors (temperature, precipitation, and productivity), evolutionary time (assemblage age), and speciation rate on species richness across tetrapod clades**. Points represent mean standardized path coefficients (β) across clades, with error bars indicating 95% confidence intervals, shown separately for amphibians, reptiles, birds, and mammals. Black diamonds indicate the mean effect size of each predictor across clades within each tetrapod class. Colours denote effect direction and statistical significance: blue, positive and significant; red, negative and significant; green, non-significant (confidence intervals overlapping zero). The data underlying this figure can be found in <https://doi.org/10.5281/zenodo.14008084>


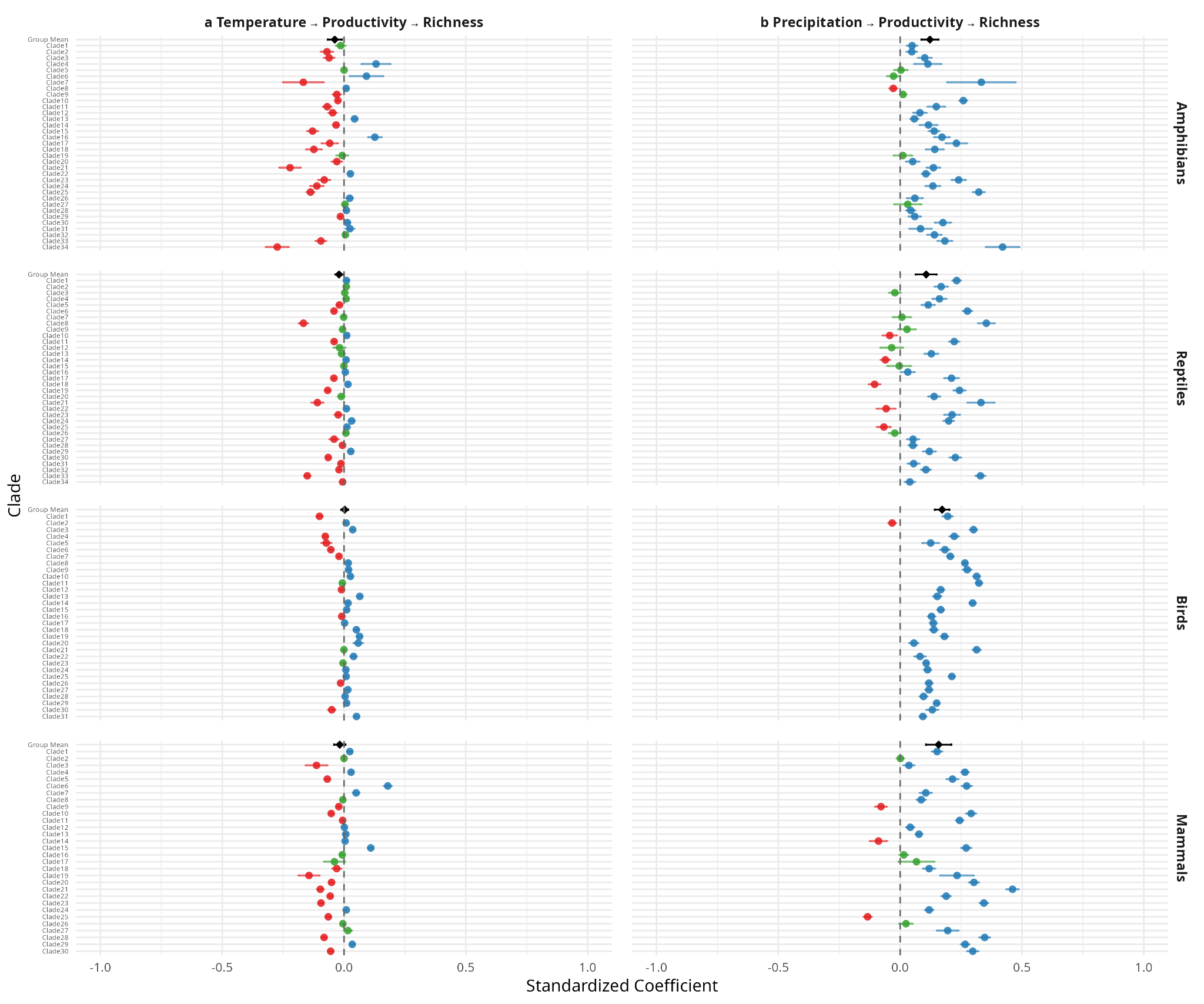


**Fig P. Indirect effects of climate mediated through productivity on species richness across tetrapod clades.** Points represent mean standardized path coefficients (β) across clades, with error bars indicating 95% confidence intervals, shown separately for amphibians, reptiles, birds, and mammals. Black diamonds indicate the mean effect size of each predictor across clades within each tetrapod class. Colours denote effect direction and statistical significance: blue, positive and significant; red, negative and significant; green, non-significant (confidence intervals overlapping zero). The data underlying this figure can be found in <https://doi.org/10.5281/zenodo.14008084>


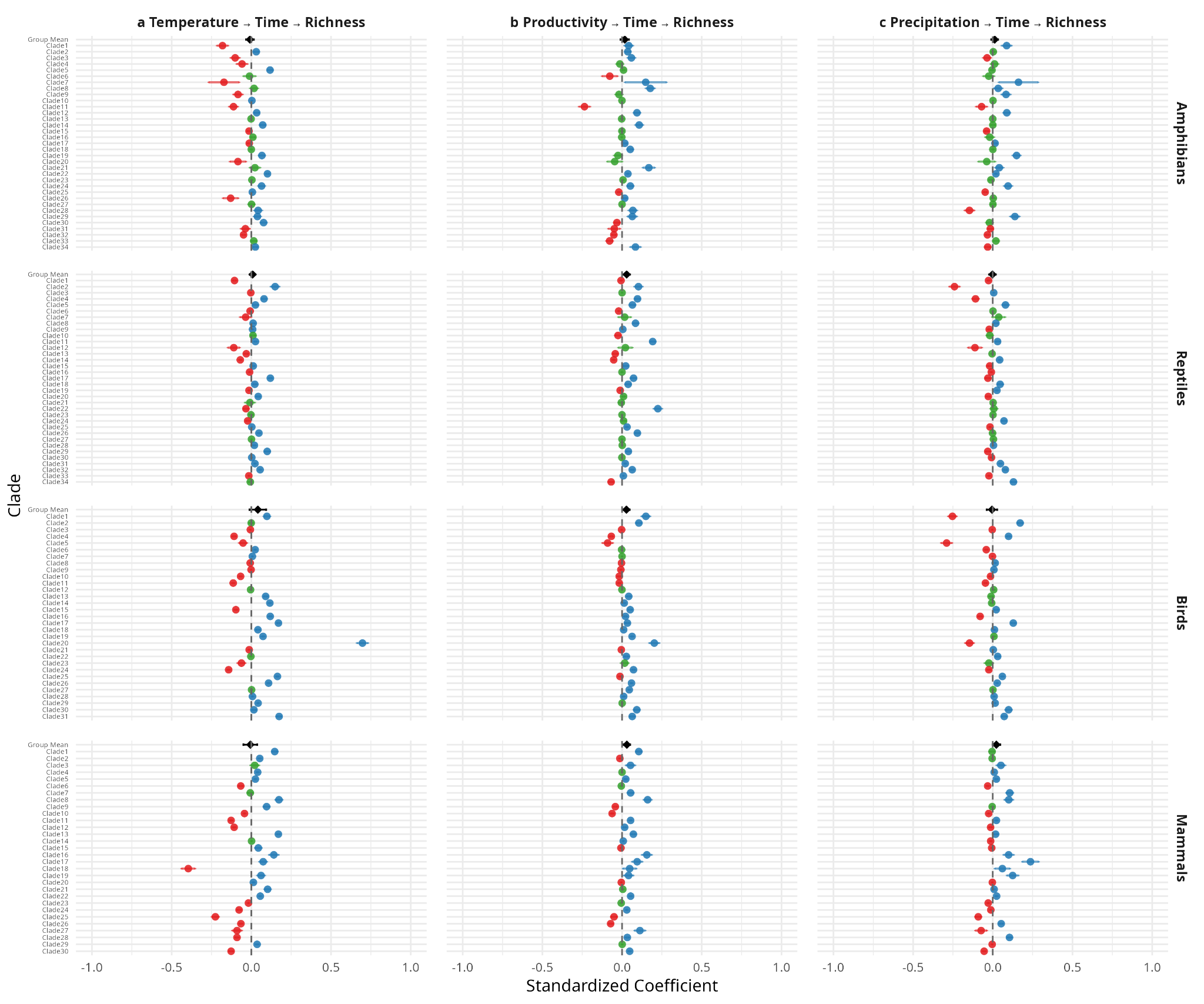


**Fig Q. Indirect effects of environmental factors mediated through evolutionary time (assemblage age) on species richness across tetrapod clades.** Points represent mean standardized path coefficients (β) across clades, with error bars indicating 95% confidence intervals, shown separately for amphibians, reptiles, birds, and mammals. Black diamonds indicate the mean effect size of each predictor across clades within each tetrapod class. Colours denote effect direction and statistical significance: blue, positive and significant; red, negative and significant; green, non-significant (confidence intervals overlapping zero). The data underlying this figure can be found in <https://doi.org/10.5281/zenodo.14008084>


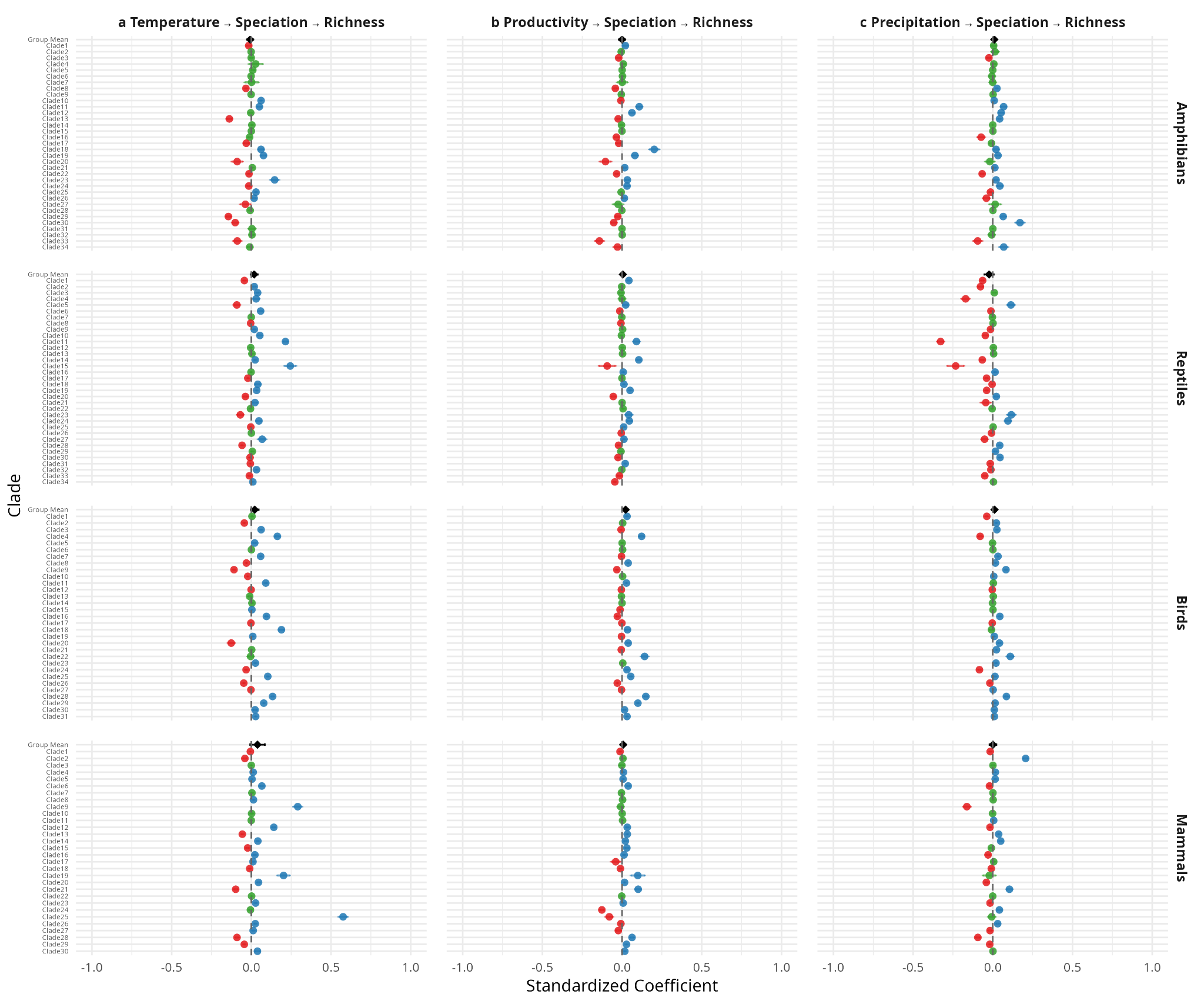


**Fig R. Indirect effects of environmental factors mediated through speciation rate on species richness across tetrapod clades.** Points represent mean standardized path coefficients (β) across clades, with error bars indicating 95% confidence intervals, shown separately for amphibians, reptiles, birds, and mammals. Black diamonds indicate the mean effect size of each predictor across clades within each tetrapod class. Colours denote effect direction and statistical significance: blue, positive and significant; red, negative and significant; green, non-significant (confidence intervals overlapping zero). The data underlying this figure can be found in <https://doi.org/10.5281/zenodo.14008084>


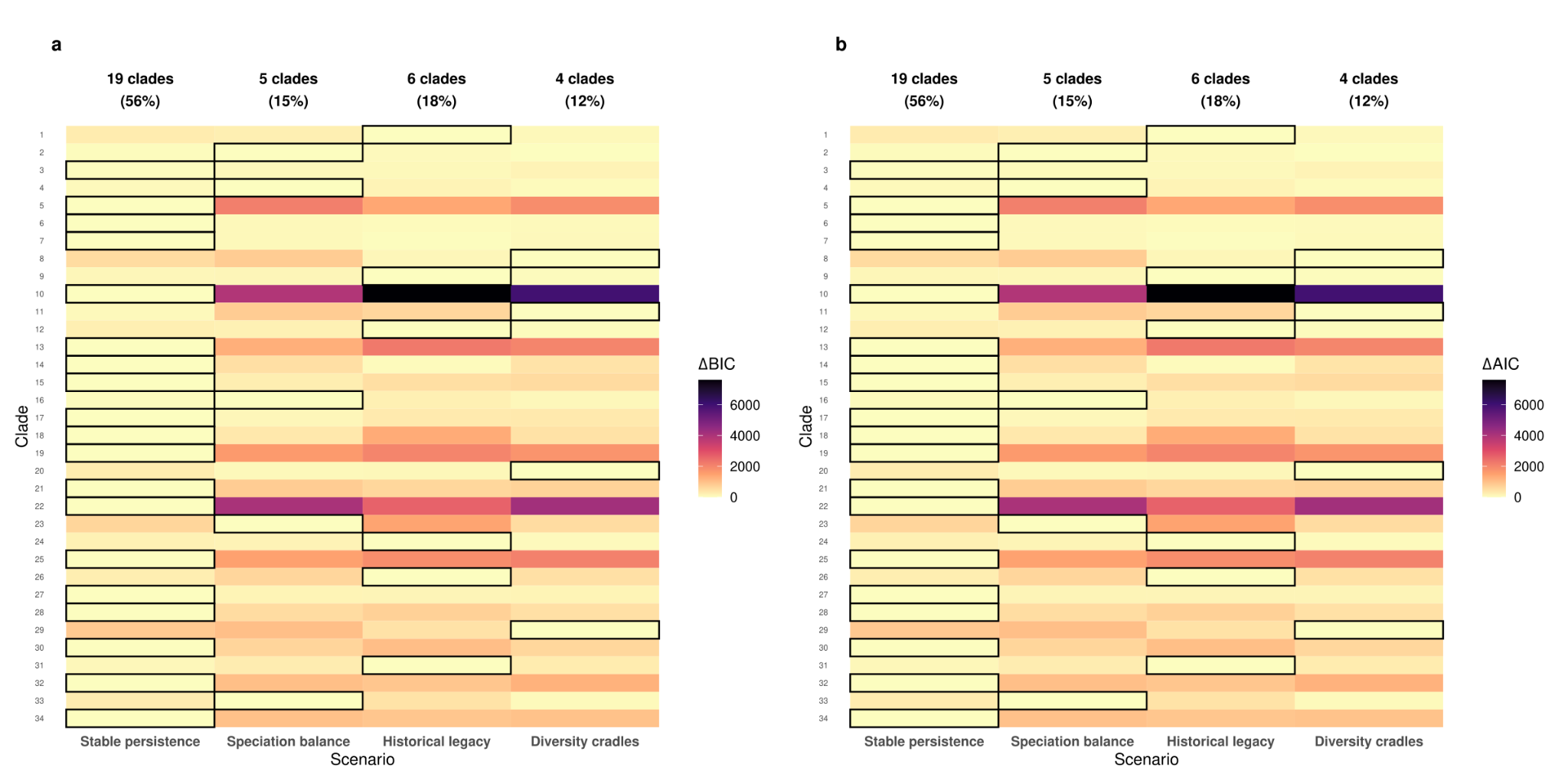


**Fig S. Model-selection support for alternative richness-generating scenarios across amphibian clades based on information-theoretic criteria.** Heatmaps show (a) ΔBIC and (b) ΔAIC values for four scenario-specific path models (stable persistence, historical legacy, diversity cradles, and speciation balance) evaluated separately for each clade (rows). Lower Δ values indicate stronger relative model support within a clade. For each clade, the scenario with the lowest information-criterion value is outlined with a darker border, indicating the most strongly supported model. Colors represent relative ΔBIC or ΔAIC values within clades. The data underlying this figure can be found in <https://doi.org/10.5281/zenodo.14008084>


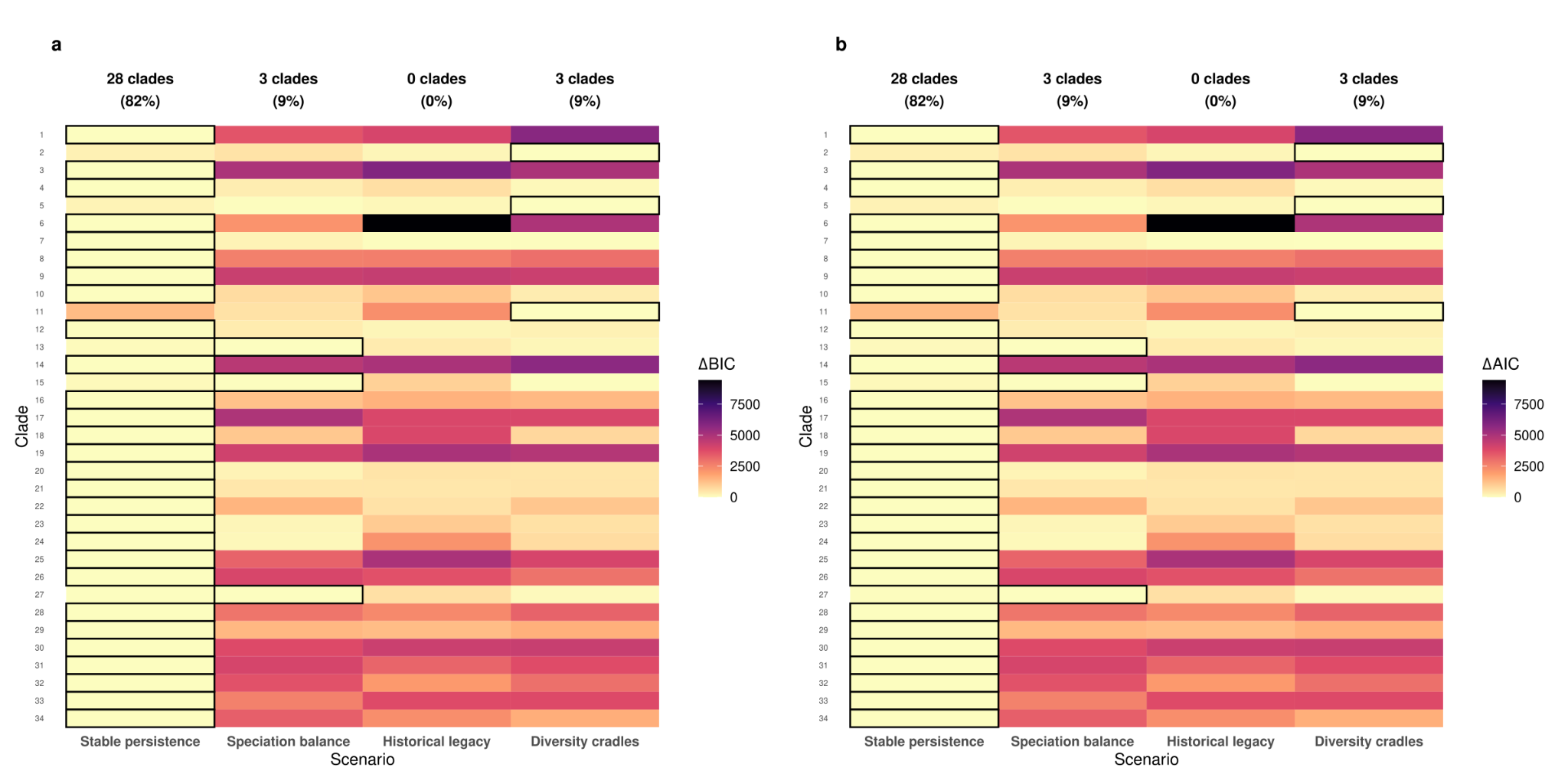


**Fig T. Model-selection support for alternative richness-generating scenarios across reptile clades based on information-theoretic criteria.** Heatmaps show (a) ΔBIC and (b) ΔAIC values for four scenario-specific path models (stable persistence, historical legacy, diversity cradles, and speciation balance) evaluated separately for each clade (rows). Lower Δ values indicate stronger relative model support within a clade. For each clade, the scenario with the lowest information-criterion value is outlined with a darker border, indicating the most strongly supported model. Colors represent relative ΔBIC or ΔAIC values within clades. The data underlying this figure can be found in <https://doi.org/10.5281/zenodo.14008084>


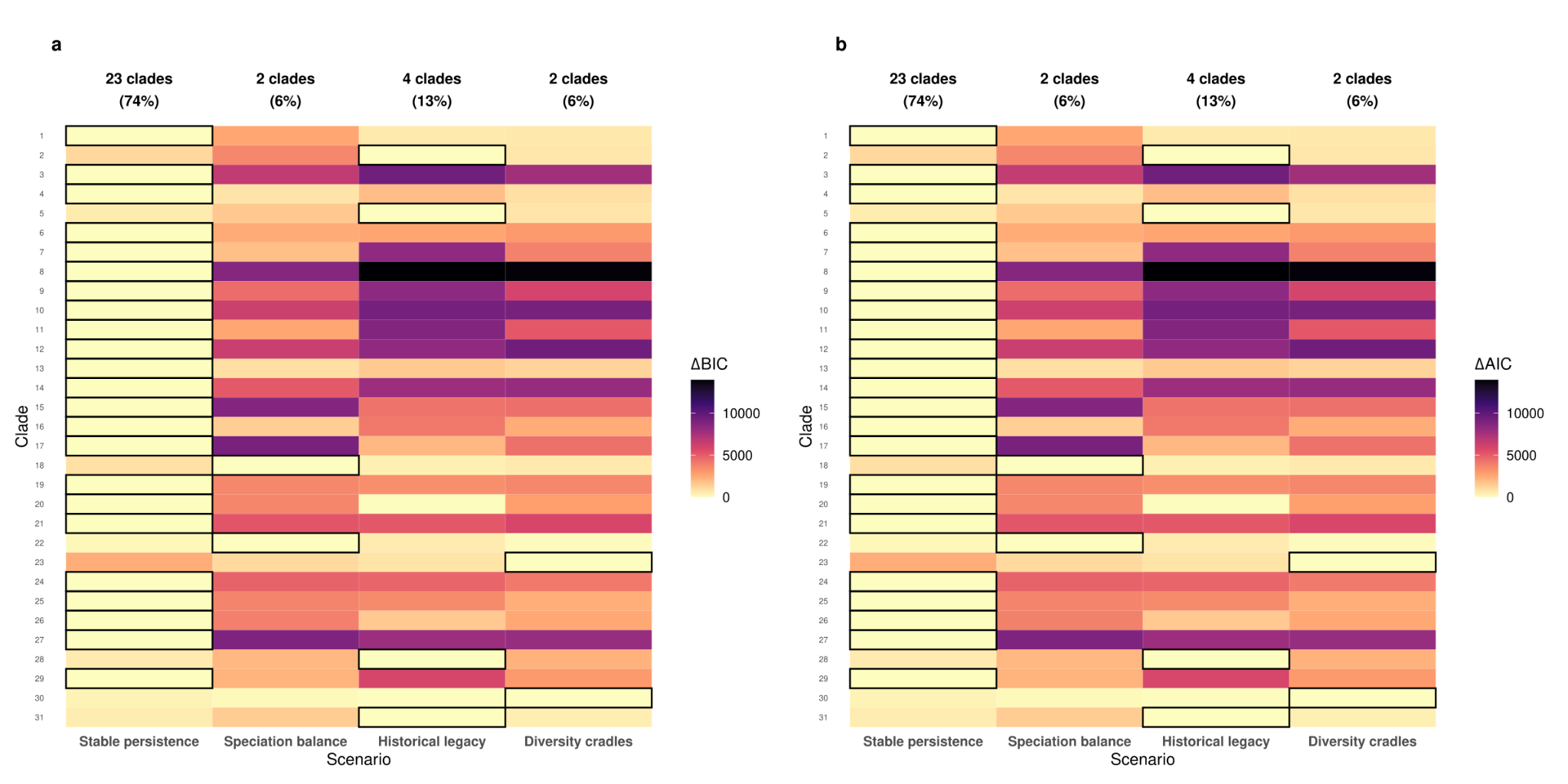


**Fig U. Model-selection support for alternative richness-generating scenarios across bird clades based on information-theoretic criteria.** Heatmaps show (a) ΔBIC and (b) ΔAIC values for four scenario-specific path models (stable persistence, historical legacy, diversity cradles, and speciation balance) evaluated separately for each clade (rows). Lower Δ values indicate stronger relative model support within a clade. For each clade, the scenario with the lowest information-criterion value is outlined with a darker border, indicating the most strongly supported model. Colors represent relative ΔBIC or ΔAIC values within clades. The data underlying this figure can be found in <https://doi.org/10.5281/zenodo.14008084>


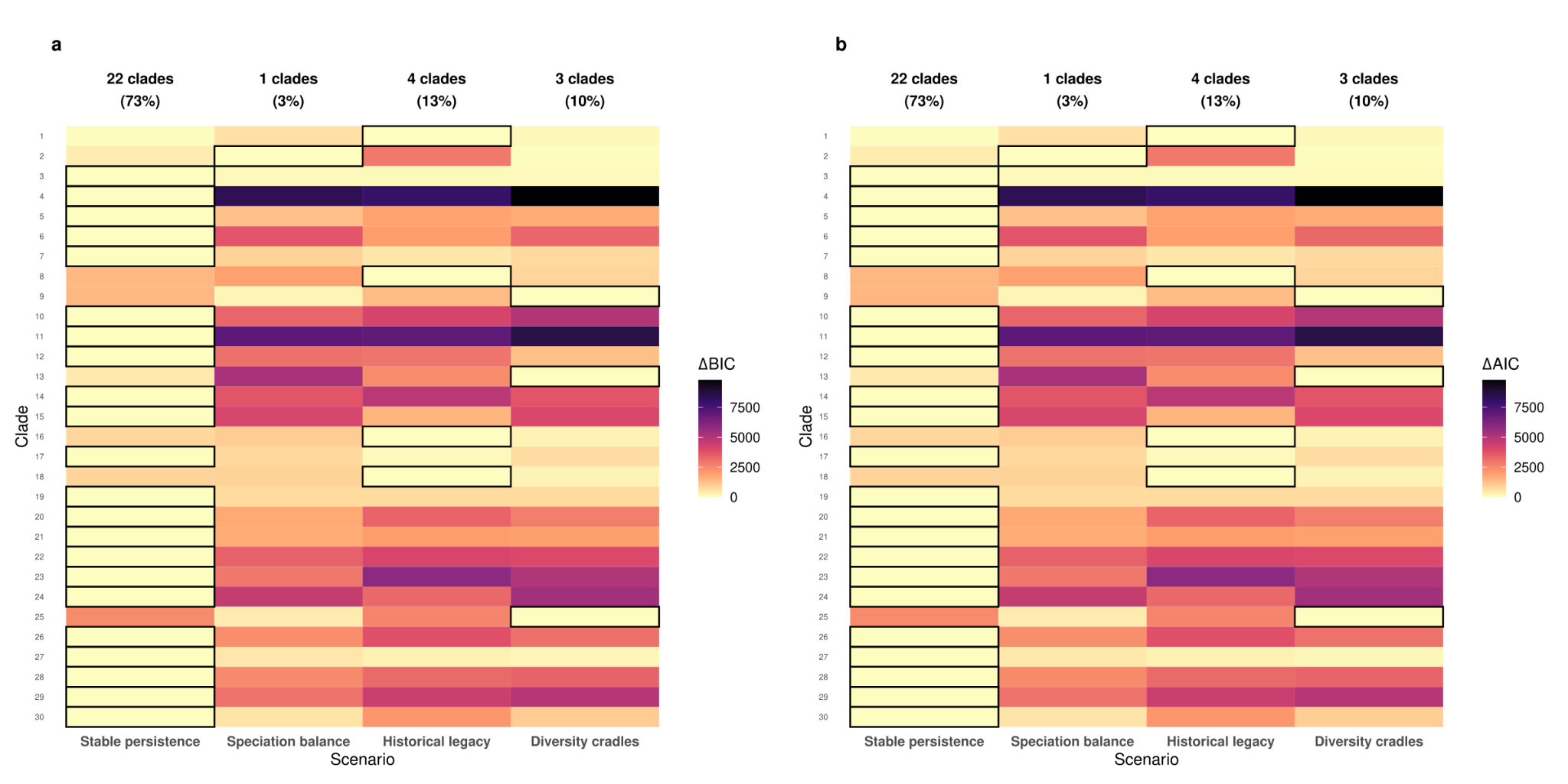


**Fig V. Model-selection support for alternative richness-generating scenarios across mammal clades based on information-theoretic criteria.** Heatmaps show (a) ΔBIC and (b) ΔAIC values for four scenario-specific path models (stable persistence, historical legacy, diversity cradles, and speciation balance) evaluated separately for each clade (rows). Lower Δ values indicate stronger relative model support within a clade. For each clade, the scenario with the lowest information-criterion value is outlined with a darker border, indicating the most strongly supported model. Colors represent relative ΔBIC or ΔAIC values within clades. The data underlying this figure can be found in <https://doi.org/10.5281/zenodo.14008084>


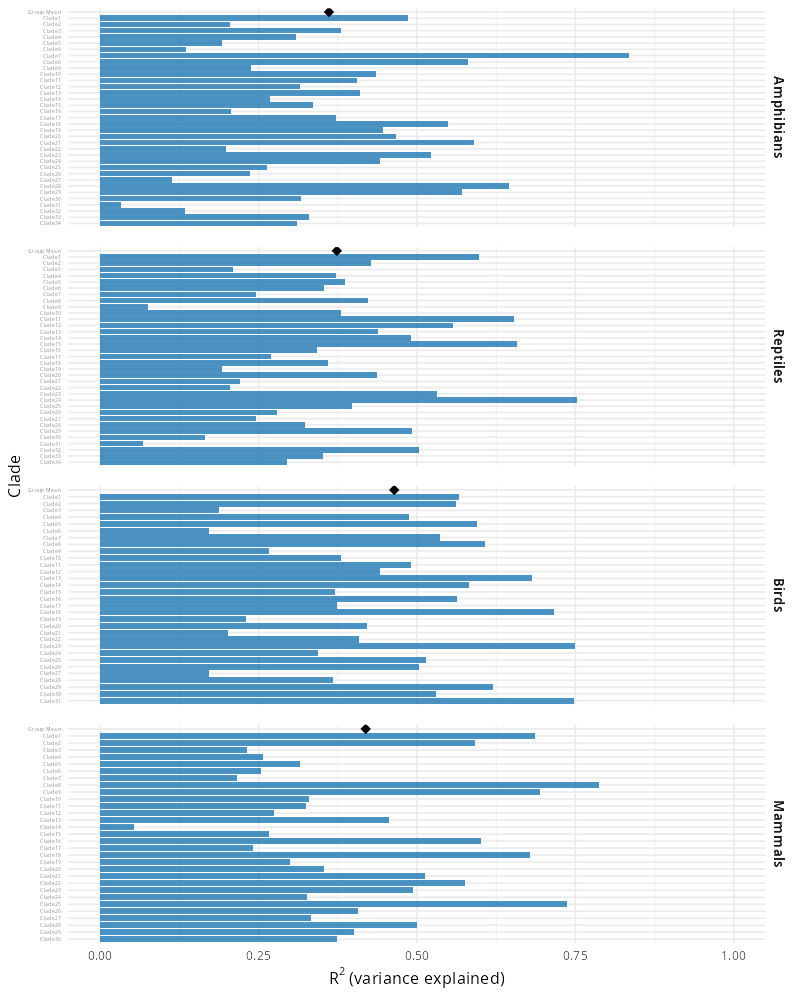


**Fig W. Variance explained (R²) by clade-specific path models across tetrapods.** Bars represent R² values for individual clades, reflecting the combined contribution of environmental, evolutionary time, and speciation rates predictors. Diamonds indicate mean R² values for each tetrapod class. The data underlying this figure can be found in <https://doi.org/10.5281/zenodo.14008084>


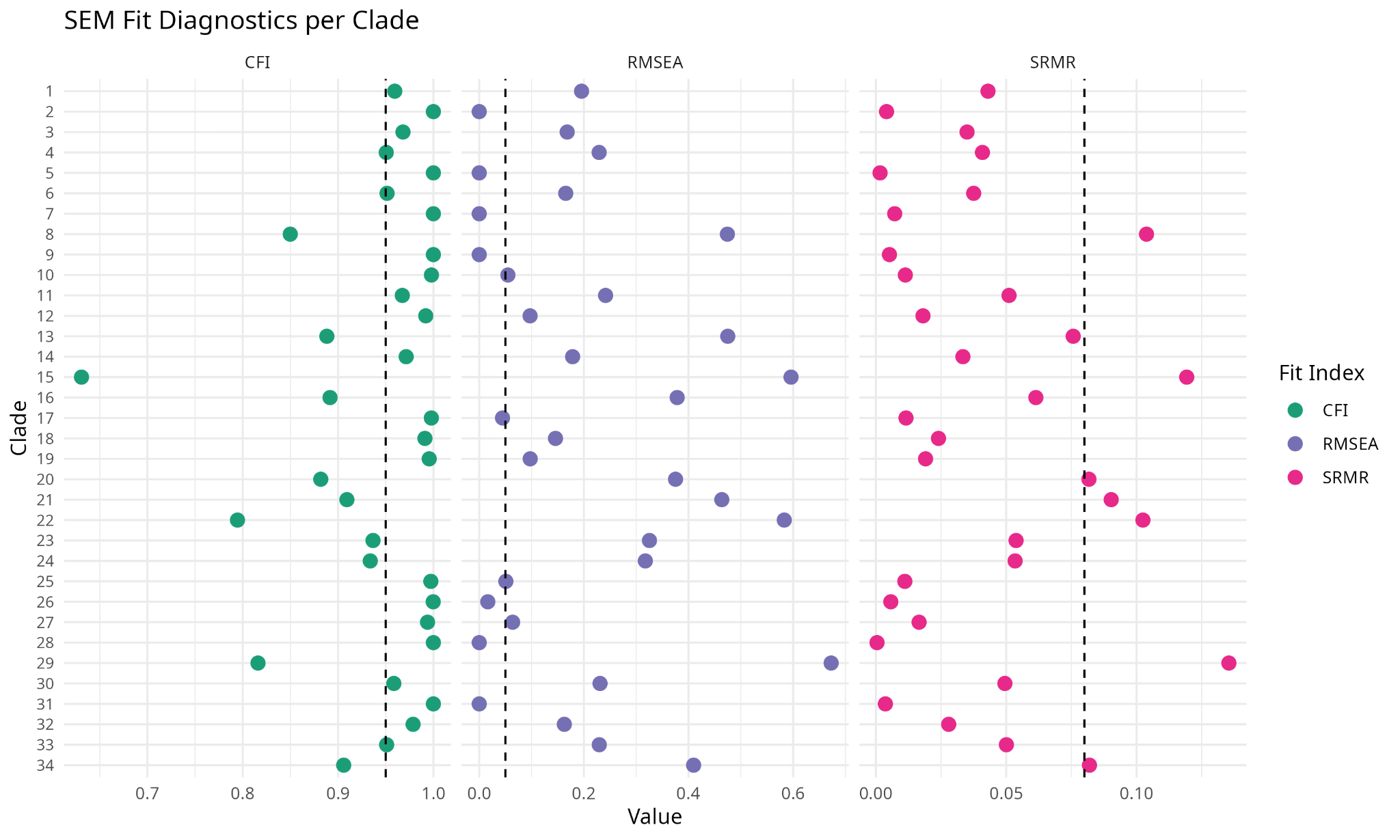


**Fig X. Model fit statistics for clade-specific path models across amphibians.** Distributions of goodness-of-fit indices for path models fitted separately to each clade, including the Comparative Fit Index (CFI), Root Mean Square Error of Approximation (RMSEA), and Standardized Root Mean Square Residual (SRMR). Each point represents the fit value for a given clade. Horizontal reference lines indicate commonly used thresholds for acceptable fit (CFI ≥ 0.95, RMSEA ≤ 0.05, SRMR ≤ 0.08). Most models meet or exceed recommended criteria for CFI and SRMR, whereas RMSEA values are frequently elevated, a pattern expected in large-sample and complex macroecological path models. The data underlying this figure can be found in <https://doi.org/10.5281/zenodo.14008084>


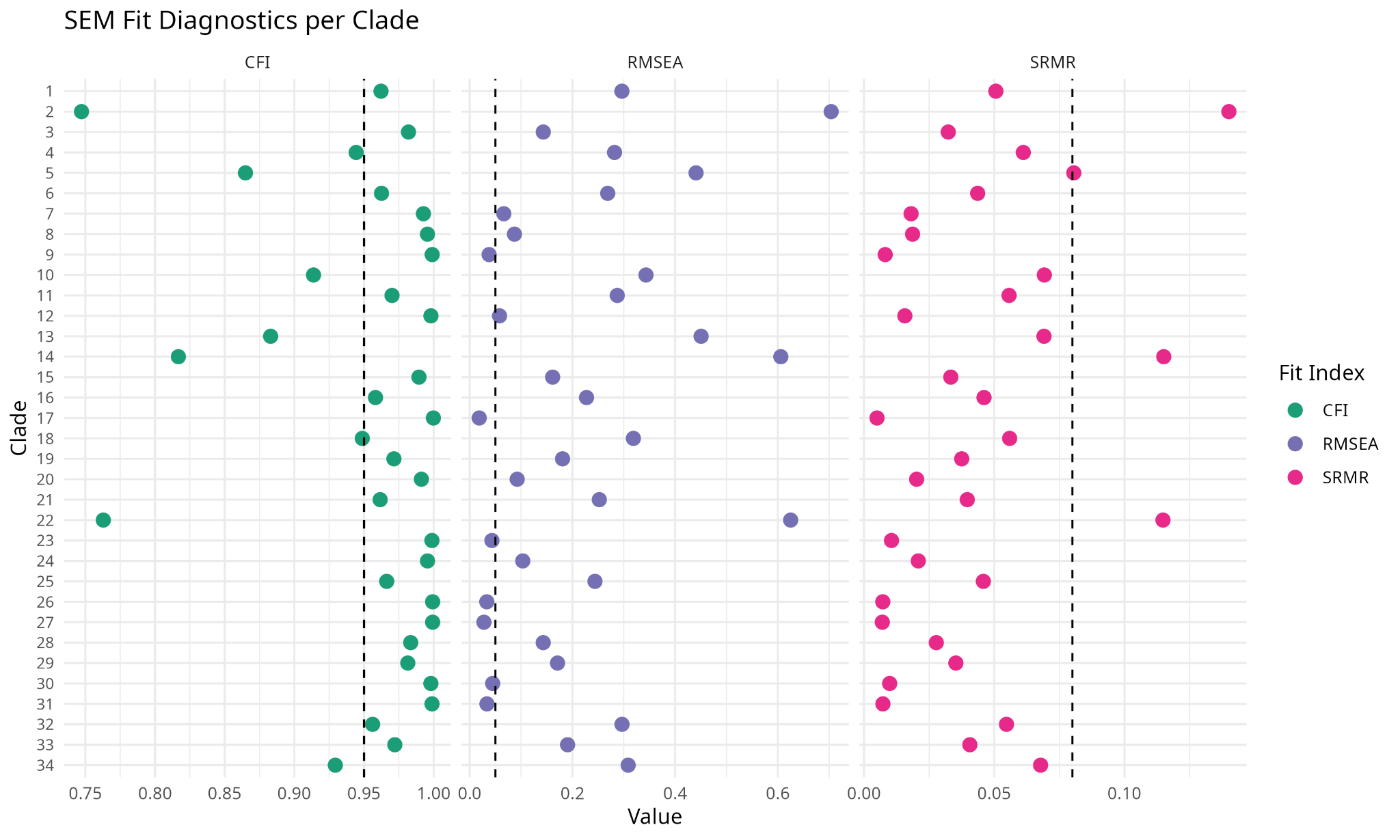


**Fig Y. Model fit statistics for clade-specific path models across reptiles.** Distributions of goodness-of-fit indices for path models fitted separately to each clade, including the Comparative Fit Index (CFI), Root Mean Square Error of Approximation (RMSEA), and Standardized Root Mean Square Residual (SRMR). Each point represents the fit value for a given clade. Horizontal reference lines indicate commonly used thresholds for acceptable fit (CFI ≥ 0.95, RMSEA ≤ 0.05, SRMR ≤ 0.08). Most models meet or exceed recommended criteria for CFI and SRMR, whereas RMSEA values are frequently elevated, a pattern expected in large-sample and complex macroecological path models. The data underlying this figure can be found in <https://doi.org/10.5281/zenodo.14008084>


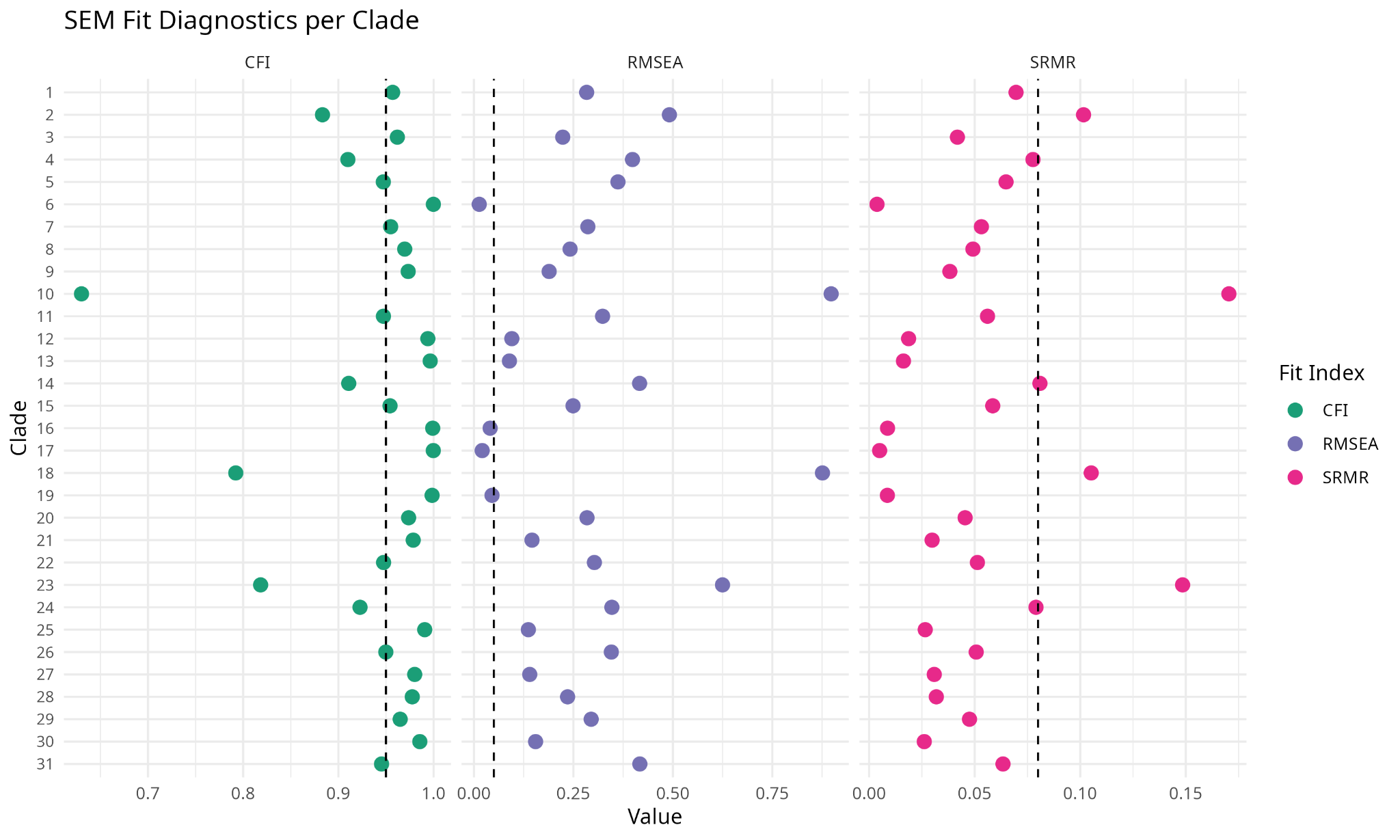


**Fig Z. Model fit statistics for clade-specific path models across birds.** Distributions of goodness-of-fit indices for path models fitted separately to each clade, including the Comparative Fit Index (CFI), Root Mean Square Error of Approximation (RMSEA), and Standardized Root Mean Square Residual (SRMR). Each point represents the fit value for a given clade. Horizontal reference lines indicate commonly used thresholds for acceptable fit (CFI ≥ 0.95, RMSEA ≤ 0.05, SRMR ≤ 0.08). Most models meet or exceed recommended criteria for CFI and SRMR, whereas RMSEA values are frequently elevated, a pattern expected in large-sample and complex macroecological path models. The data underlying this figure can be found in <https://doi.org/10.5281/zenodo.14008084>


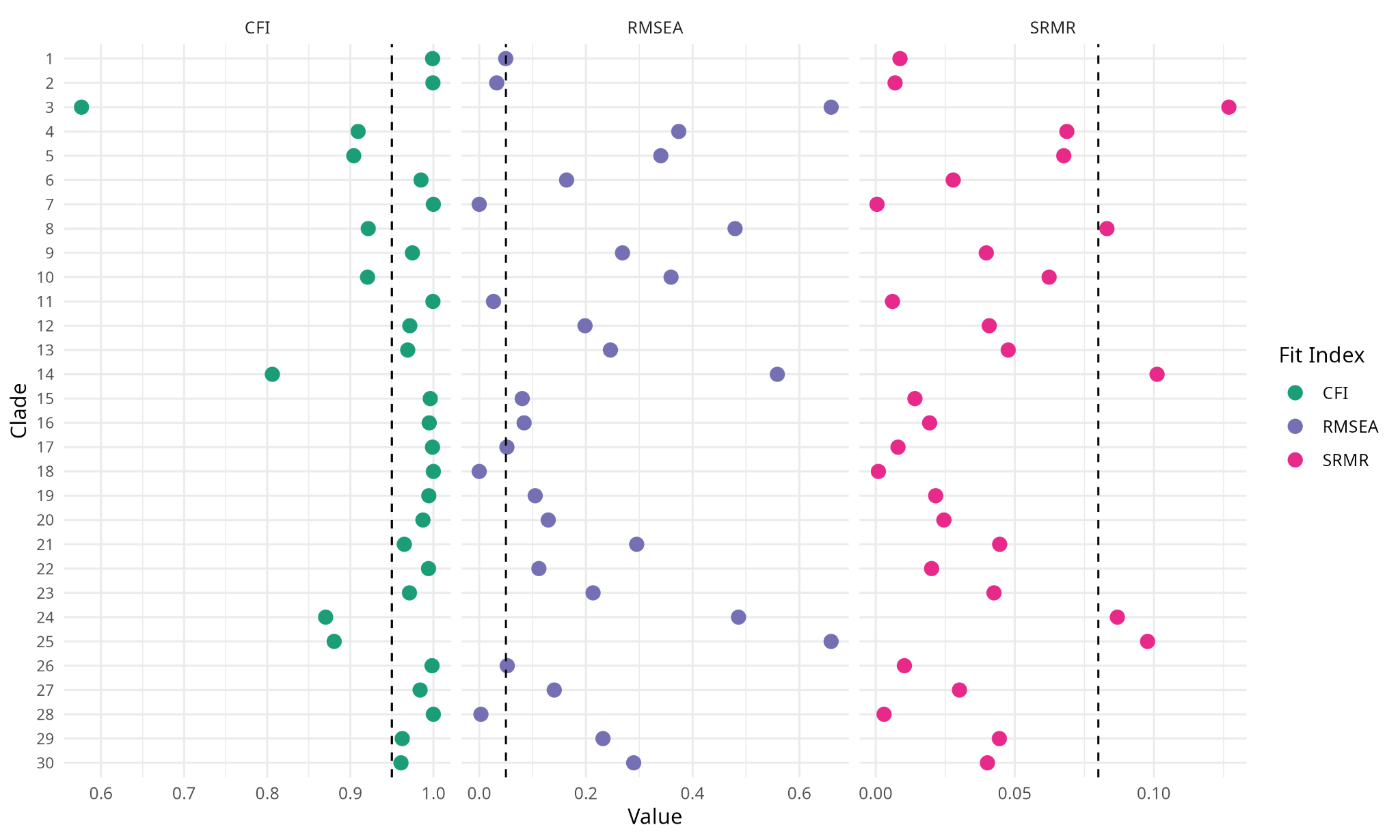


**Fig AA. Model fit statistics for clade-specific path models across mammals.** Distributions of goodness-of-fit indices for path models fitted separately to each clade, including the Comparative Fit Index (CFI), Root Mean Square Error of Approximation (RMSEA), and Standardized Root Mean Square Residual (SRMR). Each point represents the fit value for a given clade. Horizontal reference lines indicate commonly used thresholds for acceptable fit (CFI ≥ 0.95, RMSEA ≤ 0.05, SRMR ≤ 0.08). Most models meet or exceed recommended criteria for CFI and SRMR, whereas RMSEA values are frequently elevated, a pattern expected in large-sample and complex macroecological path models. The data underlying this figure can be found in <https://doi.org/10.5281/zenodo.14008084>


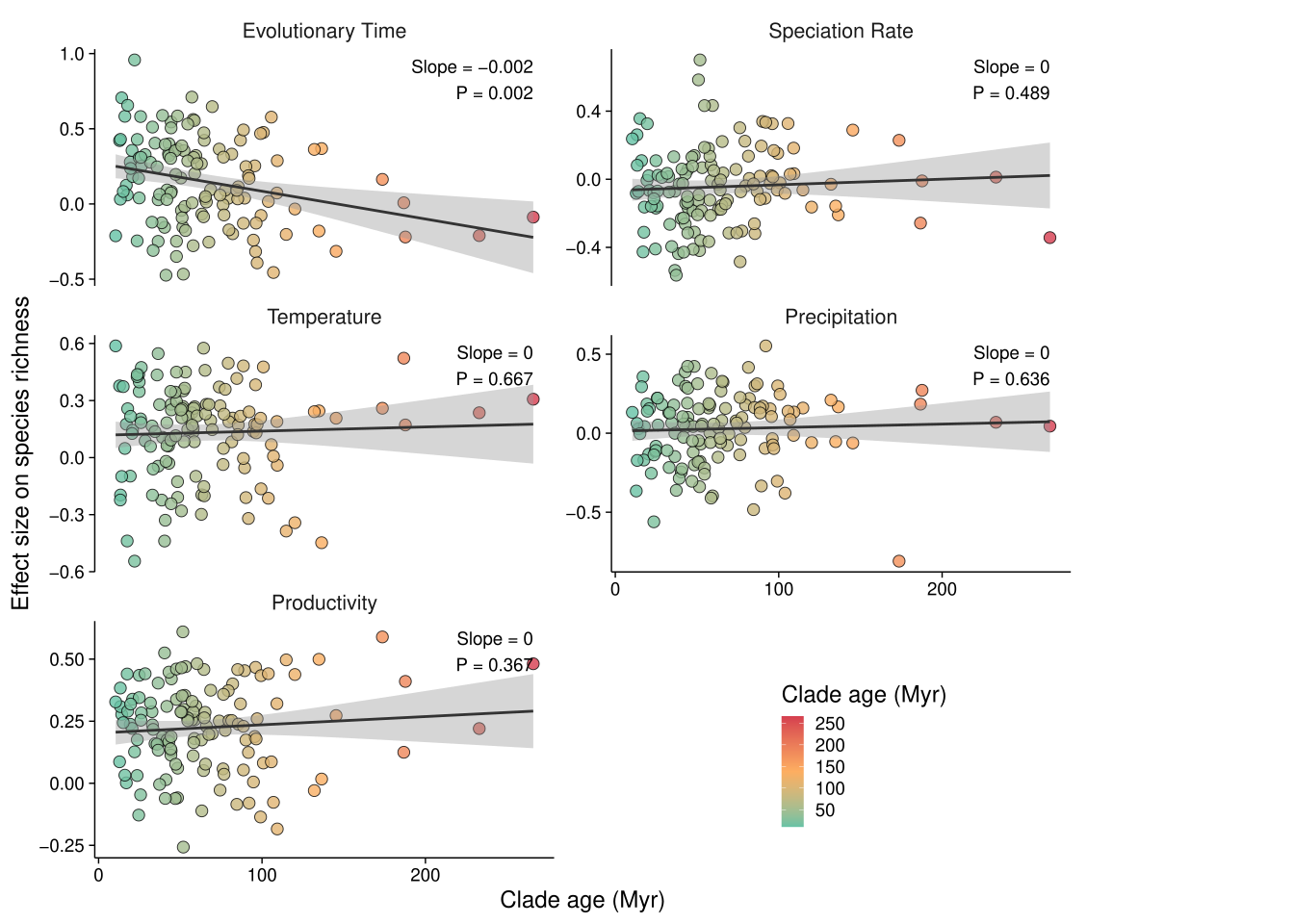


**Fig AB. Relationship between clade age and the strength of direct predictor effects on species richness across tetrapods.** Each point represents a clade, and lines show linear regressions between clade age and standardized effect sizes for environmental factors, evolutionary time, and speciation rates factors on species richness. The data underlying this figure can be found in <https://doi.org/10.5281/zenodo.14008084>


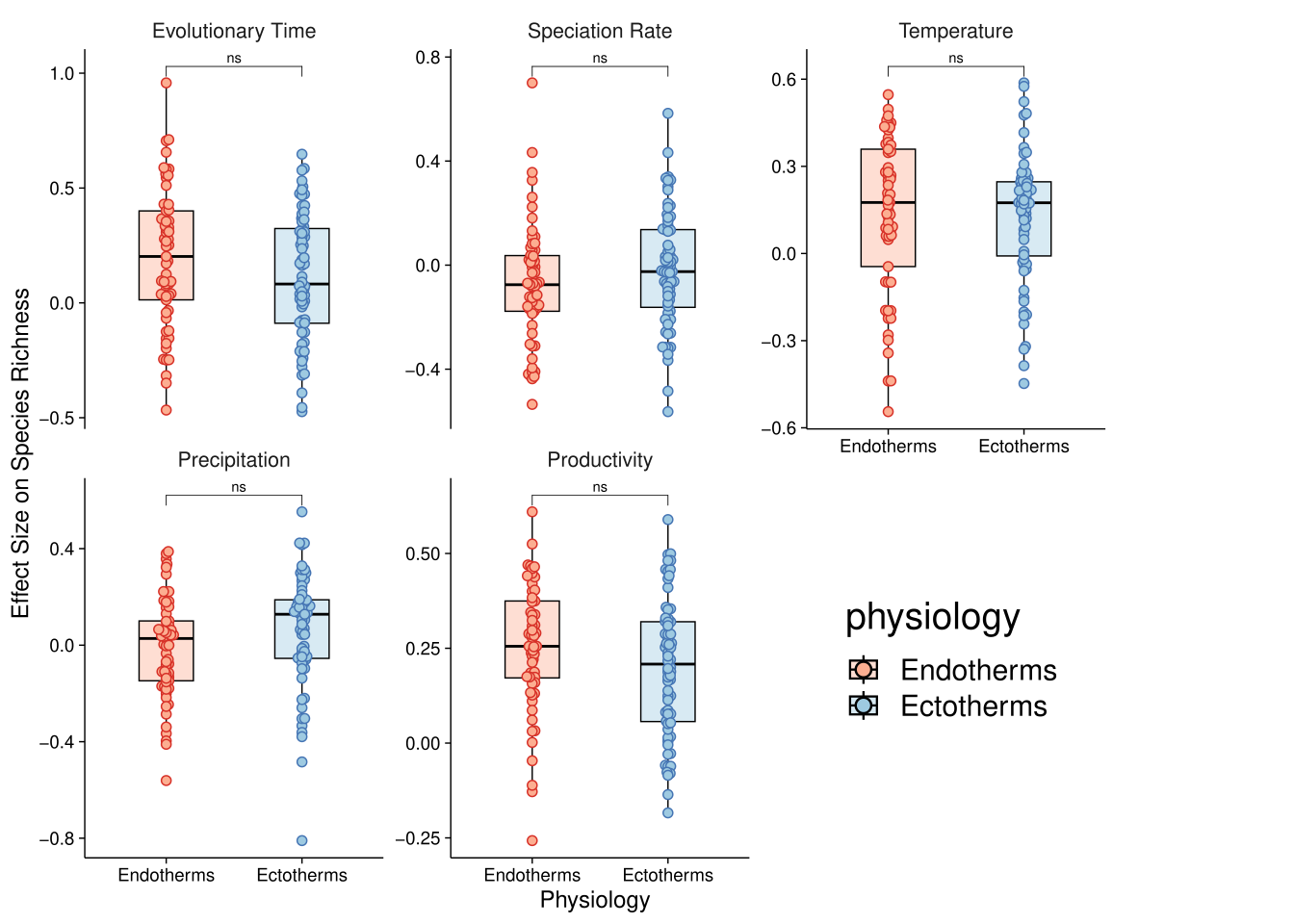


**Fig AC. Relationship between clade physiology and the strength of direct predictor effects on species richness across tetrapods.** Clades are classified as ectothermic or endothermic. Non-significant relationships are indicated by “ns”. The data underlying this figure can be found in <https://doi.org/10.5281/zenodo.14008084>


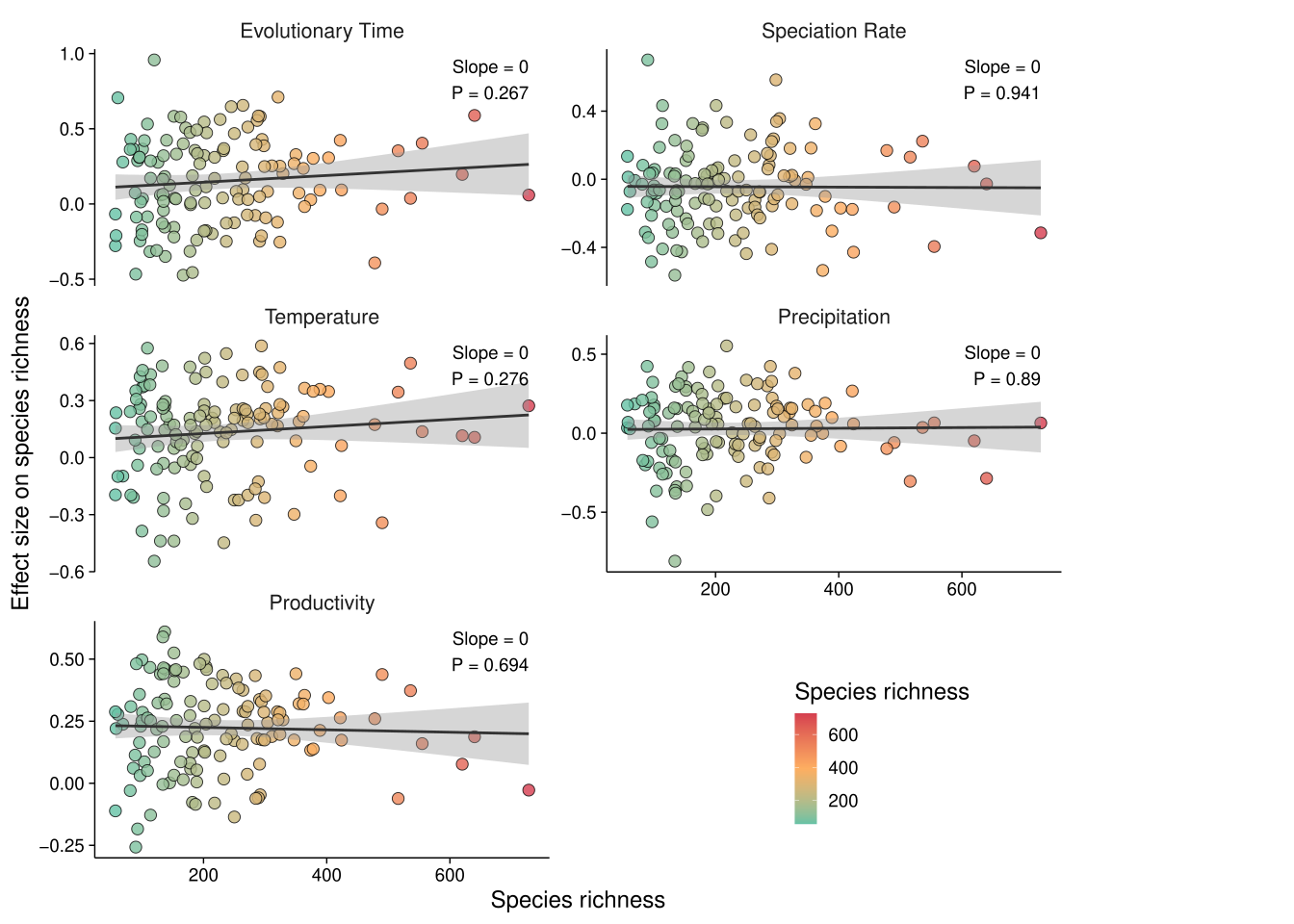


**Fig AD. Relationship between clade diversity (species richness) and the strength of direct predictor effects on species richness across tetrapods.** Each point represents a clade, and lines show linear regressions between clade diversity and standardized effect sizes for environmental factors, evolutionary time, and speciation rates factors on species richness. The data underlying this figure can be found in <https://doi.org/10.5281/zenodo.14008084>


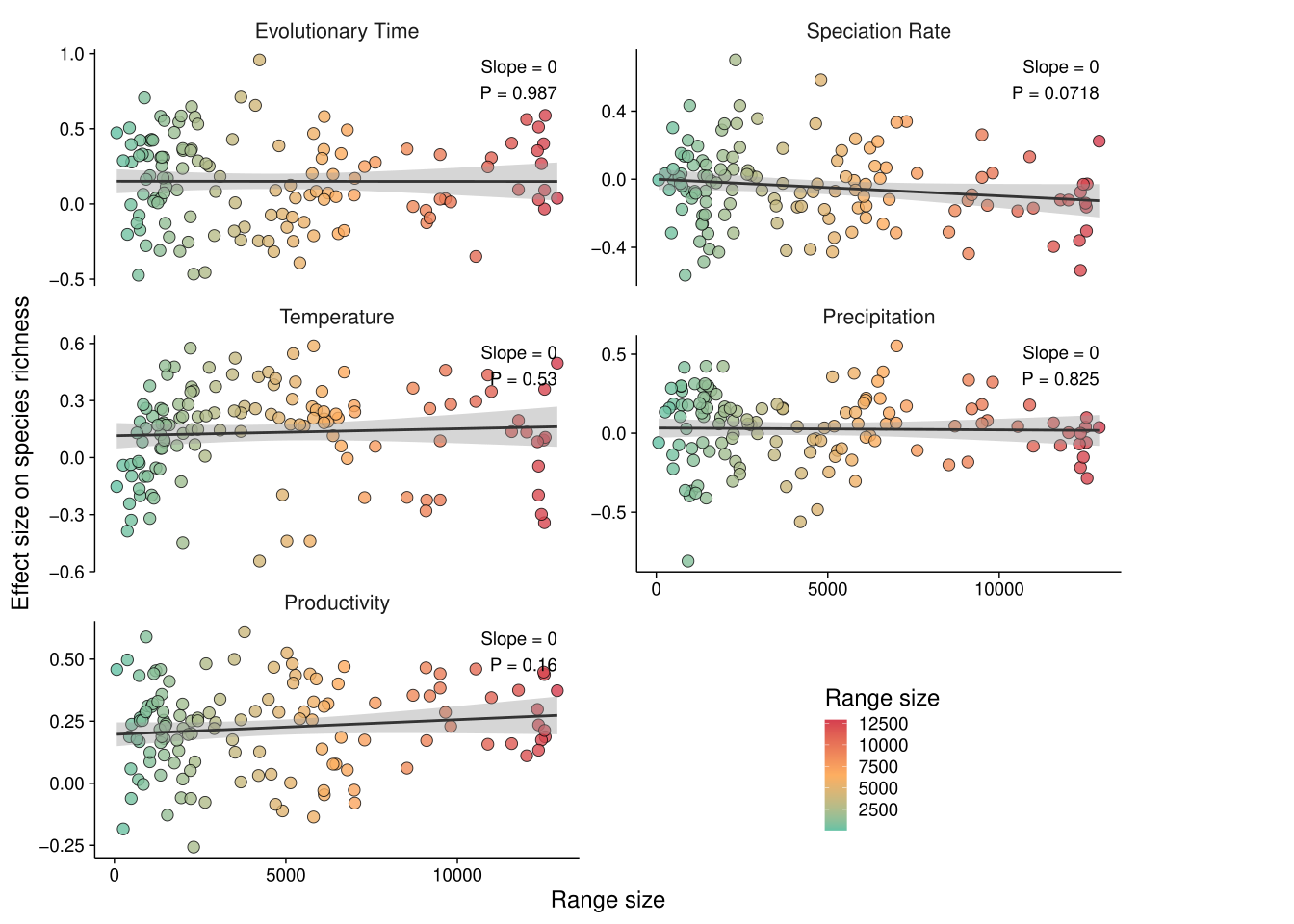


**Fig AE. Relationship between clade geographic extent (range size) and the strength of direct predictor effects on species richness across tetrapods.** Each point represents a clade, and lines show linear regressions between clade geographic extent and standardized effect sizes for environmental factors, evolutionary time, and speciation rates factors on species richness. The data underlying this figure can be found in <https://doi.org/10.5281/zenodo.14008084>


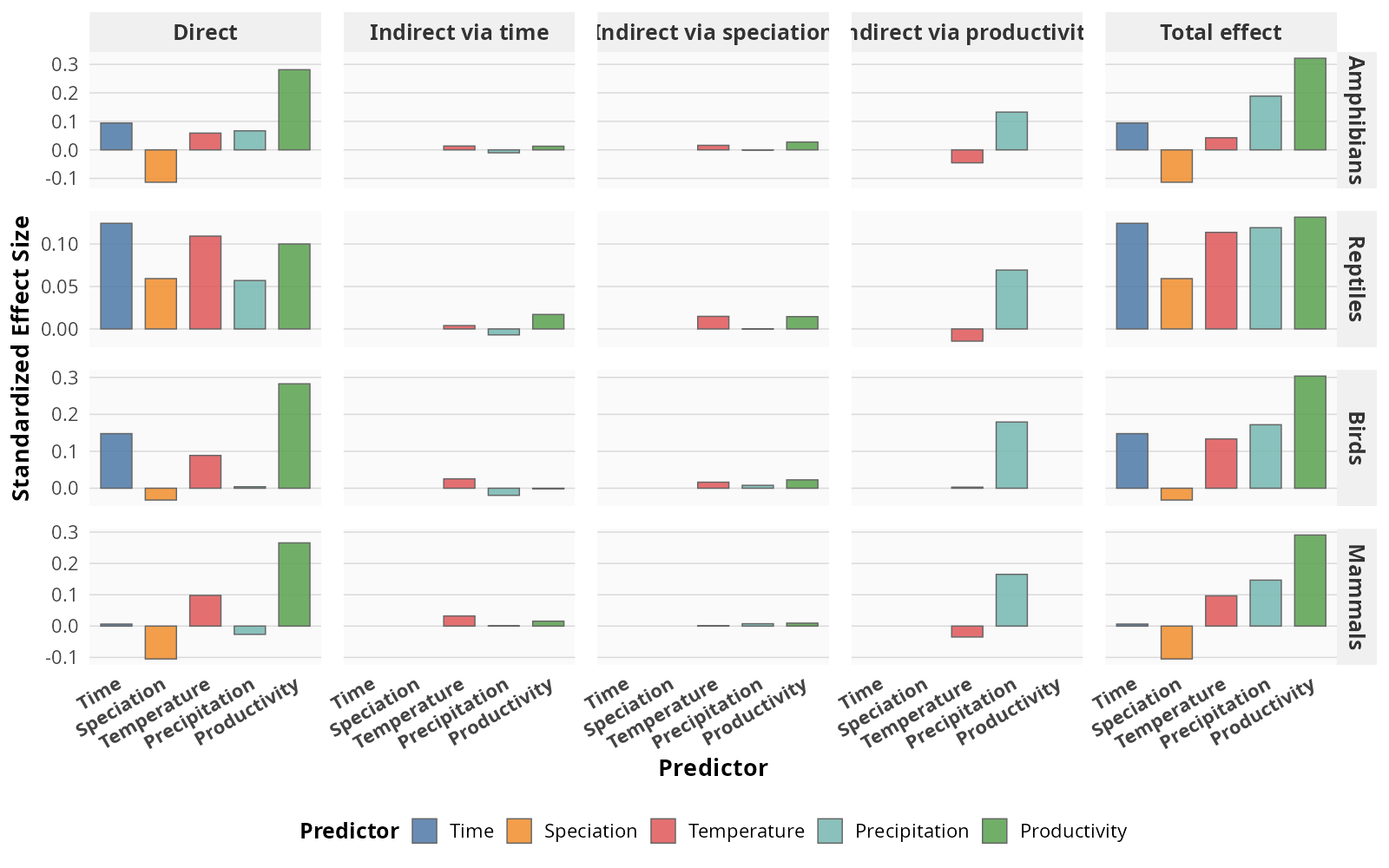


**Fig AF. Robustness of path-model results to the number of Laplacian modalities (40 modalities).** Bars show mean standardized path coefficients (β) representing direct, indirect, and total effects of five predictors on species richness across tetrapod clades identified using 40 modalities: amphibians (28 clades), reptiles (26 clades), birds (25 clades), and mammals (27 clades). Evolutionary time was approximated using mean pairwise phylogenetic distance (MPD) rather than assemblage arrival time, owing to the computational cost of historical biogeographic reconstructions. Other predictors include speciation rate (DR estimates), temperature, precipitation, and net primary productivity (NPP). Indirect effects are grouped into three classes: (i) environmental effects mediated through evolutionary time (MPD), (ii) environmental effects mediated through speciation rates, and (iii) climatic effects mediated through productivity. Total effects combine direct and all indirect pathways. This analysis evaluates whether reducing the number of modalities relative to the main analyses affects the overall conclusions. The data underlying this figure can be found in <https://doi.org/10.5281/zenodo.14008084>


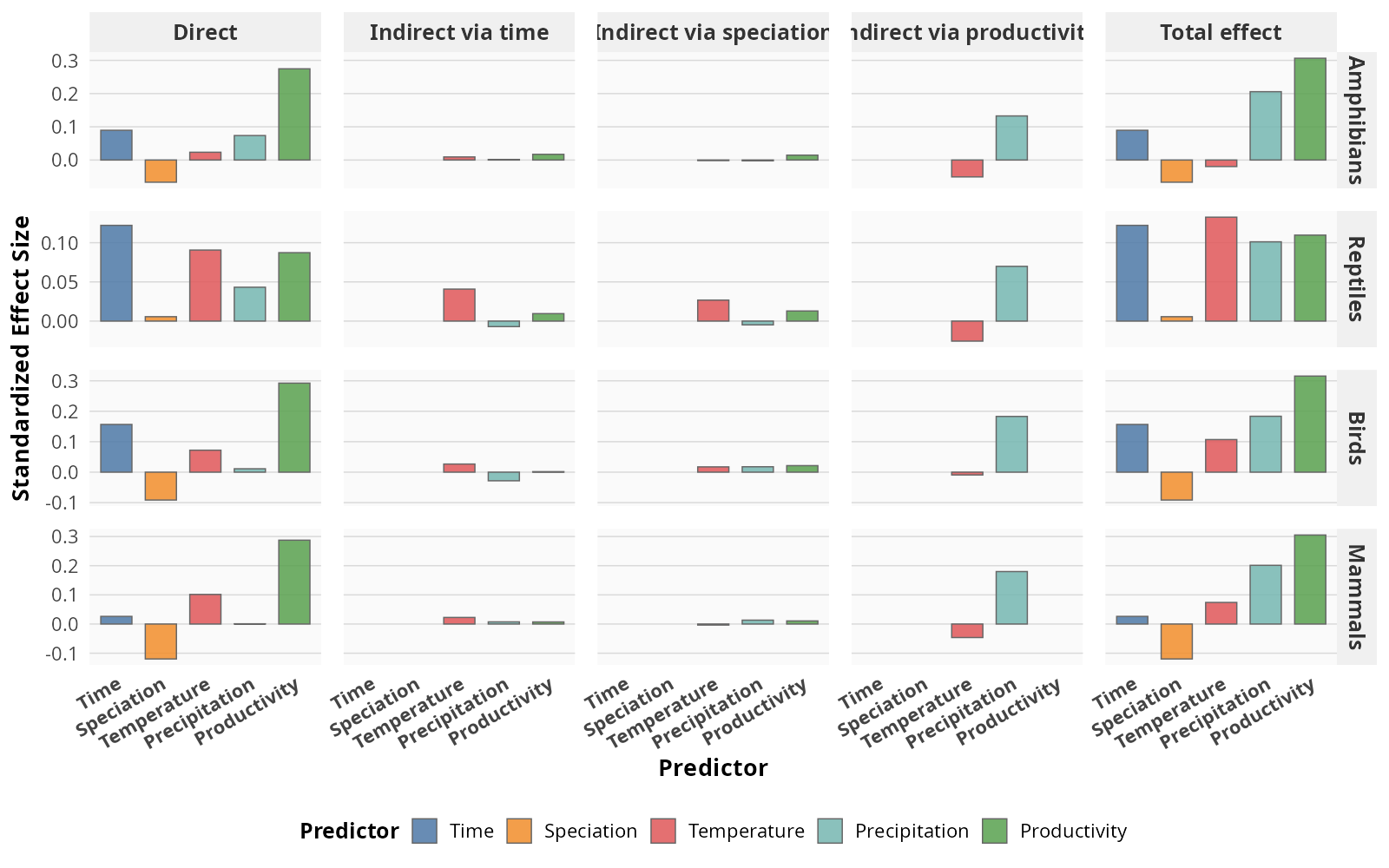


**Fig AG. Robustness of path-model results to the number of Laplacian modalities (60 modalities).** Bars show mean standardized path coefficients (β) representing direct, indirect, and total effects of five predictors on species richness across tetrapod clades identified using 60 modalities: amphibians (35 clades), reptiles (34 clades), birds (34 clades), and mammals (37 clades). Evolutionary time was approximated using mean pairwise phylogenetic distance (MPD) rather than assemblage arrival time, owing to the computational cost of historical biogeographic reconstructions. Other predictors include speciation rate (DR estimates), temperature, precipitation, and net primary productivity (NPP). Total effects combine direct and indirect pathways. This analysis assesses whether increasing the number of modalities alters the qualitative conclusions. The data underlying this figure can be found in <https://doi.org/10.5281/zenodo.14008084>


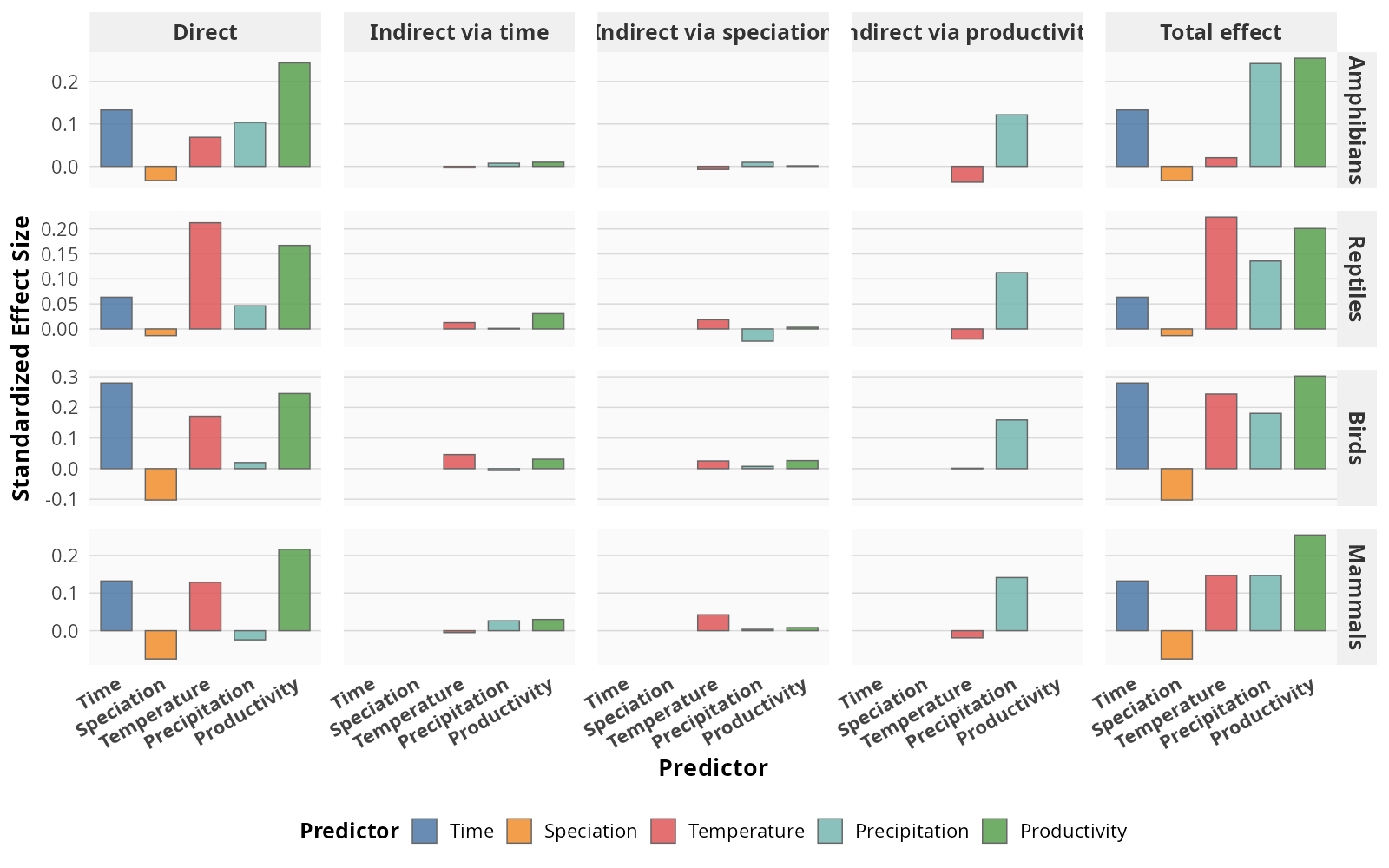


**Fig AH. Effects of environmental, evolutionary time, and speciation variables on species richness after excluding paraphyletic clades.** Bars show mean standardized path coefficients (β) representing direct, indirect, and total effects across amphibians (30 clades), reptiles (31 clades), birds (29 clades), and mammals (25 clades). Predictors include evolutionary time (assemblage arrival time), speciation rate (DR estimates), temperature, precipitation, and net primary productivity (NPP). Indirect effects are grouped into three classes: (i) environmental effects mediated via evolutionary time, (ii) environmental effects mediated via speciation rates, and (iii) climatic effects mediated via productivity. Colours correspond to predictor variables included in the path models. The data underlying this figure can be found in <https://doi.org/10.5281/zenodo.14008084>


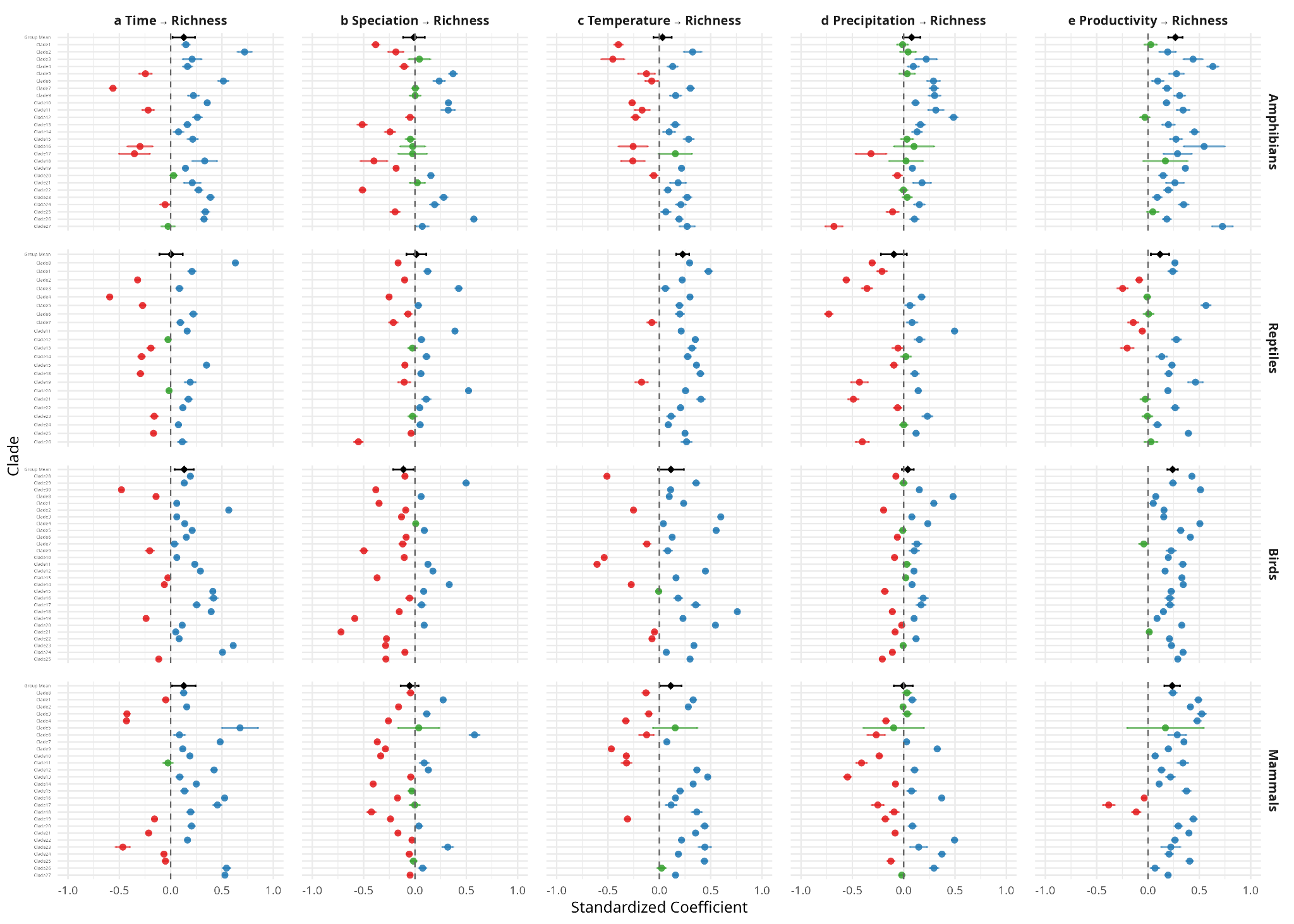


**Fig AI. Direct effects of environmental, evolutionary time, and speciation variables on species richness estimated using molecular-only phylogenies.** Clades were delineated using Laplacian spectral clustering applied to phylogenies restricted to species with genetic data only. Path models corresponding to Fig 3 were refitted for each clade, and mean standardized coefficients were extracted across clades for amphibians, reptiles, birds, and mammals. Points represent means and error bars indicate 95% confidence intervals. Colours denote effect direction and significance: blue, positive and significant; red, negative and significant; green, non-significant (confidence intervals overlapping zero). The data underlying this figure can be found in <https://doi.org/10.5281/zenodo.14008084>


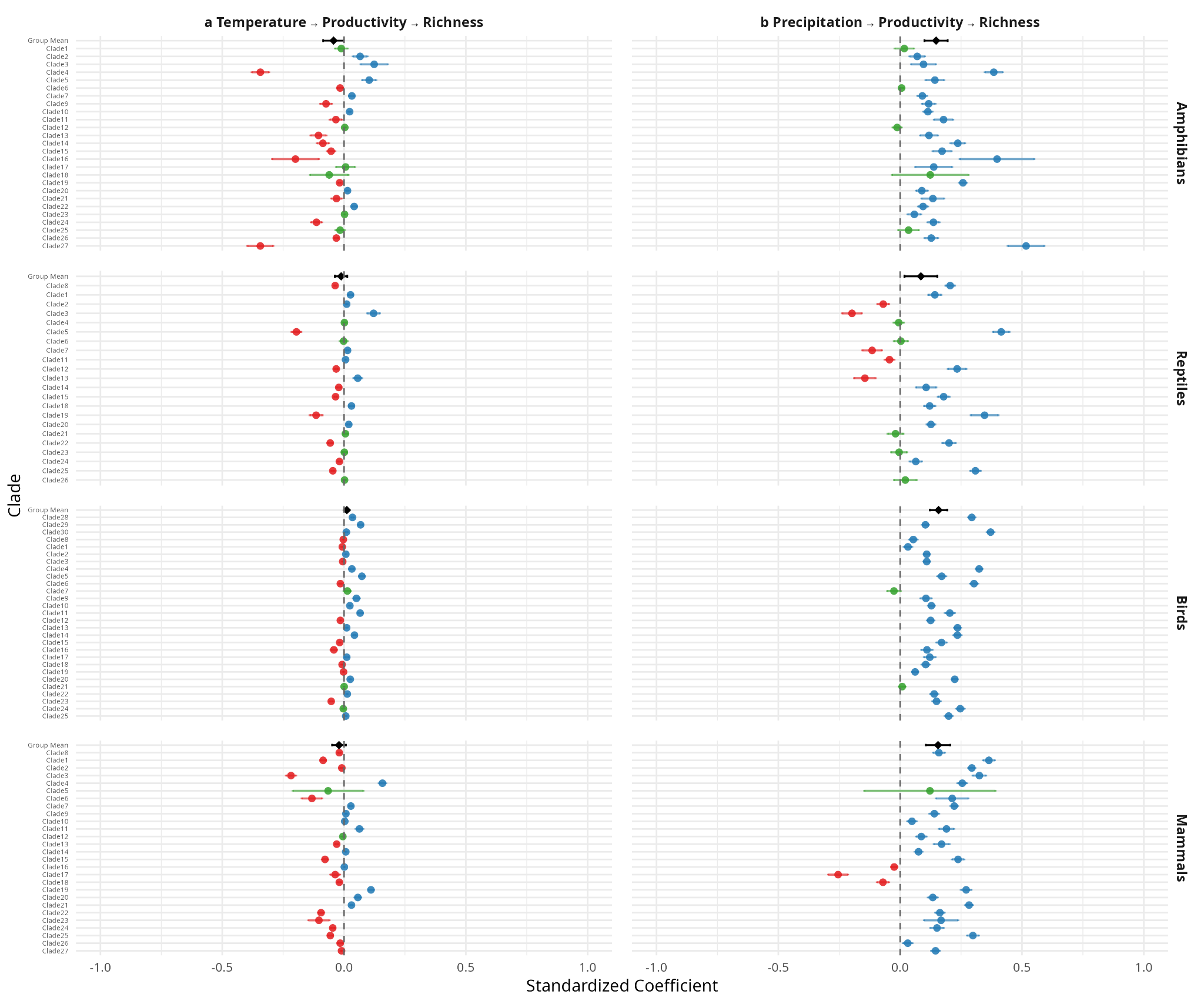


**Fig AJ. Indirect effects of climate (temperature and precipitation) on species richness mediated through productivity, estimated using molecular-only phylogenies.** Clades were delineated using Laplacian spectral clustering applied to phylogenies restricted to genetically sampled species. For each clade, the path model shown in Fig 3 was fitted and standardized coefficients were extracted. Points represent mean effects across clades for each tetrapod class, with error bars indicating 95% confidence intervals. Colours denote effect direction and significance: blue, positive and significant; red, negative and significant; green, non-significant (confidence intervals overlapping zero). The data underlying this figure can be found in <https://doi.org/10.5281/zenodo.14008084>


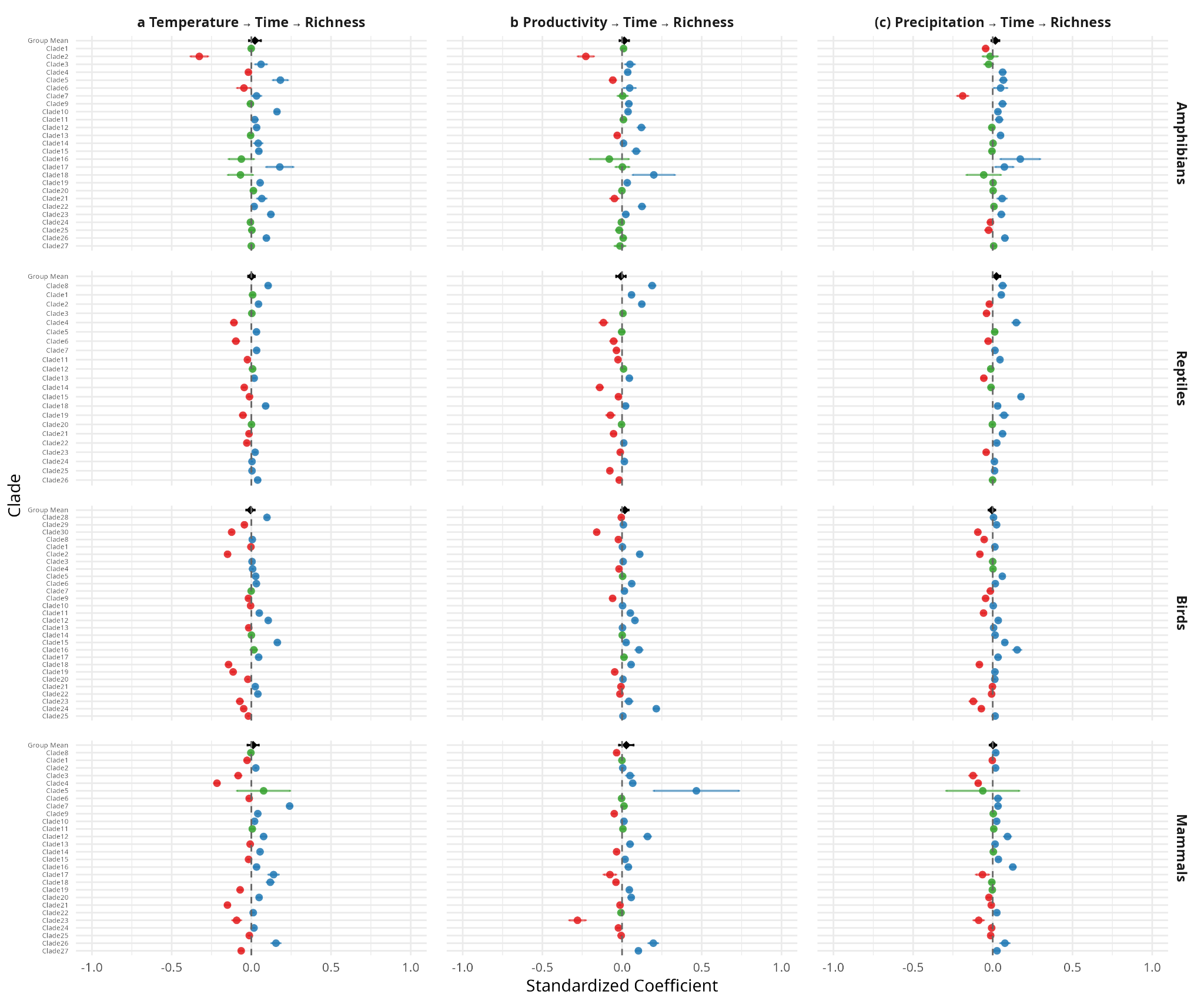


**Fig AK. Indirect effects of environmental variables mediated through evolutionary time (assemblage age) on species richness, estimated using molecular-only phylogenies.** Clades were delineated using Laplacian spectral clustering applied to phylogenies restricted to genetically sampled species. For each clade, the path model shown in Fig 3 was fitted and standardized coefficients were extracted. Points represent mean effects across clades for each tetrapod class, with error bars indicating 95% confidence intervals. Colours denote effect direction and significance: blue, positive and significant; red, negative and significant; green, non-significant (confidence intervals overlapping zero). The data underlying this figure can be found in <https://doi.org/10.5281/zenodo.14008084>


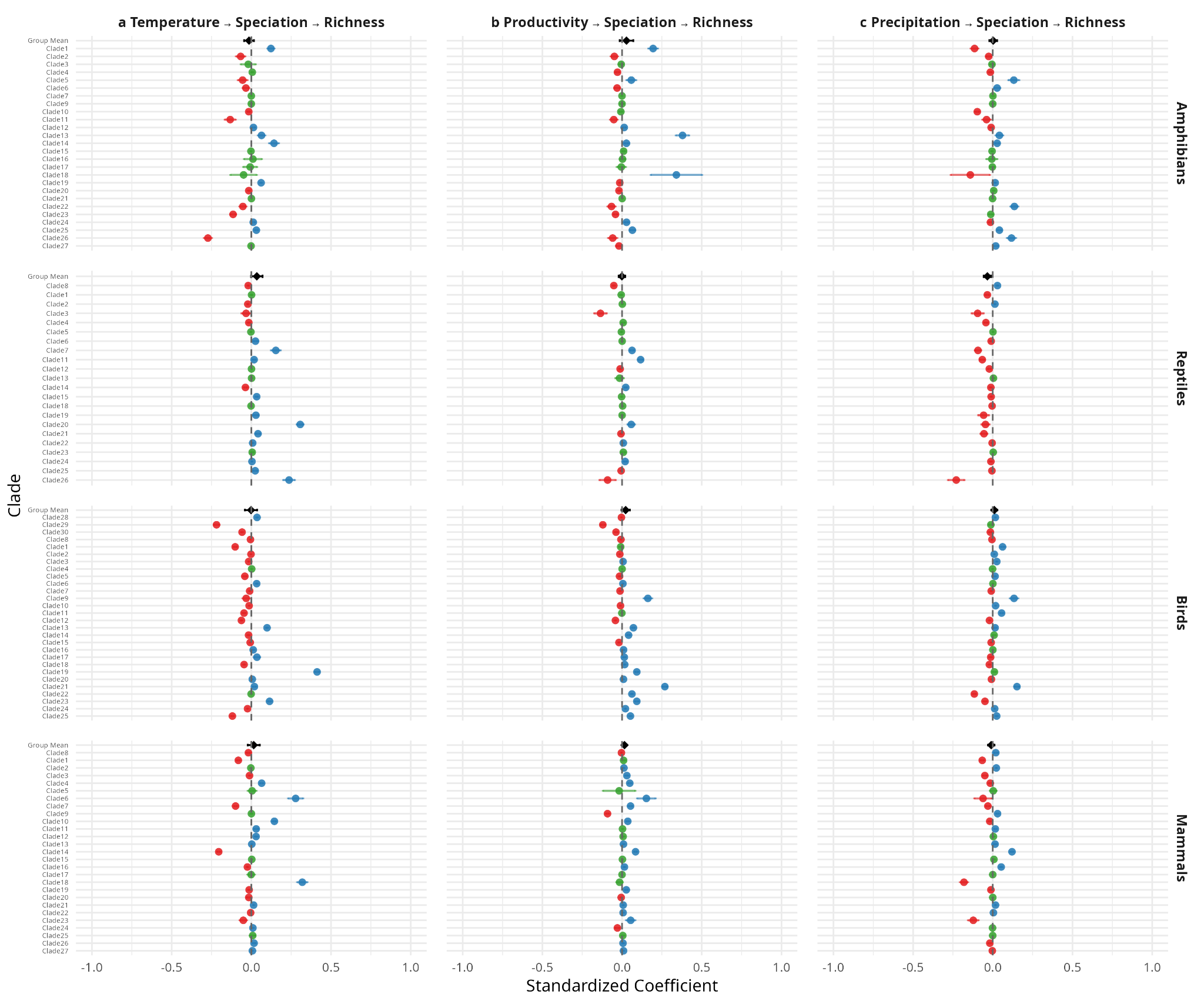


**Fig AL. Indirect effects of environmental variables mediated through speciation rates on species richness, estimated using molecular-only phylogenies.** Clades were delineated using Laplacian spectral clustering applied to phylogenies restricted to genetically sampled species. For each clade, the path model shown in Fig 3 was fitted and standardized coefficients were extracted. Points represent mean effects across clades for each tetrapod class, with error bars indicating 95% confidence intervals. Colours denote effect direction and significance: blue, positive and significant; red, negative and significant; green, non-significant (confidence intervals overlapping zero). The data underlying this figure can be found in <https://doi.org/10.5281/zenodo.14008084>


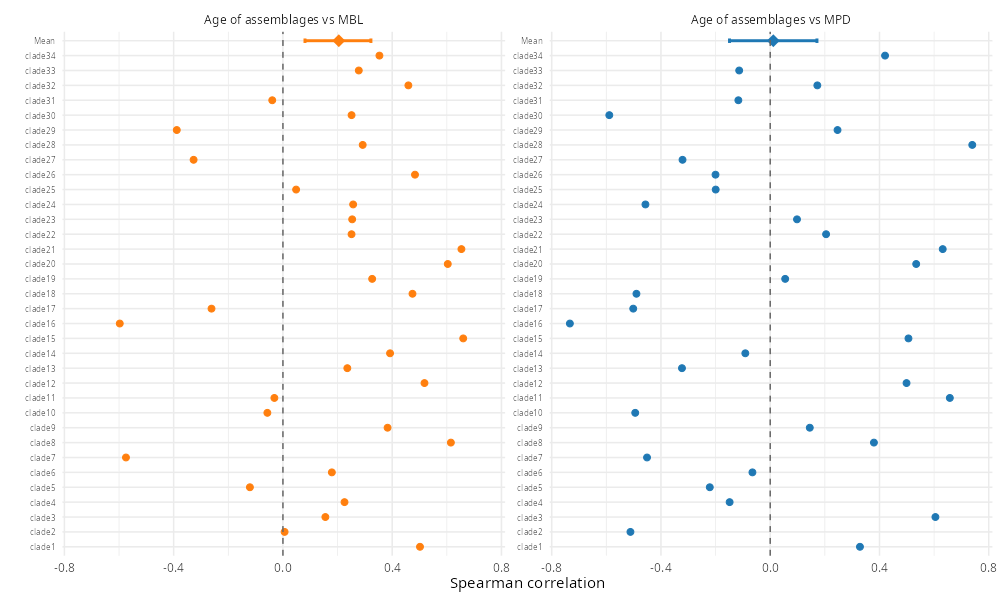


**Fig AM. Spearman correlations between assemblage age and phylometrics across amphibian clades.** Points represent clade-specific correlations between assemblage age (defined as the mean arrival time of species within assemblages) and two phylogenetic metrics: mean pairwise phylogenetic distance (MPD) and maximum branch length (MBL). The dashed vertical line indicates zero correlation. Diamonds at the top of each panel show the mean correlation across clades for each metric, with horizontal error bars denoting 95% confidence intervals calculated using Fisher’s z-transformation. The data underlying this figure can be found in <https://doi.org/10.5281/zenodo.14008084>


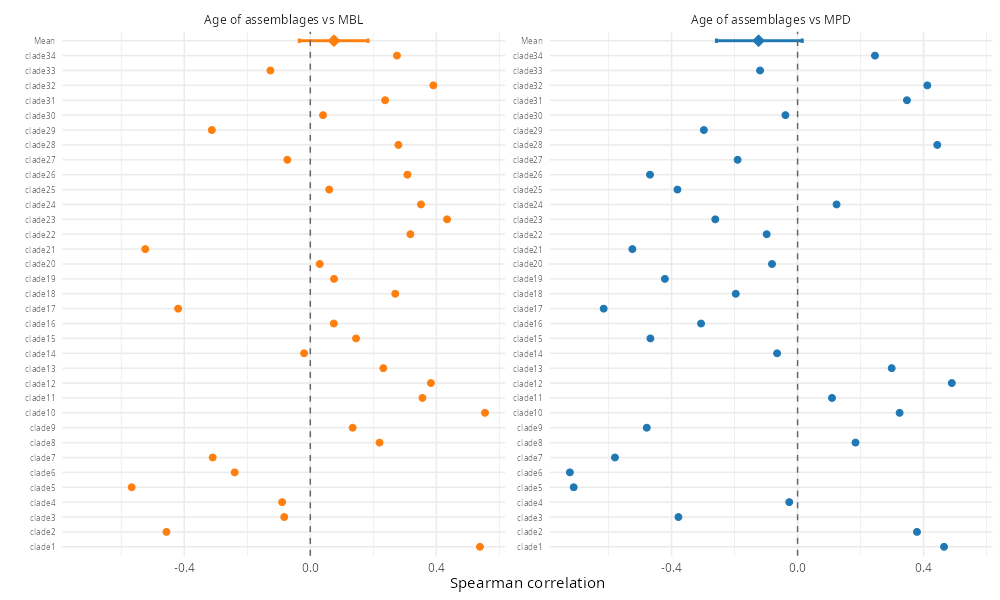


**Fig AN.** **Spearman correlations between assemblage age and phylometrics across reptile clades.** Points represent clade-specific correlations between assemblage age (defined as the mean arrival time of species within assemblages) and two phylogenetic metrics: mean pairwise phylogenetic distance (MPD) and maximum branch length (MBL). The dashed vertical line indicates zero correlation. Diamonds at the top of each panel show the mean correlation across clades for each metric, with horizontal error bars denoting 95% confidence intervals calculated using Fisher’s z-transformation. The data underlying this figure can be found in <https://doi.org/10.5281/zenodo.14008084>


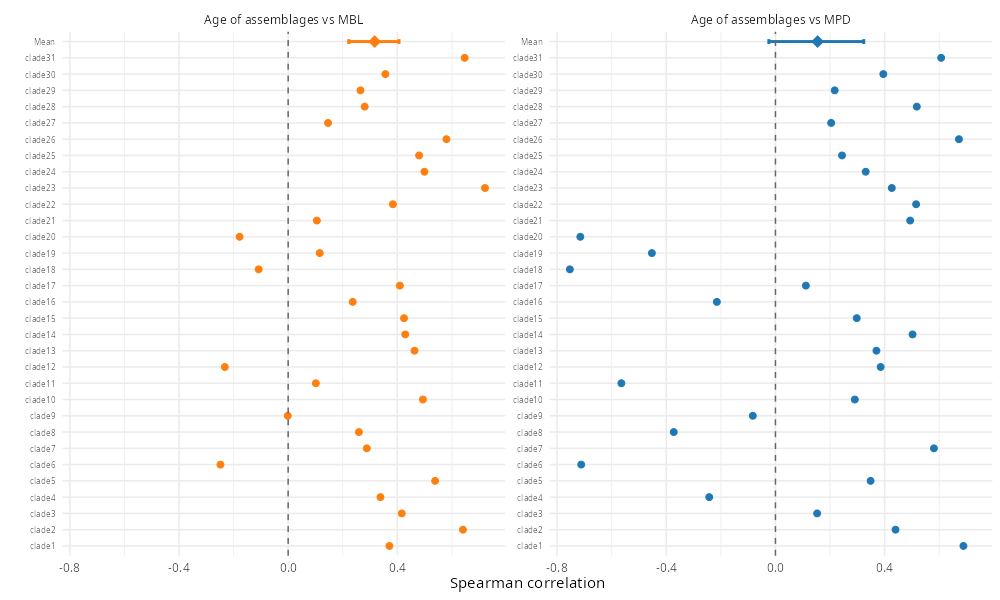


**Fig AO.** **Spearman correlations between assemblage age and phylometrics across bird clades.** Points represent clade-specific correlations between assemblage age (defined as the mean arrival time of species within assemblages) and two phylogenetic metrics: mean pairwise phylogenetic distance (MPD) and maximum branch length (MBL). The dashed vertical line indicates zero correlation. Diamonds at the top of each panel show the mean correlation across clades for each metric, with horizontal error bars denoting 95% confidence intervals calculated using Fisher’s z-transformation. The data underlying this figure can be found in <https://doi.org/10.5281/zenodo.14008084>


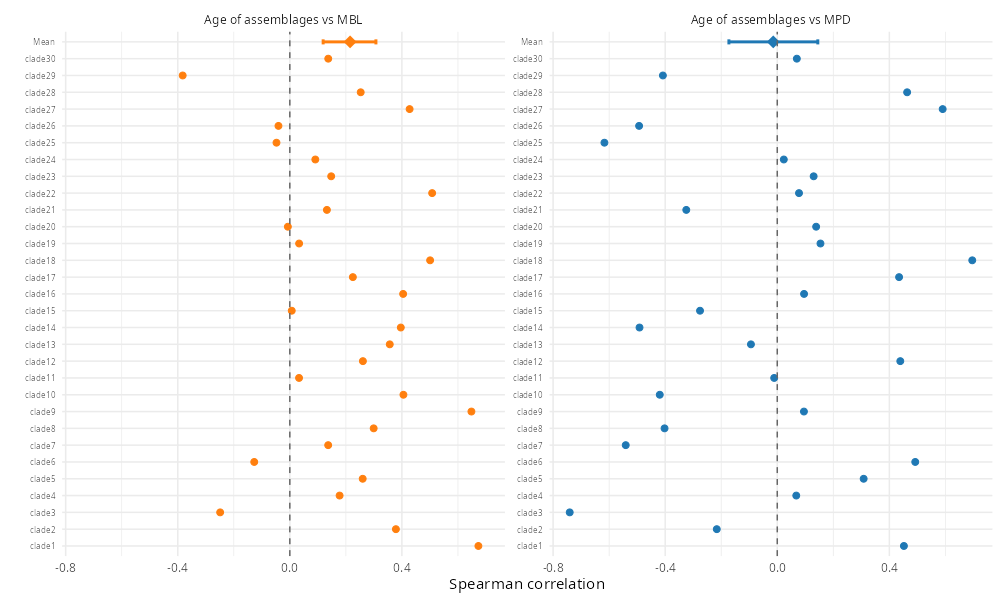


**Fig AP.** **Spearman correlations between assemblage age and phylometrics across mammal clades.** Points represent clade-specific correlations between assemblage age (defined as the mean arrival time of species within assemblages) and two phylogenetic metrics: mean pairwise phylogenetic distance (MPD) and maximum branch length (MBL). The dashed vertical line indicates zero correlation. Diamonds at the top of each panel show the mean correlation across clades for each metric, with horizontal error bars denoting 95% confidence intervals calculated using Fisher’s z-transformation. The data underlying this figure can be found in <https://doi.org/10.5281/zenodo.14008084>


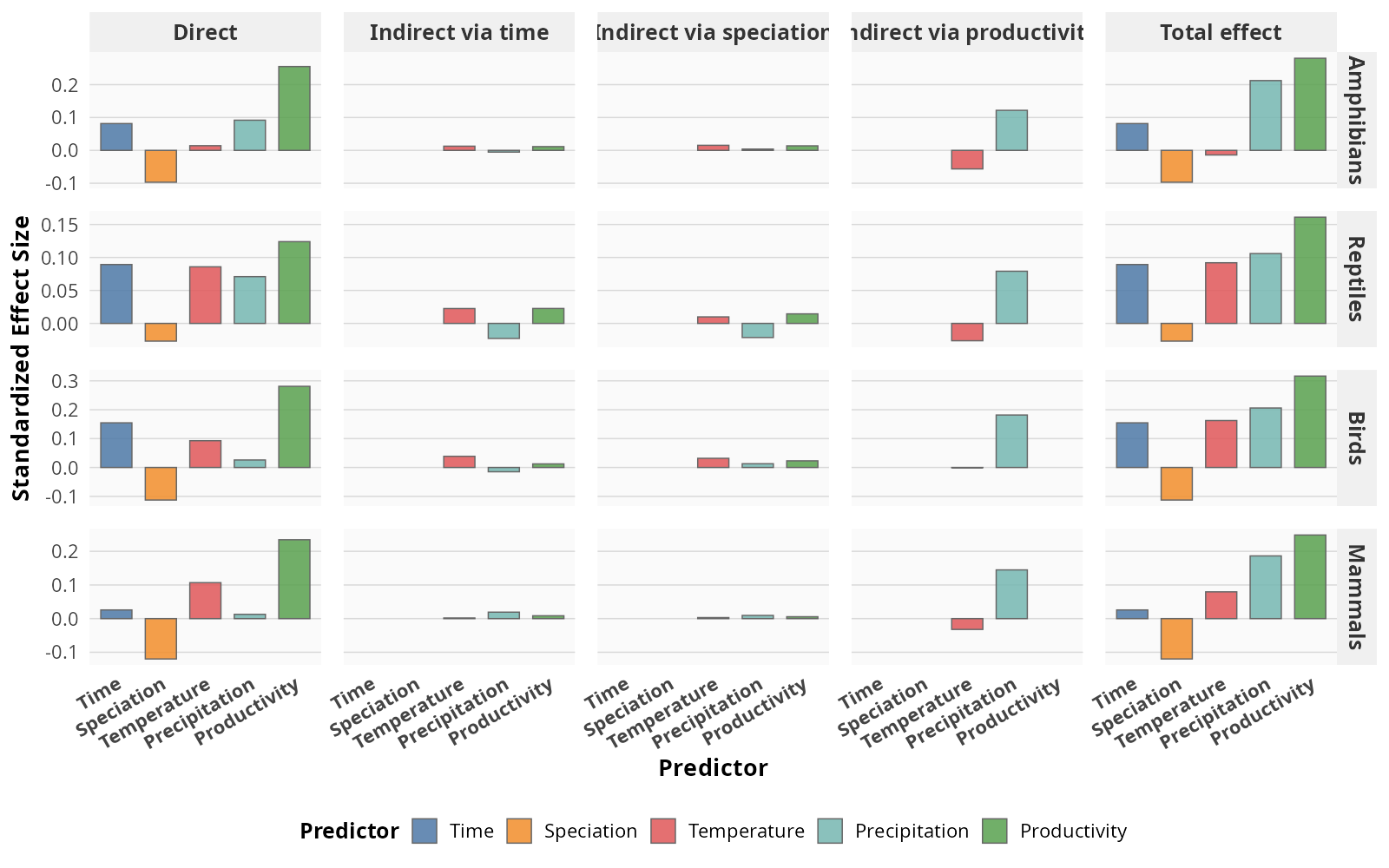


**Fig AQ. Direct, indirect, and total effects of environmental, evolutionary time, and speciation rate on species richness using MPD as a proxy for evolutionary time.** Bars show mean standardized path coefficients (β) across amphibian (34 clades), reptile (34 clades), bird (31 clades), and mammal (30 clades) clades. Evolutionary time is represented by mean pairwise phylogenetic distance (MPD), while additional predictors include speciation rate (DR estimates), temperature, precipitation, and net primary productivity (NPP). Indirect effects are grouped into three classes: environmental effects mediated via evolutionary time, environmental effects mediated via speciation rate, and climatic effects mediated via productivity. Total effects combine direct and all indirect pathways. Colours correspond to individual predictors. The data underlying this figure can be found in <https://doi.org/10.5281/zenodo.14008084>


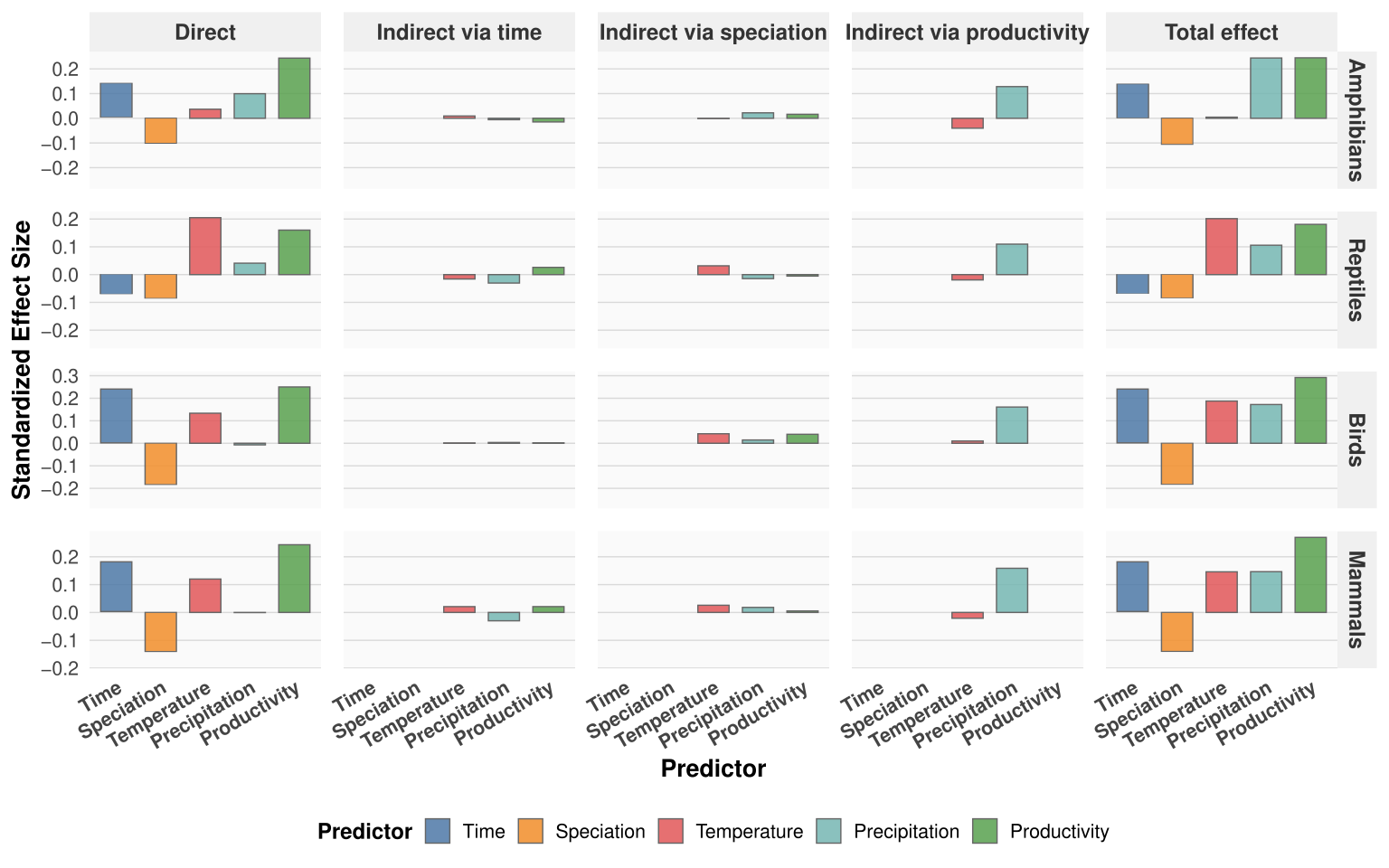


**Fig AR.** **Direct, indirect, and total effects of environmental, evolutionary time, and speciation rate on species richness using MBL as a proxy for evolutionary time.** Bars show mean standardized path coefficients (β) across amphibian (34 clades), reptile (34 clades), bird (31 clades), and mammal (30 clades) clades. Evolutionary time is represented by maximum branch length (MBL), while additional predictors include speciation rate (DR estimates), temperature, precipitation, and net primary productivity (NPP). Indirect effects are grouped into three classes: environmental effects mediated via evolutionary time, environmental effects mediated via speciation rate, and climatic effects mediated via productivity. Total effects combine direct and all indirect pathways. Colours correspond to individual predictors. The data underlying this figure can be found in <https://doi.org/10.5281/zenodo.14008084>


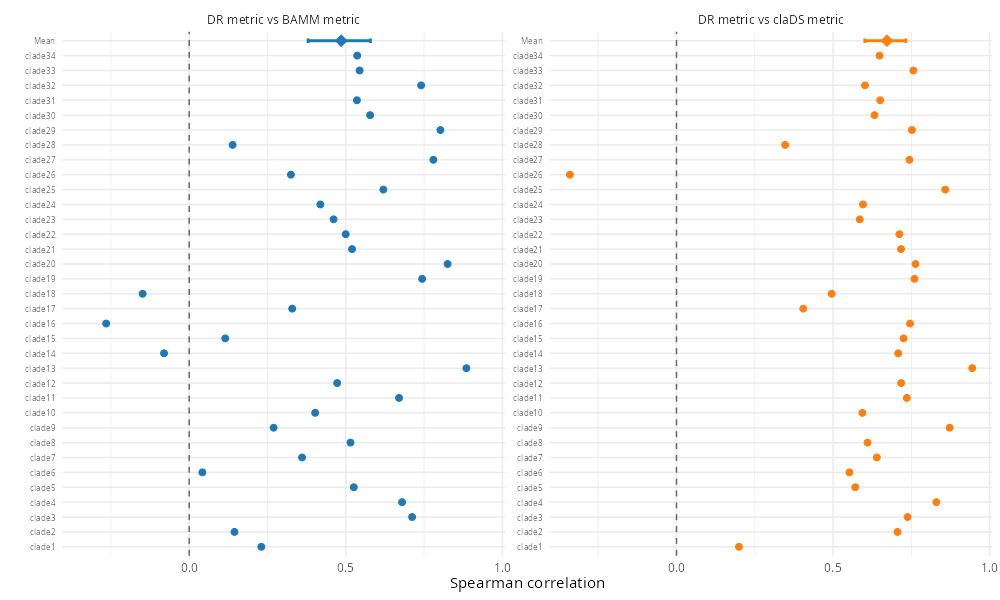


**Fig AS. Spearman correlations among speciation metrics across amphibian clades.** Points represent clade-specific correlations between the DR statistic and alternative speciation-rate estimates derived from BAMM or ClaDS. The dashed vertical line indicates zero correlation. Diamonds show mean correlations across clades with 95% confidence intervals derived using Fisher’s z-transformation. Correlations were calculated across all grid cells occupied by each clade. The data underlying this figure can be found in <https://doi.org/10.5281/zenodo.14008084>


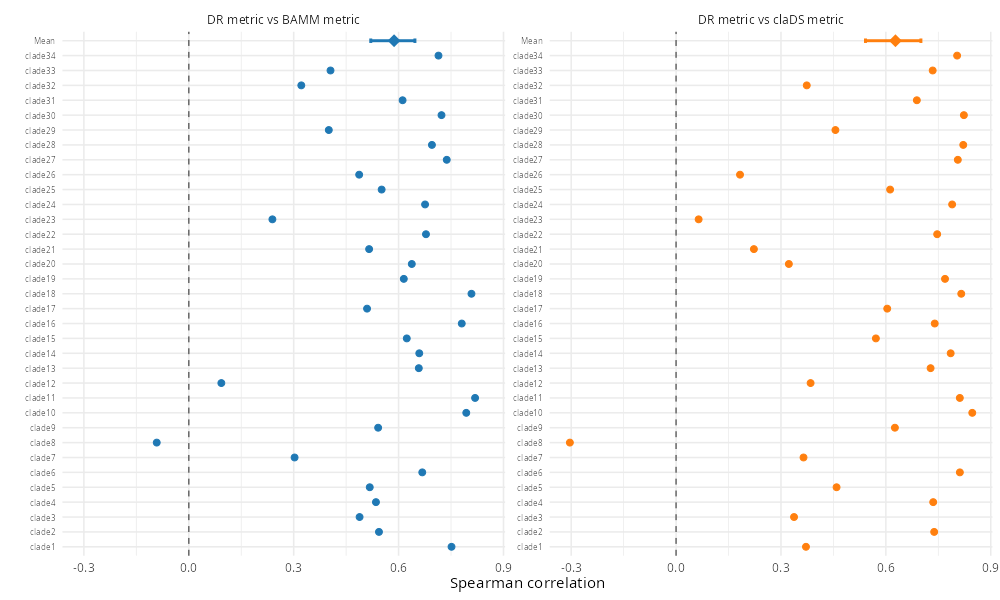


**Fig AT. Spearman correlations among speciation metrics across reptile clades.** Points represent clade-specific correlations between the DR statistic and alternative speciation-rate estimates derived from BAMM or ClaDS. The dashed vertical line indicates zero correlation. Diamonds show mean correlations across clades with 95% confidence intervals derived using Fisher’s z-transformation. Correlations were calculated across all grid cells occupied by each clade. The data underlying this figure can be found in <https://doi.org/10.5281/zenodo.14008084>


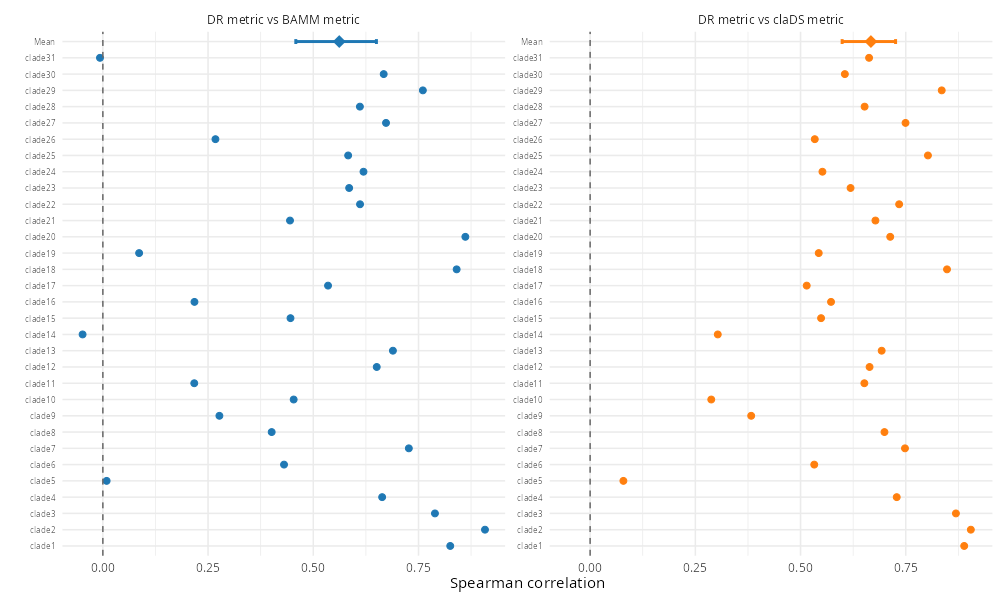


**Fig AU. Spearman correlations among speciation metrics across bird clades.** Points represent clade-specific correlations between the DR statistic and alternative speciation-rate estimates derived from BAMM or ClaDS. The dashed vertical line indicates zero correlation. Diamonds show mean correlations across clades with 95% confidence intervals derived using Fisher’s z-transformation. Correlations were calculated across all grid cells occupied by each clade. The data underlying this figure can be found in <https://doi.org/10.5281/zenodo.14008084>


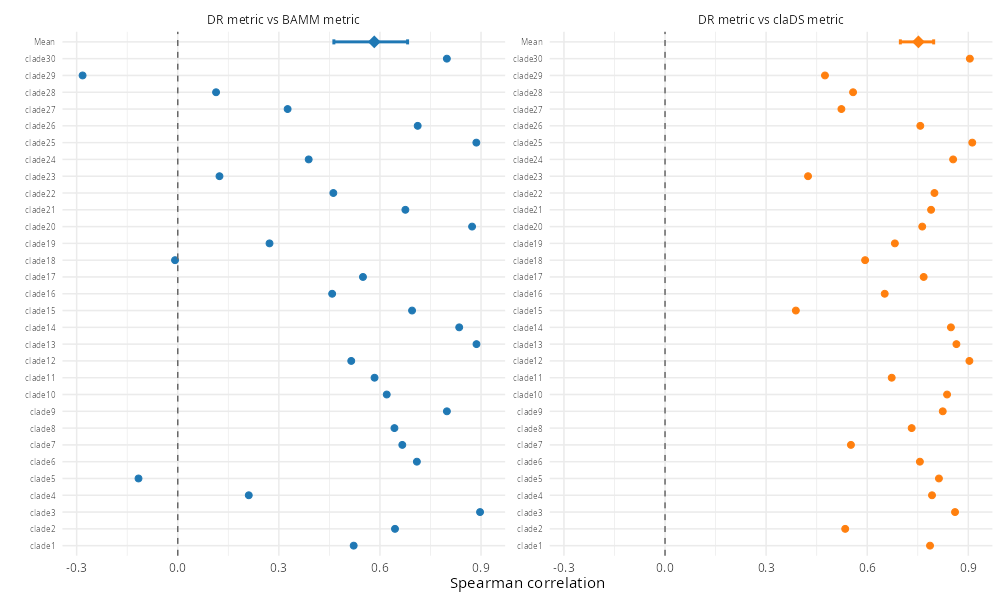


**Figure AV. Spearman correlations among speciation metrics across mammal clades.** Points represent clade-specific correlations between the DR statistic and alternative speciation-rate estimates derived from BAMM or ClaDS. The dashed vertical line indicates zero correlation. Diamonds show mean correlations across clades with 95% confidence intervals derived using Fisher’s z-transformation. Correlations were calculated across all grid cells occupied by each clade. The data underlying this figure can be found in <https://doi.org/10.5281/zenodo.14008084>


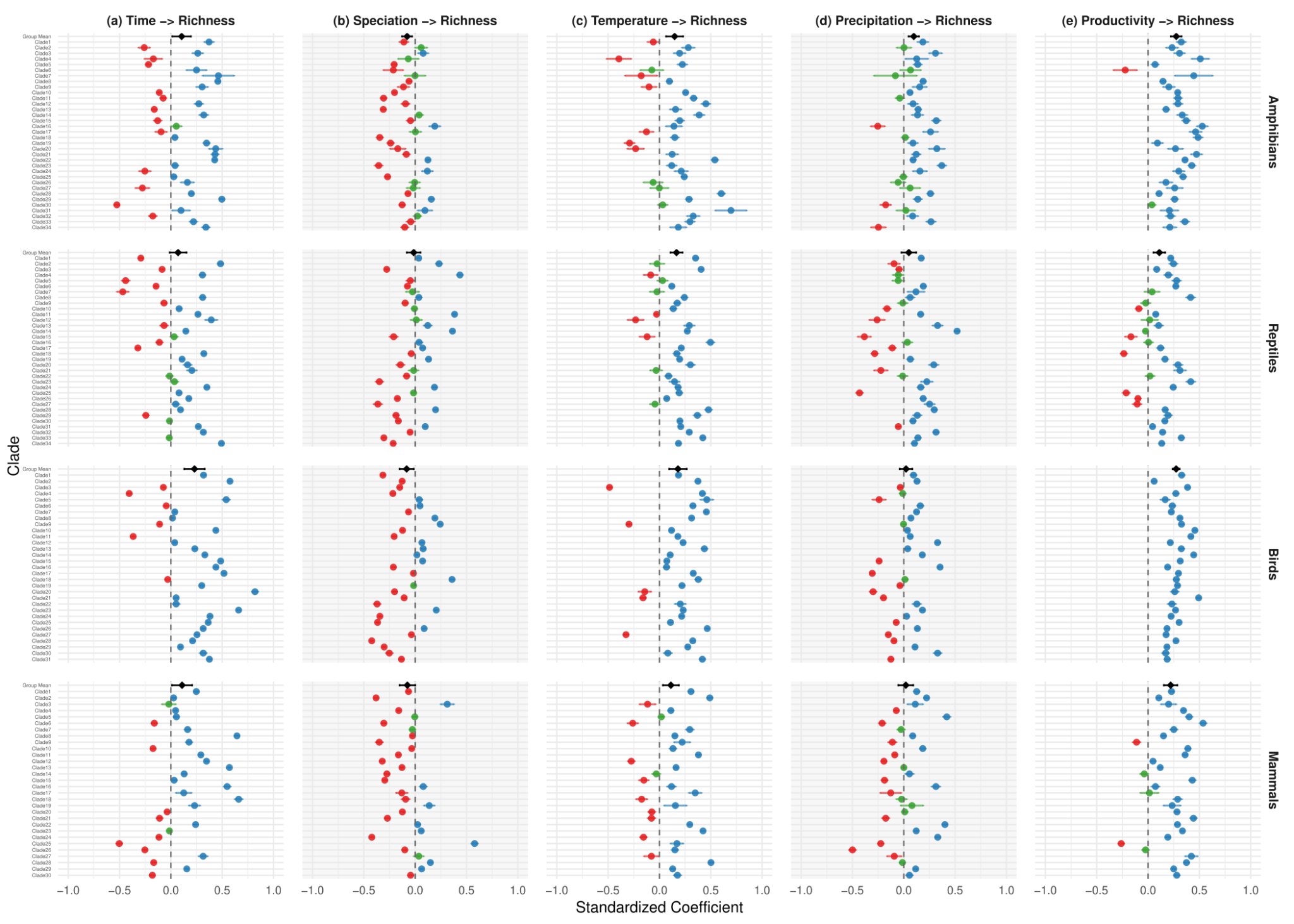


**Fig AW. Direct effects of environmental factors (temperature, precipitation, and productivity), evolutionary time (assemblage age), and speciation rate on species richness after accounting for spatial structure.** Path models include latitude and longitude as covariates to control for spatial autocorrelation. Points represent mean standardized path coefficients (β) across clades, with error bars indicating 95% confidence intervals, shown separately for amphibians, reptiles, birds, and mammals. Black diamonds indicate the mean effect size of each predictor across clades within each tetrapod class. Colours denote effect direction and statistical significance: blue, positive and significant; red, negative and significant; green, non-significant (confidence intervals overlapping zero). The data underlying this figure can be found in <https://doi.org/10.5281/zenodo.14008084>

**Table A. Summary of taxonomic coverage for the four tetrapod classes included in the study.** For each group, the table reports the total number of described species and the number of species retained in the analyses, defined by the intersection between available phylogenetic data and geographic range maps.

| Class | Described species | Sampled richness |
| --- | --- | --- |
| Amphibia | 8,747^a^ | 6,361 |
| Squamata | 11,769^b^ | 8,819 |
| Testudines and Crocodiles | 392^b^ | 310 |
| Aves | 11,032^c^ | 9,234 |
| Mammalia | 6,611^d^ | 5,145 |

^a^Amphibian Species of the World (v.6.2; <https://amphibiansoftheworld.amnh.org/>)

^b^The Reptile Database (<https://reptile-database.reptarium.cz/>)

^c^International Ornithological Committee (IOC World Bird List, v.14.1; <https://www.worldbirdnames.org/new/>)

^d^Mammal Diversity Database of the American Society of Mammalogists (v1.12.1; <https://www.mammaldiversity.org/>)

**Table B. Prior settings used in Bayesian Analyses of Macroevolutionary Mixtures (BAMM) for each tetrapod phylogeny.** Priors were specified using the setBAMMpriors function in the BAMMtools package, with incomplete taxon sampling explicitly accounted for in all analyses.

| Class | expectedNumberOfShifts | lambdaInitPrior | lambdaShiftPrior | muInitPrior | globalSamplingFraction |
| --- | --- | --- | --- | --- | --- |
| Amphibia | 1.0 | 8.4231 | 0.0033 | 8.4231 | 0.73 |
| Reptilia: Squamata | 1.0 | 5.8300 | 0.0047 | 5.8300 | 0.80 |
| Reptilia: Testudines and Crocodiles | 1.0 | 10.5472 | 0.0043 | 10.5472 | 0.83 |
| Aves | 1.0 | 2.8470 | 0.0095 | 2.8470 | 0.88 |
| Mammalia | 1.0 | 5.0619 | 0.0057 | 5.0619 | 0.78 |
