# Supplementary material for: Diversity patterns in terrestrial tetrapod clades are governed by equilibrium dynamics
Source: PLoS Biol. 2026 Apr 13;24(4):e3003730. doi: 10.1371/journal.pbio.3003730 (PMC13075687; doi:10.1371/journal.pbio.3003730)
Supplement: Attachment — Submitted filename: Response_to_Reviewers.doc [file pbio.3003730.s004.doc]

# Dear Editor and Reviewers,

# We are very grateful for your thoughtful and constructive feedback, which helped us improve the clarity and rigor of our manuscript. In response to the reviewers’ comments, we implemented several major revisions, summarized below:

# **(1) Robustness to phylogenetic structure and taxonomic imputation.** To address concerns about paraphyletic clades and imputed species, we repeated all analyses using phylogenies restricted to sampled taxa with molecular data only. These results are presented in the Supplementary Material (Figures S34–S38) and closely match those obtained from the full phylogenies, demonstrating that our conclusions are robust to imputation and tree-topology artifacts.

# **(2) Refinement of predictors and treatment of spatial structure.** We removed variables with weaker theoretical or empirical support (e.g., climate origin and centroid displacement) and clarified the interpretation of assemblage age as a model-derived hypothesis rather than a direct historical observation (Methods; Supplementary Figure S3). Because spatial autocorrelation is inherent to global biodiversity data, we quantified spatial structure in model residuals (Figure S8) and conducted sensitivity analyses including latitude and longitude as covariates (Figure S49), which confirmed that our main results are not driven by spatial bias.

# **(3) Expanded and clearer structural equation modeling framework.** We revised the SEM approach by fitting four scenario-specific models per clade, corresponding to alternative richness-generating hypotheses, and formally comparing them using AIC and BIC (Figure 4; Figures S19–S22). We also report standard model fit indices (CFI, RMSEA, SRMR; Figures S24–S27) to provide transparent diagnostics of model adequacy, while maintaining a focus on biologically interpretable pathways.

# **(4) Improved clarity, terminology, and interpretation.** We standardized terminology throughout the manuscript, revised figure captions and table legends for clarity, and added supplementary maps and schematic figures to improve visualization of key concepts (e.g., Figures S3). The Results and Discussion were refined to emphasize that net primary productivity emerges as the most consistent driver of species richness, whereas evolutionary time and speciation rates show weaker and context-dependent effects. We also adjusted the tone throughout to better distinguish inference from causation and to explicitly acknowledge modeling assumptions and limitations.

# We believe these revisions substantially strengthen the manuscript and address the reviewers’ concerns. Main changes are highlighted in blue in the revised manuscript, and references to lines are provided where relevant. We have also slightly revised the manuscript title to improve clarity and precision, changing it from “Equilibrium dynamics shape diversity patterns across terrestrial tetrapod clades” to “Equilibrium dynamics governs diversity patterns in terrestrial tetrapod clades.” We thank the reviewers again for their insightful feedback and hope that the revised manuscript is now suitable for publication in PLOS Biology.

# **Point-by-point response to reviewer comments**

# **Reviewer #1**

I've read and reviewed the manuscript entitled "Equilibrium dynamics shape diversity patterns across terrestrial tetrapod clades" submitted as a research article for PLoS Biology. The manuscript presents a conceptual framework for testing the role of dynamic equilibria in governing species richness patterns in tetrapods. The paper's strengths lies in providing a thorough test of the role of environment, time, and rates, in a single conceptual framework, across a large number of clades. The paper is very well written and the figures are of the highest quality. The subject matter is of a broad interest to biologists and therefore is, in principle, suitable for publication in PLoS Biology. I do have quite a few large concerns about the data and analyses that I would hope to see addressed, I don't think any of these would be prohibitive to the authors, but greater consideration of what information is contained in a series of phylogenetic trees based on large amounts of imputed data, should be explicitly considered and accounted for. Overall, I congratulate the authors on a commendable effort here.

**Response:** We thank the reviewer for the thoughtful evaluation and constructive comments. We appreciate the recognition of the manuscript’s conceptual framework, the scope of our analyses, and the quality of our figures. We have carefully considered all points raised and have addressed each one in detail below.

### **Comment 1.1:** I think the idea of including paraphyletic clades would make many people uneasy for this kind of analysis, and I don't think the argument pointing towards clades with extinct lineages really gets at the reason why. Lineages in the clade are clumped because of an abstract diversification modality rather than any biologically meaningful criteria (e.g., common traits or biogeography). What does it really mean to share a diversification modality and how distinct are they that they form distinguishable units? Would it be possible to just split each paraphyletic clade into a set of monophyletic clades, each sharing both a modality and common ancestry?

**Response:** We appreciate the reviewer’s concern regarding the inclusion of paraphyletic clades and the biological meaning of diversification modalities. Our rationale for defining clades based on diversification modality is to ensure that the analytical units are homogeneous with respect to the processes shaping their species richness patterns. Because the central aim of the study is to evaluate alternative richness-generating mechanisms, grouping species that share similar diversification dynamics reduces the risk of combining subunits governed by fundamentally different processes within a single clade.

We agree that diversification modalities are not defined by shared traits or biogeographic histories in the traditional taxonomic sense. Rather, they represent statistically identifiable regions of the phylogeny characterized by similar diversification dynamics. In this context, “sharing a modality” means that lineages exhibit comparable diversification behavior, which is the property directly relevant to our hypotheses. Importantly, the resulting clades are not arbitrary: modalities are objectively identified using the Laplacian spectral approach and are sufficiently distinct to form reproducible units across analyses.

Although paraphyletic clades may appear suboptimal, they are not fundamentally different, for the purposes of our analyses, from monophyletic clades in which some descendant lineages have gone extinct. In both cases, the retained clade represents a subset of lineages sharing similar diversification dynamics, while excluding others that differ in the processes of interest. From this perspective, excluding subclades with contrasting diversification regimes is preferable to enforcing strict monophyly at the cost of mixing heterogeneous dynamics within a single analytical unit.

Paraphyletic clades represent a small fraction of the dataset (approximately 11%), and their exclusion does not alter our main conclusions (Figure S34; lines 535–538). We now explicitly acknowledge this limitation in the Discussion (lines 679–697). In addition, we performed sensitivity analyses using phylogenies based on species having only molecular data, which produced highly consistent results (Figure S35–S38; lines 538–541), further indicating that our findings are robust to both paraphyly and potential artifacts of tree topology.

**Comment 1.2:** Another possible issue with the Laplacian spectrum idea (which is very interesting by the way) is that many of the mega-trees used in this study contain a large number of species whose phylogenetic position are imputed. Therefore when producing an MCC tree from a posterior, you will find these species placed towards the "average" position of the taxonomic constraint they were imputed with - e.g., forming polytomies at the crown node of the genus or family (I know this because I've done it before). I worry that this will bias the spectrum as clades with high rates of imputation (and therefore high rates of polytomies / multiple species clustering around certain nodes) might fall within a similar modality. Or, the the modalities simply don't reflect true diversification scenarios.

**Response:** We thank the reviewer for raising this important concern. We agree that phylogenetic imputation can influence tree topology, particularly by placing unsampled taxa at average positions within taxonomic constraints, potentially leading to local clustering or pseudo-polytomies in MCC trees. Such artifacts could, in principle, affect the Laplacian spectrum and bias the identification of modalities if clades with high levels of imputation were systematically grouped together.

To explicitly evaluate this possibility, we repeated the entire analytical pipeline using phylogenies based exclusively on species with molecular data. This included (i) clade delimitation based on the Laplacian spectrum, (ii) ancestral reconstructions, and (iii) all downstream path-model analyses. Importantly, the results were qualitatively unchanged. The relative importance of speciation rates, evolutionary time, and environmental drivers remained stable across analyses (Figure S35–S38; lines 257–266 & lines 538–541).

This robustness suggests that our results are not driven by imputation-induced topological artifacts, and that the Laplacian spectrum captures deeper structural properties of diversification modalities rather than superficial clustering caused by imputed placements. We now explicitly report this sensitivity analysis and discuss the implications and remaining limitations of phylogenetic imputation in the main text (Figure S35–S38; lines 257–266 & lines 538–541).

**Comment 1.3:** What this is really getting at, is that I think the method you've selected is too sophisticated for the data you have, which is largely imputed (especially for groups like amphibians). Best practice would be to find a way of clustering species into monophyletic clades based on some average modality membership across multiple phylogenies, or (more work) repeat downstream analyses across different trees from the posterior. Estimates of speciation rates and other metrics should be doing this in any case (i.e. not using MCC tree, but averaging values across posterior).

**Response:** We agree that extensive phylogenetic imputation, particularly in groups such as amphibians, raises legitimate concerns about the reliability of fine-scale inference. To directly address this concern, we conducted comprehensive sensitivity analyses using phylogenies restricted only to taxa with molecular data, thereby eliminating all imputed placements. Importantly, this was not limited to a subset of downstream steps (see previous reply). Across tetrapod groups, the results obtained from trees based on molecular data were qualitatively indistinguishable from those based on the full phylogenies (Figure S35–S38).

These results indicate that our conclusions are not contingent on the use of imputed phylogenetic placements. Instead, the Laplacian spectrum appears to capture broad, repeatable features of diversification structure that are robust to reasonable variation in topology and taxon sampling. While we agree that averaging diversification metrics across posterior samples is best practice in principle, we note that our key inferences rely on relative comparisons across many independent clades, rather than on precise estimates of deep-time diversification parameters for any single lineage. We also emphasize an important trade-off inherent in the reviewer’s suggestion. Restricting analyses to trees with molecular data reduces potential bias from imputation, but at the cost of substantial taxonomic incompleteness, which can itself distort diversification patterns and spatial richness gradients.

By showing concordant results across both full and genetic-only phylogenies, we demonstrate that our framework is robust to this trade-off. We now explicitly acknowledge in the manuscript that phylogenetic uncertainty is uneven across tetrapod groups. However, the strong agreement across sensitivity analyses gives us confidence that the central conclusions of the study are not driven by phylogenetic imputation or by overfitting complex methods to noisy data. These points are now clarified in the main text (lines 257–266 & lines 538–541).

**Comment 1.4:** The authors use speciation rates because they are more reliably estimated than extinction rates, citing Louca and Pennell etc. However, speciation rates are non-identifiable with extinction rates - meaning both rates can't be estimated from the infinite possible combinations that give the same net-diversification rate. This isn't a problem restricted to extinction rates. I would encourage the authors to use net diversification rates instead (this is what ClaDS and BAMM give you anyway for tip rates, I thought). I'm fine with the authors use the DR metric and stating it is known to be more strongly correlated with recent speciation rates in Title's study but they should use clear language regarding identifiability problems.

Response: Thank you for your point. We have revised this section to more clearly articulate the identifiability issues associated with estimating diversification dynamics from molecular phylogenies. As the reviewer notes, speciation and extinction rates are mathematically non-identifiable from extant-only phylogenies, and this non-identifiability propagates directly to net diversification rates as well. Importantly, as highlighted by Louca & Pennell (2020), these concerns are most pronounced for rates through time, whereas present-day (tip) speciation rates can be meaningfully estimated. As Louca & Pennell (2020) emphasize in the Conclusion section, *“Without further information or biologically well-justified constraints, in general extant timetrees alone cannot be used to reliably infer speciation rates (except for the present day), extinction rates, or net diversification rates.”*

Although methods such as BAMM or ClaDS provide “diversification” or “tip rates” as an output, these values are generally interpreted as reflecting variation in recent speciation rates rather than true net diversification, because extinction remains largely unidentifiable. The DR statistic similarly correlates most strongly with recent speciation rates (Title & Rabosky 2019 - Methods Ecol. Evol.). Importantly, even though deeper-time estimates remain uncertain, some recent studies demonstrate that speciation rates near the tips of the phylogeny can be reliably inferred (Kopperud et al., 2023 - PNAS; Vasconcellos et al., 2022 - Evolution).

Together, these studies support the view that tip-based metrics, whether from DR, BAMM, or ClaDS, are best interpreted as estimates of recent speciation rates. In line with this emerging consensus, we therefore frame our analyses explicitly in terms of speciation rather than net diversification, as the latter would imply a level of parameter identifiability not supported by current theory. These clarifications have now been incorporated into the main text (see lines 359-379).

**Comment 1.5:** Why is the grid cell the appropriate scale to test these hypotheses? They are not independent units of analysis because species contribute to multiple grid cells, and spatial variables are autocorrelated. How have the authors addressed these issues? Do the SEMs account for autocorrelation?

**Response:** Grid cells are a widely accepted and well-established unit of analysis in macroecology and biogeography for testing large-scale hypotheses about species richness and its drivers (Hawkins et al. 2003; Jetz et al. 2012; Saupe et al. 2019; Harvey et al. 2020). The 1° × 1° resolution used here represents a common compromise between ecological relevance, spatial coverage, and the known uncertainty of vertebrate range maps, and has been shown to minimize false presences while retaining broad-scale signal in richness patterns (Hurlbert and Jetz 2007). We fully agree that grid cells are not perfect units of analysis, but any alternative choice would face similar or even more severe challenges. For example, using ecoregions would introduce issues related to their subjective delimitation, heterogeneous areas, shapes, and contiguity. Grid cells, in contrast, have standardized area and shape, which is why they are widely used in large-scale biogeographic analyses. Using larger grid cells would not eliminate the problem of spatial autocorrelation.

Path models by themselves cannot account for spatial autocorrelation, although it is possible to evaluate its presence using diagnostics such as Moran’s I calculated on path residuals. We performed this assessment and indeed found strong spatial autocorrelation in the residuals, as expected (see Fig. S8). Because there is no simple or widely accepted way to incorporate spatial autocorrelation directly into path models, we took a pragmatic approach. Specifically, we included both latitude and longitude as additional predictors in the SEMs and re-ran the path models for each clade. The main results remained qualitatively similar (particularly the strong and consistent effect of productivity on species richness), although the effect sizes of some predictors became slightly weaker or stronger in certain clades. These changes, however, did not alter our overall conclusions. For instance, Figure S15 (path models without spatial predictors) and Figure S49 (models including latitude and longitude) show that the core patterns remain robust.

Throughout the revised manuscript, we now more explicitly acknowledge the presence of spatial autocorrelation in our data (lines 432-436, explain why it is challenging to address in a path-modeling framework, and clarify that our additional tests confirm that this issue does not affect the key conclusions (see lines 557-560).

**Comment 1.6:** Wouldn't geographic location / biome be more relevant that cool or warm epoch? There are cooler and warmer regions in any given epoch. This is also confounded with time because older origins = warmer, younger = cooler as seen in Figure 4. I don;t think this variable adds more information than time.

**Response:** Thank you for the suggestion. We fully agree that climate origin can be confounded with clade age, as older clades tend to originate in warmer periods and younger clades in cooler periods. Following your recommendation, we have removed climate origin from our analyses (see Comment 1.8 for more details).

**Comment 1.7:** Ancestral areas aren't really estimated because there is no information on the paleolocation of the areas themselves. This would be impossible to define in the first place because the areas aren't defined by anything that can be projected through time (e.g., climate), they are just contemporary representations of relatedness in space.

**Response:** We acknowledge that ancestral range reconstructions cannot perfectly capture the absolute geographic positions of historical areas, given the changes in paleoclimate and continental configurations through time. However, the primary goal of our approach was not to provide geographically exact ancestral locations, but rather to quantify the relative timing of species arrivals into assemblages. In other words, we aim to determine whether species-rich areas tend to have lineages that have been present longer than in other regions (regardless of their exact past locations). While traditional phylometrics such as MPD or MBL are often used as proxies for assemblage age, they can be confounded with species richness and diversification rates and do not incorporate information on lineage movements. In contrast, our historical biogeography-based metric, despite its limitations, provides a mechanistically informed estimate of evolutionary time within assemblages, capturing temporal patterns of colonization that MPD and MBL cannot. We therefore consider it a suitable and robust measure for linking evolutionary history to species richness. Additionally, we have now downplayed some aspects of the ancestral reconstructions in the Methods section (see lines 291-298 & lines 328-336), and we believe these clarifications make the limitations more appropriately acknowledged than before

**Comment 1.8:** While nice ideas in principle, I would consider removing the centroid displacement / rate / climate origin because I don't trust that they represent what you claim they represent.

**Response:** We agree that centroid displacement and its rate were not essential for our study and did not provide additional insight. Therefore, alongside climate origin, we have removed centroid displacement and its rate from the analyses. Consequently, we revised the section describing clade traits to focus on the remaining, well-characterized traits (see lines 438–456), and updated all relevant sections in the main text, including the Results (lines 515-530) and Discussion (lines 625–639), to reflect these changes.

**Comment 1.9:** Further, linear models don't account for non-independence of clade's traits/geography/speciation rates - this should be tested with a pgls or similar model but paraphyletic clades mean this would be difficult to implement.

**Response:** We thank the reviewer for raising the important issue. In our study, the analytical units are non-overlapping taxonomic clades identified using a Laplacian spectrum approach, each representing a distinct evolutionary lineage with its own internally estimated relationships among richness-generating processes. These clades span different taxonomic ranks, vary widely in age, and include a small number of paraphyletic groups. As a result, they cannot be represented as terminal tips of a single, coherent phylogeny in a way that would allow construction of a biologically meaningful phylogenetic variance–covariance matrix required for PGLS or related approaches.

More importantly, implementing a clade-level PGLS would require merging independently inferred phylogenies for birds, mammals, reptiles, and amphibians into a single tetrapod-level backbone. Although the topology of such a backbone is well established, the underlying class-specific phylogenies were reconstructed using different data sources, calibration strategies, clock models, and inference frameworks. Consequently, branch lengths are not guaranteed to be directly comparable across classes or to lie on a shared evolutionary scale. Because PGLS explicitly relies on branch lengths to model expected covariance among units (Martins and Hansen 1997; Freckleton et al. 2002), combining such phylogenies could introduce artefactual patterns of phylogenetic dependence unrelated to biological processes.

In addition, our objective is not to analyse trait evolution within a shared phylogeny, but to compare emergent clade-level properties, specifically, the strength of historical, diversification, and environmental drivers of species richness, across distinct evolutionary lineages. When clades are non-overlapping and capture independent evolutionary histories, treating them as independent analytical units is a standard and well-established practice in macroecology and macroevolution (Ricklefs 2004, 2007; Jablonski 2007; Rabosky 2009). This cross-clade comparative framework has been widely used to investigate how diversification dynamics, ecological limits, and historical contingencies vary among major lineages. We have clarified these assumptions and their implications in the revised Methods to make our comparative design explicit and transparent (see lines 446–456).

**Comment 1.10:** Table 1 elegantly sets up four alternative models for the origin of richness. I would have expected the authors to fit these four alternative SEM's and use model selection to see which model provides the best fit, rather than just throwing every path in the same model which is far more difficult to interpret. Model selection would allow the authors to tally the number of clades which support each of the four scenarios.

**Response:** This is a very neat idea, thank you! We agree that formally comparing scenario-specific SEMs provides a clear and intuitive way to evaluate support for the four richness-generating scenarios outlined in Table 1. We implemented this approach by fitting four alternative, scenario-specific path models to each clade and comparing them using both AIC and BIC. For each clade, the scenario with the lowest information criterion value was interpreted as receiving the strongest support.

We now make this procedure explicit in the Methods (lines 415-425) and Results (lines 502-510) and report the proportion of clades supporting each scenario within each tetrapod group into a new Figure in the main text (Figure 4). Detailed results are provided in the Supplementary material (Supplementary Figures S19-S22). Results based on AIC and BIC were highly concordant and show that the Stable Persistence scenario is most frequently supported across tetrapods, with varying secondary support for the Historical Legacy, Diversity Cradles, and Speciation Balance scenarios. These additions strengthen the link between Table 1 and the empirical analyses and clarify how support for alternative richness-generating scenarios was quantified.

**Comment 1.11:** Path model statistics beyond the slopes were not presented in the paper. What about chi-square p value - do the models fit?

**Response:** We agree that providing additional fit statistics can clarify model performance. In path models with large datasets, the χ² test is known to have very high power, often leading to rejection of well-fitting models due to minor deviations. Thus, significant χ² values are not unexpected in our case (Kline 2016). To provide a more robust assessment, we computed alternative fit indices, including the Comparative Fit Index (CFI), the Root Mean Square Error of Approximation (RMSEA), and the Standardized Root Mean Square Residual (SRMR) for each clade (Figures S24–S27; see Methods, lines 402–415). Across tetrapod clades, the majority of models meet or exceed commonly accepted thresholds for CFI (≥0.95) and SRMR (≤0.08), whereas RMSEA values were frequently above the typical cutoff (≤0.05), which is a known issue for models with many observations or complex structures (MacCallum et al. 1996). Importantly, all paths included in the model were theoretically justified and supported by prior studies. As detailed in the Methods (lines 402–415) and Results (lines 510–513), emphasis in macroecological and macroevolutionary path models is placed on the plausibility and support of hypothesized paths rather than strict reliance on χ² or fit indices, while ensuring that model assumptions are reasonably met.

**Comment 1.12:** Why 50 clades? Was any test done to estimate optimal number of branching modes

**Response:** The choice of 50 clades was based on a balance between capturing sufficient number of replicates and ensuring that each clade included a sufficient number of species for robust analysis. Applying the Laplacian spectrum to the full phylogeny without pre-specifying the number of clades can result in a very large number of small clades, many with few species, which would be unsuitable for our analyses focused on clade-level richness and diversity patterns. Selecting 50 clades retrieves groups spanning a broad range of ages and species numbers (see Figure S13-S14), which aligns well with the objectives of our study. To verify that this choice did not influence our conclusions, we also tested alternative settings of 40 and 60 modalities (clades). These analyses produced qualitatively similar results (Figures S32–S33, lines 253-257 & lines 535-538), confirming that the patterns reported in the manuscript are robust to the specific number of modalities used. Additionally, performing the modality search using only phylogenies based on molecular data produced highly congruent findings (see comment 1.2 for more details). These checks confirm that the patterns reported in the manuscript are robust to both the number of modalities and the source of the phylogenetic data.

**Comment 1.13:** How many species were excluded by only retaining clades of 50 species or more - would this bias against recent + slow diversifying clades?

**Response:** We thank the reviewer for raising this point. To assess whether restricting the analysis to clades with at least 50 species could bias against recent or slowly diversifying clades, we quantified the proportion of species excluded relative to the set of species for which both phylogenetic and distributional data were available. From this matched dataset, we retained approximately 97.8% of mammals (out of 5,145 species), 99.98% of birds (out of 9,234 species), 99.9% of amphibians (out of 6,361 species), and 99.6% of reptiles (out of 9,129 species). In all cases, fewer than 2.5% of species from the matched datasets were excluded by the clade-size criterion of 50 species or more.

Given this near-complete retention of species with matched phylogenetic and distributional data, the 50-species threshold is unlikely to introduce a systematic bias against recent or slowly diversifying clades. Instead, it ensures statistical robustness of clade-level estimates while maintaining broad taxonomic coverage across tetrapods. We have clarified this point in the Methods (lines 241–243).

**Comment 1.14:** Some recent criticisms about the meaning of grid-cell values of speciation rates are relevant here. Speciation happens at coarser scales than 1 degree grids.

**Response:** We agree that speciation does not occur at the spatial scale of 1° grid cells, and we do not interpret our results as implying in situ speciation within individual cells. The speciation rates used in our analyses are species-level (tip-based) estimates derived from phylogenies and subsequently mapped onto space based on present-day species distributions. Thus, grid-cell values reflect the evolutionary characteristics of the lineages occurring in an area, not the location or scale at which speciation events took place.

Our analyses therefore test whether species-rich areas tend to be occupied by lineages with higher intrinsic speciation rates, an approach that is widely used to infer the geography of diversification in macroecology and macroevolution (Jetz et al. 2012; Title and Rabosky 2019; Harvey et al. 2020). We now clarify this interpretation in the main text (lines 196-199).

**Comment 1.15:** Paraphyletic clades again raise an issue with reconstructing historical biogeography, because tools like BGB assume monophyletic clades to estimate parameters

**Response:** We agree that some biogeographic tools, such as BioGeoBEARS, assume monophyletic clades. However, only a small fraction of the clades in our dataset (11%) are paraphyletic. Importantly, we verified that excluding all paraphyletic clades and re-running our analyses produced results that were qualitatively identical to those reported in the manuscript (Figure S34).

**Comment 1.16**: Figure 1. Label panels a/b/c/d or 1/2/3/4 (whichever is journal preference); Possibly remove extinction line from Historical Legacy and Diversity Cradles (or maybe indicate that it is not always diversity dependent - could make it a dashed line?)

**Response:** Thank you very much for these suggestions. We have labeled the panels as A–D and updated the extinction lines in the Historical Legacy and Cradles of Diversity scenarios to be dashed, indicating that extinction is not always diversity-dependent and is not relevant in these scenarios. The figure caption has been revised accordingly.

**Comment 1.17:** Figure 2. Very nice figure!

**Response:** Thank you for the positive feedback.

# **Reviewer #2**

[identifies himself as Allen Hurlbert]

The authors unite four different hypotheses for diversity patterns within a single framework that considers the relative importance of equilibrial dynamics, speciation rate, and time. Through this unified framework, they are also able to evaluate the relative strength of direct and indirect paths of the relevant historical, evolutionary and environmental variables for a large number of terrestrial vertebrate clades. I would argue that this manuscript represents the best attempt to date to evaluate the relative importance of all of these hypotheses, and does so in a clear, elegant fashion. This paper is very well written, and the analyses and inferences provided here represent such an advance and clarification of a tangled literature that they are likely to end up in textbooks. Publish this.

**Response:** Thank you so much for these very encouraging comments. We’re really glad to hear that the framework and analyses come across clearly and that the contribution is seen as valuable to the field.

**Comment 2.1:** Section 2.7: In the context of traits, "size-related" can be interpreted as body size rather than clade size as you intend. Consider clarifying.

**Response:** We thank the reviewer for raising this point. We have clarified and reformulated the description of clade traits in the Methods (see lines 438–456), explicitly distinguishing clade diversity (number of species) and geographic extent (range size) from physiological and spatial–historical traits. This avoids any confusion with individual body size, which is not considered in these analyses. Terminology throughout the manuscript has been standardized to reflect this distinction, ensuring that “size-related” clearly refers to clade-level diversity and extent rather than individual species traits.

# **Reviewer #3**

The manuscript presents an innovative conceptual framework for distinguishing the mechanisms that generate species richness and applies it to a global analysis of tetrapods. Using a path modelling approach and clades defined by spectral partitioning, they simultaneously evaluate the role of evolutionary time, diversification rates, and environmental factors (productivity, temperature, precipitation). The results highlight a predominant role for productivity, exerting a dominant and direct effect on richness, while time and temperature play more contextual roles and speciation rates contribute little, reflecting mostly turnover dynamics. The work aims to reconcile classical explanations and provide an integrative framework for future studies.

**Response:** We thank the reviewer for the positive and thoughtful summary of our work.

**Comment 3.1:** The manuscript is very well written and clearly reflects a large amount of work. However, in my view the authors make some very strong claims given the limitations of the available data. Much of the analysis relies on adjusting models (path equation model) to data created with their own inferences, on top of other models (e.g. phylogenies), which are then used again to interpret and explain the very patterns from which they were initially derived. This circularity makes it difficult to fully evaluate the robustness of the conclusions, and the underlying processes are not always clearly distinguished from the assumptions required to model them.

**Response:** We thank the reviewer for the comment. We agree that the analytical design is complex. However, this complexity is intentional and reflects the need to robustly infer the processes underlying observed species richness patterns. While all datasets have inherent limitations, global tetrapod data represent the best currently available taxonomic, geographic, and phylogenetic coverage. To anticipate potential biases and strengthen inference, we employed multiple macroevolutionary approaches (e.g., BAMM, DR, and ClaDS) and moved beyond simple phylometrics as proxies for evolutionary time by incorporating ancestral state reconstructions. These choices naturally introduce complexity, but they are essential for capturing the multidimensional nature of clade-level diversification and environmental effects. Importantly, our analyses rely on independent clade-level traits rather than recycling the same data to explain themselves, and sensitivity analyses confirm that our main conclusions are robust across alternative modeling choices and phylogenetic subsets (see Robustness analyses section in the Results, lines 532-560). Without specific examples of where the circularity could be, it is difficult to address concerns about circularity further. We debated some of the potential circularities in the Discussion (lines 731-749) but we are confident that our approach provides a robust framework for assessing the relative contributions of productivity, evolutionary time, and speciation rates to richness patterns.

**Comment 3.2:** The study demonstrates impressive technical and computational capacity, but at times it feels somewhat disconnected from empirical grounding. For example, the biogeographical reconstructions are themselves estimates based on multiple assumptions, and the evolutionary variables are also estimates; building further interpretations on top of these layers of uncertainty leads to claims that may be overstated.

**Response:** We fully agree that both biogeographic reconstructions and evolutionary rate estimates carry uncertainty, as they necessarily rely on model-based inference. To address this, we deliberately employed state-of-the-art methods, explored alternative macroevolutionary estimators (e.g., BAMM, DR, ClaDS), and incorporated ancestral state reconstructions to reduce reliance on single proxies. We also conducted extensive sensitivity analyses to evaluate the robustness of our conclusions across phylogenetic subsets, alternative model specifications, and different clade definitions. These steps were designed precisely to mitigate the risks associated with stacking multiple layers of inference.

In response to the reviewer’s concern, we have carefully re-examined the manuscript to ensure that all claims remain appropriately calibrated to the uncertainties inherent in the data and methods. Where needed, we have softened wording and clarified limitations to avoid any perception of overstating results (e.g., lines 328-336). While uncertainties remain, inevitably, in global-scale comparative analyses, we are confident that the main conclusions are supported consistently across methodological choices.

**Comment 3.3:** One methodological novelty is the selection of clades from phylogenies and the repeated analyses across clades to increase replication. This is indeed a strength of the approach, but it should be made clearer how this differs from previous work and how it affects the interpretation of the results.

**Response:** We thank the reviewer for highlighting this point. We agree that the clade-selection procedure represents an important methodological contribution, and we have now clarified its novelty and implications more explicitly in the manuscript. Previous studies assessing the drivers of species richness have typically relied on either (i) analyses conducted at a single global scale, or (ii) comparisons among subjectively defined higher taxa (e.g., families, orders) or nested clades. In contrast, our approach introduces an objective, data-driven procedure to delimit clades based on homogeneity in diversification dynamics, using spectral partitioning of phylogenetic trees. This ensures that each clade represents a coherent evolutionary unit with internally consistent diversification processes, which is important for unbiased estimation of the effects of evolutionary time, speciation rate, and environmental variables.

Importantly, the replication across dozens of independently defined clades allows us to assess the generality of mechanisms rather than relying on a single phylogeny-wide pattern. We have added it in the Introduction (lines 150-154), revised the Methods (see lines 244-252) and Discussion (see lines 711-730) sections to better highlight how this clade-based replication, combined with process-homogeneous subtrees, enables more robust inference and provides stronger tests of competing explanations for species richness patterns than previous comparative work.

**Comment 3.4:** Add "1." before "Introduction", because the other paragraphs are numbered and the introduction is not (unify it).

**Response:** Thank you for noticing this. The numbering of the Introduction (and in the other sections) has been removed, as the journal requirements do not include numbering.

**Comment 3.5:** Unify the format of the references: remove the "," before the year in the following references: Yu & Wiens, 2024; Allen et al., 2006; Storch & Okie, 2019; Barreto et al., 2021.

**Response:** Thank you! The formatting of these references has been corrected.

**Comment 3.6:** In the sentence "Although these explanations frequently operate together rather than as mutually exclusive alternatives (Cerezer et al. 2022; Machac 2020; Pontarp & Wiens 2017), their conceptual boundaries remain debated (Pontarp et al. 2019), highlighting the need to move beyond the traditional trichotomy." I suggest adding some classic references to provide a broader historical perspective, such as Pianka (1966, 1989), Fischer (1960), and Rosenzweig (1995).

**Response:** We thank the reviewer for the suggestion. We have now reformulated the sentence and added classic references to provide a broader historical perspective (lines 54-58).

**Comment 3.7:** In "We first delineated clades objectively using the Laplacian spectrum of large-scale phylogenies", I recommend removing the word "objectively", since the method itself is not objective but rather a mathematical procedure that summarizes tree properties and allows comparisons across trees. Please also add the citation for the method (Characterizing and Comparing Phylogenies from their Laplacian Spectrum).

**Response:** Thank you for this suggestion. We have removed the word “objectively” and added the citation for the method (Lewitus & Morlon, 2016) (lines 170-173). By “objectively,” we had intended to convey exactly what the reviewer noted, a procedure based on the mathematical properties of the clades, as opposed to subjective selection based on taxonomic categories or pre-selected traits.

**Comment 3.8:** Please add in the Supplementary Material a map of this variable, as well as the evoregions from Nakamura et al., so readers can better visualize the approach.

**Response:** Thank you for the suggestion. We realized that our original description may not have been sufficiently clear about the nature of the evoregions framework. Nakamura et al. do not define a universal or fixed set of evoregions. Rather, they provide a methodological tool to delimit phylogenetically informed evolutionary regions separately for each clade. As a consequence, evoregions are clade-specific and cannot be represented by a single global map applicable across taxa.

To help readers visualize how this approach works, we have added a map to the Supplementary Material illustrating the estimation of assemblage age for an hypothetical clade, including the delimitation of its evolutionary regions and the subsequent inference of species arrival times (see Figure S3, lines 288-290). This figure is intended as a conceptual and methodological illustration of the procedure applied consistently across all clades, rather than as an exhaustive set of maps for every clade, which would be impractical given their number.

**Comment 3.9:** In the sentence "This approach explicitly estimates the timing and likely regional arrival of lineages into assemblages", I suggest replacing "explicitly estimates" with "infers" or "hypothesizes", since the method relies on multiple assumptions.

**Response:** Thank you! We have replaced "explicitly estimates" with "infers" to reflect that the method relies on assumptions rather than direct measurement (lines 291-292).

**Comment 3.10:** In reporting temporal resolution, instead of "1 × 10⁻⁵ million years", please simplify and write "10 years". Avoid overcomplicating the text unnecessarily.

**Response:** We thank the reviewer for the suggestion. The temporal resolution has been simplified to "10 years" for clarity (lines 324-326).

**Comment 3.11:** While innovative, this metric is extremely complex and based on several assumptions, including reliance on a single phylogenetic hypothesis. Past ranges are inferred only from present distributions, which is a weak proxy for reconstructing historical dynamics or constraints. The variable is presented as if it were directly encoding "history," but in fact it does not represent any historical data. What it truly captures are present-day ranges combined with environmental variables (temperature, precipitation), whereas all historical aspects are hypotheses derived from phylogenies and ancestral reconstructions (i.e., models built on models, with partial data from the present). I recommend lowering the tone in this section, being more cautious, and recognizing that this variable is not a direct measure of history but rather a hypothesis about when species might have arrived in certain regions, strongly dependent on modeling assumptions. It seems premature to present it as if it were an empirical historical variable.

**Response:** We agree that the assemblage age metric used as proxy for evolutionary time is model-based and relies on assumptions from phylogenies and ancestral reconstructions. We revised the text to make clear that this variable is not a direct historical measurement, but a model-derived hypothesis about the relative timing of lineage arrivals. We also lowered the tone and emphasized its comparative nature and associated uncertainties (lines 328-336).

**Comment 3.12:** I would perhaps change this sentence: "By contrast, evolutionary time and temperature influence richness more weakly and in a context-dependent manner." For: "In contrast, evolutionary time and temperature show weaker, context-dependent effects on richness."

**Response:** This is a great suggestion. We have incorporated it into the text (lines 574-575).

**Comment 3.13:** Add a "." at the end of the Conclusions paragraph (after the "and provide testable predictions to resolve long-standing debates in macroecology and macroevolution").

**Response:** Thank you. We have corrected this mistake.

**Comment 3.14:** The study offers a useful integrative snapshot, but its inferences are inherently correlational: the "equilibrium" narrative is inferred from patterns of standardized path coefficients, not from explicit demographic/speciation-extinction dynamics. Uncertain "assemblage age" hinges on DEC ancestral reconstructions and region definitions, while tip-based speciation metrics (DR/BAMM/ClaDS) emphasize recent rates and omit extinction—so diversification inference is incomplete.

**Response:** We agree that our inferences are correlational and depend on model-based variables, and we have clarified these limitations throughout the manuscript (see lines 328-336 & 698-710). Our objective was not to reconstruct full diversification dynamics, which is impossible, but to evaluate how speciation, evolutionary time, and environmental factors jointly relate to richness across clades. For this reason, we focus on speciation rates rather than diversification rates, mainly because extinction cannot be reliably inferred from molecular phylogenies, and incorporating highly uncertain extinction estimates would reduce rather than improve interpretability. The speciation metrics used here (DR, BAMM, ClaDS) are among the most robust available for present-day rate estimation, and we assessed the consistency of results across all three (lines 551-557). While assemblage age also relies on ancestral reconstructions, we emphasize that it is used as a comparative, model-derived hypothesis rather than an empirical historical variable (lines 328-336). Together, these choices allow us to provide a transparent, integrative assessment of how different processes relate to present-day richness while acknowledging the associated assumptions and uncertainties.

**Comment 3.15:** Analyses on 1° grids might have inflated significance from spatial autocorrelation (also, please, specify in methods not in sup material that the maps have an equal area projection). Multicollinearity, measurement error in environmental layers, and residual correlations across equations can bias coefficients; reporting fit indices and comparing constrained SEMs that encode the four scenarios would strengthen causal claims.

**Response:** We thank the reviewer for raising concerns regarding spatial autocorrelation, projection, and model diagnostics. We now explicitly state in the main text, Methods section (lines 196–199) that we used equal-area projection, rather than only in the Supplementary Materials.

Regarding spatial autocorrelation, we agree that analyses on 1° grid cells inevitably exhibit spatial structure. Path models cannot directly incorporate spatial dependence, but we quantified its magnitude using Moran’s I on model residuals and found substantial autocorrelation (Figures S8). To evaluate whether this influenced our conclusions, we re-run all path models including latitude and longitude as additional predictors. This approach does not eliminate autocorrelation but provides a pragmatic and commonly used sensitivity test. The key results, including the strong and consistent effect of productivity on species richness, remained qualitatively unchanged, although effect sizes varied slightly across clades (Figures S49). We now more explicitly acknowledge these limitations in the main text (lines 557-560).

We now also added model fit indices from each path model (i.e., global SEM goodness-of-fit metrics such as CFI, RMSEA, and SRMR) to provide a more comprehensive evaluation of model performance (Figures S24-S27; lines 408-415). Importantly, CFI and SRMR values typically met standard thresholds, whereas RMSEA was often elevated, a known feature of complex models with high sample sizes. Because our path models adhere to theoretical expectations and all paths are grounded in established macroecological predictions, we interpret them in terms of the relative support for hypothesized relationships rather than relying solely on global fit statistics.

Taken together, we have clarified methods, added fit statistics, conducted spatial sensitivity analyses, and strengthened the discussion of assumptions and limitations. These additions improve transparency while showing that our main conclusions are robust to the issues raised.

**Comment 3.16:** Results from all clades are presented as averages, not including

**Response:** The comment is incomplete, but we assume it refers to Figure 3. This figure presents the average standardized effects across clades for each tetrapod group. Individual clade results are provided in Supplementary Figures S15–S18. We have now added the confidence intervals in Figure 3.

**Comment 3.17:** Also, <50% of the variance is explained under their assumptions for all clades (36-46%), so, the title might be even the opposite. 50% of variance is not explained by productivity.

**Response:** We are not sure we understand this comment. The reported 36-46% of explained variance refers to the average proportion of variance explained within individual clades for each tetrapod class. Given the complexity of macroecological processes, and multiple sources of variability, we consider this substantial. We do not claim that productivity alone explains all variance. Instead, it consistently exerts the strongest effect compared with other predictors, while we indicated that historical and clade-specific factors also contribute, particularly in younger clades. This pattern supports the conceptual scenarios without implying complete determinism by productivity.

**Comment 3.18:** The fact that productivity is linked to richness is not a novel result.

**Response:** We agree that the positive effect of productivity on species richness is well established. Our study does not claim this as novel. Rather, the contribution of our work lies in comparing productivity’s effect with other predictors, showing that it operates mostly directly (not via speciation or history), while temperature, speciation rate, and historical effects are weaker or context-dependent across clades.

**Comment 3.19:** Finally, clade selection via Laplacian spectra (including some paraphyly) may influence results and should be tested for sensitivity to alternative clade definitions.

**Response:** We thank the reviewer for raising this important point. Because clade delimitation can influence comparative inference, we explicitly tested the sensitivity of our results to alternative clade definitions. First, we repeated all analyses after restricting the dataset to monophyletic clades only, excluding the small fraction of paraphyletic modalities identified by the Laplacian spectrum approach. Second, we repeated the entire analytical pipeline using phylogenies based on only molecular data, thereby excluding species with imputed phylogenetic positions. Importantly, removing both paraphyletic clades and imputed species did not alter our main conclusions (see lines 534-542).

Third, we considered alternative clade-definition strategies. Approaches based on taxonomic ranks or pre-selected traits introduce substantial arbitrariness, generate clades of similar ages or sizes, making them poorly suited for testing hypotheses in which time and diversification are explicit predictors. Random node sampling would further introduce strong non-independence among clades, as species would be repeatedly represented across nested clades, violating assumptions of replication and inflating confidence in shared patterns.

In contrast, the Laplacian spectrum approach provides a data-driven and objective method for delimiting non-overlapping, independent clades based on shared diversification structure rather than taxonomic convention. This design allows meaningful evolutionary replication across lineages while minimizing dependence among clades. Importantly, the robustness of our results to both clade monophyly constraints and the exclusion of imputed species confirms that the inferred richness-generating processes reflect general properties of diversification structure rather than sensitivity to particular phylogenetic assumptions. Together, these sensitivity analyses indicate that our conclusions are robust to variation in clade delimitation and phylogenetic uncertainty, and that the Laplacian-based framework provides an appropriate basis for comparative inference at this scale.

# **Reviewer #4**

[identifies himself as Richard Field]

I think this is a wonderful paper! It addresses a classic question in ecology (what causes the latitudinal biodiversity gradients and related patterns? - often considered the 'holy grail of ecology') and provides both a framework for making progress on the theory and empirical results that address key hypotheses. Papers whose conceptual contribution is a mix of methodological and empirical are often difficult for journal editors because usually either, often both, of these advances is rather weak. This is a rare example of a paper in which each of these elements is, in my opinion, a sufficient advance *on its own* to merit publication in a very good journal like PLoS Biology. Putting the two together makes this one of the best and most important papers I have seen in a long time.

In addition, I congratulate the authors on having the manuscript in such good shape on first submission to the journal. I suppose that this is either a remarkable achievement by careful authors or the paper has previously been reviewed at one of the very top journals and improved as a result; it may be both. I would not be surprised if it had undergone revision for one of the very top journals because the conceptual advance provided is so significant. I can also envisage that rejection from such a journal might result because of some matter of reviewer taste (e.g. on very specific aspects of methods or not liking the implications), given how many areas of science this draws from and the implications that many peoples' preferred theories are shown rather convincingly herein to be modifiers rather than the main story. Whatever the pathway to submission to PLoS Biology, the result is that I have unusually little to criticise! (I normally provide many pages of pretty fundamental criticisms.)

I do have a few, relatively minor comments that the authors can consider. I did not find any line numbers in the manuscript, so below I include quotations, where appropriate, to aid location in the manuscript.

**Response:** We sincerely thank the reviewer for the exceptionally positive and encouraging comments. We are very grateful for the recognition of both the conceptual and empirical contributions of our work. We have carefully considered the reviewer’s comments and provide responses below.

**Comment 4.1:** My most general comment is to wonder how fully the authors have thought through possible biases and the potential for circularity. Their review of limitations in the Discussion clearly demonstrates quite a lot of such thinking, and I find the arguments there convincing. From spending only a limited time (less than I would like) thinking about these while reading the manuscript, I am not convinced that there are any major bias or circularity problems. But I encourage the authors (who know their data and analyses much more thoroughly than I do) to consider whether any of the following aspects of their methods and data might lead to any cause for concern about bias or circularity - and either add some sentences to the Discussion accordingly, or if necessary do a few more sensitivity analyses. (I note that there are already lots of sensitivity analyses!)

**Response:** We thank the reviewer for raising this important point. We carefully thought about the potential for circularity arising from our methodological choices, as suggested by the reviewer. After careful evaluation, we identified two potential sources of circularity:

1. Clade delineation via the Laplacian spectrum: Since this method identifies groups of species sharing similar branching patterns, there is a theoretical possibility that clades could cluster lineages with similar tip-based speciation rates or evolutionary times, potentially introducing minor statistical dependencies and reducing variation in some characteristics within clades.
2. Assemblage age from ancestral-range reconstructions: These reconstructions use species range maps to estimate the timing of lineage arrivals in each grid cell. While the same range maps are also used to calculate species richness per cell, the two measures are derived independently: assemblage age is a model-based estimate of arrival times, whereas richness is simply a count of species overlapping each cell. Assemblage age therefore does not deterministically influence richness values. There can still be a link with species richness caused by niche conservatism and a limited spread of species from their region of the origin, but this is the link we test, being one of the explanations of diversity patterns.

To assess the impact of these potential circularities, we conducted extensive sensitivity analyses, including varying the number of clades (40–60 modalities), excluding paraphyletic clades, comparing only trees based on species with molecular data, and using multiple speciation metrics (DR, BAMM, ClaDS). Across all these analyses (see lines 532-560), results remained highly consistent, indicating that any circularity is unlikely to meaningfully influence our conclusions.

We acknowledge these potential circularities in the Discussion (lines 679-758), where we explicitly consider them and explain why they are unlikely to bias our interpretations. Taken together, these considerations suggest that while minor circularities are theoretically possible, they do not compromise the robustness or interpretability of our results.

**Comment 4.2:** The range maps used. I think that they reflect a mix of observed occurrence data and then some form of interpolation - whether formal statistical interpolation or implicit interpolation by expert judgment, or both. Either interpolation method would typically use environmental variables either formally (e.g. by using elevation, precipitation, temperature, etc in an interpolation model) or informally (e.g. by an expert associating a species they know with shaded, wet habitats in mid elevations and mapping the range accordingly). Does the use of the range maps introduce any possible bias or circularity into the results? I'm not sure, but it might.

**Response:** This is a very good point. We do not consider it to create a pronounced circularity, but it is true that geographic ranges of some taxa were interpolated based on knowledge of their habitat associations (mostly reptiles, possibly some amphibians, and much less for birds and mammals). Importantly, although these range maps may incorporate habitat or climatic information, they were generally not constructed with reference to productivity, and certainly not with respect to species richness patterns. Consequently, any potential circularity is expected to be minor and could, at most, slightly elevate the apparent effects of temperature and precipitation on diversity patterns. Notably, this would work against our main finding that productivity is the dominant driver of richness, making our results conservative in this regard. We have now explicitly discussed this potential circularity in the Discussion section under methodological caveats (lines 739-749), together with the other sources of circularity highlighted in response to previous reviewer comments.

**Comment 4.3:** "Historical influences were captured as the timing and likely regional arrival of lineages into assemblages (reconstructed via historical biogeographic models), while evolutionary effects were measured through tip-based speciation rates". I do not know the methods used here intimately enough to know whether any circularity or bias, with respect to the work done here, could creep in through them. I think probably not, but encourage the authors to think this through and respond.

**Response:** This is something we thought about a lot. In an earlier version of the manuscript, we inferred historical effects directly from branch lengths, but we recognized that this approach inherently (negatively) links lineage age to speciation rate, potentially introducing circularity. To avoid this, we adopted ancestral-range reconstructions, which estimate the timing of species arrival in regional assemblages independently of tip-based speciation rates. Although this method is more complex and computationally demanding, it provides a more reliable and conceptually independent measure of historical influence (see lines 543-550). We are confident that the approach used in the current manuscript avoids the circularity issues inherent to branch-length-based metrics and appropriately separates historical from evolutionary effects.

**Comment 4.4:** I think it would be helpful to mention ecological opportunity somewhere in the Discussion and/or Introduction. The authors mention ecological saturation, which is kind-of the opposite, reducing speciation rates (or at least diversification rates). The other side of the coin is ecological opportunity promoting speciation rates, and this fits well with the authors' reasoning about the relationship with species richness, and the context dependency of the role of speciation rates. But, other than indirectly via a single citation of Schluter 2015, ecological opportunity is not actually mentioned in the manuscript.

**Response:** We thank the reviewer for this insightful suggestion. We agree that explicitly incorporating the concept of ecological opportunity strengthens the interpretation of our results, particularly with respect to the weak or negative relationship between speciation rates and species richness (see lines 583-621).

**Comment 4.5:** On the subject of speciation rates, the authors say in the Intro: "Furthermore, recent studies challenge the assumption that speciation rates consistently predict richness, showing that they can be decoupled or even negatively correlated with species richness (Morlon 2020; Rabosky et al. 2018; Sun et al. 2020)." I do not think 'assumption' is the right word here - it is more about proposing this as an explanation than assuming it to be true in order to formulate some other explanation. I suggest the word 'notion' instead.

**Response:** Thank you for this helpful comment! We have updated the text accordingly (lines 64-66).

**Comment 4.6:** Table 1: It seems strange to me that the legends explaining the line colours are in the form of arrows coming out of Richness! Please modify.

**Response:** That is right, thanks for the comment, we have changed it.

**Comment 4.7:** Section 3.1: I like this opener to the Results section. It is a nice, clear statement uncluttered by detail. Even so I would refer to Fig.3 at the start of this paragraph

**Response:** Thank you for the positive feedback. We have added a reference to Figure 3 at the start of the paragraph as suggested.

**Comment 4.8:** Section 3.2: in contrast, this section (and parts of 3.3) are highly cluttered with numbers, hindering readability. Figure 3 is much more effective at getting this information across to the reader (or it should be - see next point). I suggest removing the detailed numbers and instead focus on clearly and effectively narrating the key points in the text, letting Figure 3 take care of the fine details (Lines 466-467).

**Response:** Thank you for this suggestion. Following your advice, we have revised this section to reduce the clutter of detailed numerical values and focus on a clear narrative of the main patterns (lines 476-513).

**Comment 4.9:** Figure 3 should show the confidence intervals (which are currently stated laboriously in the text). It is a basic principle of graphing that mean values should have appropriate error bars. (Note here: the quite innovative approach that the authors used to generate the replication behind these mean values is to me a strength of the paper, giving the ability to apply meaningful confidence intervals - so it should come across in the graphical presentation of the results!)

**Response:** Thank you for the suggestion! We have included the confidence intervals into Figure 3.

**Comment 4.10:** Section 3.4 and the associated parts of the Discussion (e.g. start of 4.2). Yes there is a negative relationship between clade age and effect size, and the authors discuss it well. But I also see in Figure 4A a triangular (or constraining) relationship. There are no large effect sizes for old clades, while there is the full range of effect sizes for young clades. I think recognising this feature of the relationship and discussing it would enhance the manuscript. It is fully in line with the authors' interpretation of the results and actually I think would enhance that interpretation.

**Response:** This is a great point, thanks for it! We have incorporated the discussion of the triangular pattern between clade age and evolutionary time effects in the Discussion (lines 627–630), highlighting that older clades show consistently small effects while younger clades display a wide range of effects.

**Comment 4.11:** Figure 4 vs the text. Figure 4B has 'warming' and 'cooling' on the horizontal axis and the caption says "clades originating in warming vs. cooling periods". In contrast, throughout the text the wording used is 'warmer'/'cooler' and 'warm'/'cool'. Warm is not the same as warming, and cool is not the same as cooling - they are partially offset from each other in time. The authors should establish which it is and then align all the labels accordingly.

**Response:** Thank you for pointing this out. Following the previous recommendation of Reviewer 1 (Comment 1.6), climate origin was removed from our analyses, and Figure 4B was consequently omitted. As a result, any references to “warm/cool” or “warming/cooling” periods in the text have been removed to avoid confusion. Since the variable is no longer included, the issue of inconsistent terminology no longer applies.

**Comment 4.12:** The first two sentences of the Discussion are Introduction material and repeat what has come before. I suggest deleting them and starting the discussion with "By applying a conceptual framework…"

**Response:** We have removed the first two sentences of the Discussion. Thank you!

**Comment 4.13:** While I agree with most, if not all of the interpretation and explanation in the paper, I am conscious that others may not. And it is important to stick to facts rather than interpretations when discussing things as facts. So the words 'strong influence of productivity' in the following sentence at the end of 4.1 concerns me: "Overall, this perspective unifies three key observations: (i) the absence of a positive correlation between present-day speciation rates and species richness, (ii) the context-dependent effect of evolutionary time, and (iii) the consistently strong influence of productivity on richness patterns." This sentence is defining key observations, but I do not agree that "the consistently strong influence of productivity on richness patterns" is an observation. It is an interpretation. The observation is the consistently strong positive relationship between productivity and richness patterns. So I suggest changing "consistently strong influence of productivity on richness patterns" to "consistently strong positive relationship between productivity and richness patterns".

**Response:** We agree that the phrase reflects an interpretation rather than a direct observation. To address this, we have revised the sentence to refer explicitly to the observed pattern (lines 616-621).

**Comment 4.14:** On a similar note, the last paragraph of the Conclusion starts "Our analyses reveal that species richness across most tetrapod clades is primarily governed by equilibrium dynamics driven by productivity." Here I am concerned by the word 'reveal', for similar reasons as articulated above. How about changing the start of the sentence to "We infer from our analyses that species richness…"?

**Response:** We thank the reviewer for this suggestion (lines 772-774).

**Comment 4.15:** End of first paragraph of 4.2: "Figure S29-SX" needs fixing.

**Response:** Thank you! We have fixed that.

**Comment 4.16:** Start of second paragraph of 4.2: here I think it is worth mentioning and briefly discussing the exception, which is the moderately strong direct effect of precipitation on amphibian richness - in addition to the moderately strong indirect effect that all the groups have, which means that the total effect of precipitation is greater for amphibians than for the other groups. This makes ecological sense, given the particular requirements of most amphibians for water. Drawing some attention to this point not only fits with the existing discussion, but I think also would enhance the discussion in the paper.

**Response:** This is a great observation. We have now incorporated a discussion of the amphibian exception in the section (lines 644-653). This addition clarifies the context-dependent nature of climatic effects across tetrapod clades.

**Comment 4.17:** Middle of last paragraph of Conclusion: "temperature exerts mainly direct effects, likely reflecting energetic constraints across trophic levels rather than indirect influences through productivity or diversification." When I read this it immediately seemed to me that it does not match how this is discussed in the Discussion, and checking back agrees with that. The last paragraph of 4.2 is the relevant part of the Discussion, and includes for example "A likely explanation is that temperature serves as a better proxy for resource availability than productivity (NPP) in many tetrapods". I suggest rewording the sentence in the Conclusion so that it matches what is in the Discussion.

**Response:** Thank you for this helpful comment. We have now revised the relevant part of the Conclusion to align precisely with the argument developed in the Discussion.

**Comment 4.18:** Finally, I find the final sentence of the conclusion to be rather weak - vague and a bit of arm-waving. In contrast, the previous sentence is strong. I suggest deleting the final sentence, and finishing on the strength.

**Response:** Thank you for this suggestion. We have removed it and worked on the final sentence to make it stronger (lines 781-784).

**References mentioned in the response letter**

Freckleton, R. P., P. H. Harvey, and M. Pagel. 2002. Phylogenetic Analysis and Comparative Data: A Test and Review of Evidence. Am. Nat. 160:712–726. The University of Chicago Press.

Harvey, M. G., G. A. Bravo, S. Claramunt, A. M. Cuervo, G. E. Derryberry, J. Battilana, G. F. Seeholzer, J. S. McKay, B. C. O’Meara, B. C. Faircloth, S. V. Edwards, J. Pérez-Emán, R. G. Moyle, F. H. Sheldon, A. Aleixo, B. T. Smith, R. T. Chesser, L. F. Silveira, J. Cracraft, R. T. Brumfield, and E. P. Derryberry. 2020. The evolution of a tropical biodiversity hotspot. Science 370:1343–1348. American Association for the Advancement of Science.

Hawkins, B. A., E. E. Porter, and J. A. Felizola Diniz-Filho. 2003. Productivity and History as Predictors of the Latitudinal Diversity Gradient of Terrestrial Birds. Ecology 84:1608–1623.

Hurlbert, A. H., and W. Jetz. 2007. Species richness, hotspots, and the scale dependence of range maps in ecology and conservation. Proc. Natl. Acad. Sci. 104:13384–13389.

Jablonski, D. 2007. Scale and Hierarchy in Macroevolution. Palaeontology 50:87–109.

Jetz, W., G. H. Thomas, J. B. Joy, K. Hartmann, and A. O. Mooers. 2012. The global diversity of birds in space and time. Nature 491:444–448. Nature Publishing Group.

Kline, R. B. 2016. Principles and Practice of Structural Equation Modeling. 4th ed. The Guilford Press, New York, NY.

MacCallum, R. C., M. W. Browne, and H. M. Sugawara. 1996. Power analysis and determination of sample size for covariance structure modeling. Psychol. Methods 1:130–149. American Psychological Association, US.

Martins, E. P., and T. F. Hansen. 1997. Phylogenies and the Comparative Method: A General Approach to Incorporating Phylogenetic Information into the Analysis of Interspecific Data. Am. Nat. 149:646–667. [The University of Chicago Press, The American Society of Naturalists].

Rabosky, D. L. 2009. Ecological limits and diversification rate: alternative paradigms to explain the variation in species richness among clades and regions. Ecol. Lett. 12:735–743.

Ricklefs, R. 2007. History and Diversity: Explorations at the Intersection of Ecology and Evolution. Am. Nat., doi: 10.1086/519402. The University of Chicago Press.

Ricklefs, R. E. 2004. A comprehensive framework for global patterns in biodiversity. Ecol. Lett. 7:1–15.

Saupe, E. E., C. E. Myers, A. T. Peterson, J. Soberón, J. Singarayer, P. Valdes, and H. Qiao. 2019. Spatio-temporal climate change contributes to latitudinal diversity gradients. Nat. Ecol. Evol. 1–11.

Title, P. O., and D. L. Rabosky. 2019. Tip rates, phylogenies and diversification: What are we estimating, and how good are the estimates? Methods Ecol. Evol. 10:821–834.
