# Supplementary material for: Diversity patterns in terrestrial tetrapod clades are governed by equilibrium dynamics
Source: PLoS Biol. 2026 Apr 13;24(4):e3003730. doi: 10.1371/journal.pbio.3003730 (PMC13075687; doi:10.1371/journal.pbio.3003730)
Supplement: Attachment — Submitted filename: Response_to_Reviewers_auresp_3.doc [file pbio.3003730.s005.doc]

**Response to Editor and Reviewers**

We thank the editor and reviewers for their careful evaluation of the revised manuscript and for their positive assessment of our work. We greatly appreciate the time and effort invested in reviewing the manuscript throughout the revision process, as well as the constructive feedback provided at each stage. Their comments have helped us further improve the clarity, transparency, and overall quality of the manuscript.

In this final revision, we have carefully addressed all remaining editorial requests and minor points raised by the editor and reviewers. The manuscript and associated materials have been updated accordingly. Below we provide a detailed, point-by-point response to each comment and indicate the corresponding changes made in the manuscript.

**Editor comments**

**Comment 1:**

Please address the remaining very minor points from the reviewers; note that this includes a minor typo in the Title.

**Response:**
We thank the editor and reviewer for pointing this out. In the original title, the phrase *“equilibrium dynamics”* was used in the sense commonly adopted in the literature, where it refers to a conceptual framework and can therefore be treated as a singular entity. However, we recognize that the term *dynamics* may also be interpreted as plural, which could introduce ambiguity regarding subject–verb agreement. To avoid any potential confusion, we slightly revised the structure of the title. The original title, *“Equilibrium dynamics governs diversity patterns in terrestrial tetrapod clades,”* has been changed to *“Diversity patterns in terrestrial tetrapod clades are governed by equilibrium dynamics.”* This modification preserves the intended meaning of the title while avoiding possible ambiguity in grammatical interpretation.

**Comment 2:**
Please address my Data Policy requests below; specifically, we need you to supply the numerical values underlying Figs 3, 4, 5, 6A, S1–S49, either as a supplementary data file or as a permanent DOI’d deposition. I note that you already have an associated Zenodo deposition (<https://doi.org/10.5281/zenodo.17020157>). Please could you confirm whether the data and code in this deposition are sufficient to recreate the Figures?

**Response:**
All numerical data underlying Figs 3, 4, 5, 6A, and S1–S49, as well as the R scripts used to generate the figures, are publicly available in the Zenodo repository under the Attribution 4.0 International license (DOI:<https://doi.org/10.5281/zenodo.14008084>). The repository contains CSV and RDS files providing the numerical values used in the analyses and figure construction, together with the custom R scripts used to perform the analyses and generate the figures presented in the manuscript. These materials include the individual values underlying the plotted summaries, thereby allowing the main analyses and figures to be reproduced directly from the deposited files. The repository also includes documentation describing the structure of the deposited data and scripts to facilitate reproducibility and reuse.

**Comment 3:**
Please cite the location of the data clearly in all relevant main and supplementary Figure legends, e.g. “The data and code needed to generate this Figure can be found in<https://zenodo.org/records/17020157”>.

**Response:**
We have added the following statement to all relevant main and supplementary figure legends: “The data underlying this figure can be found in [https://doi.org/10.5281/zenodo.14008084”](https://doi.org/10.5281/zenodo.14008084.”)

**Comment 4:**
Please make any custom code available, either as a supplementary file or as part of your data deposition.

**Response:**
All custom R code used in the analyses is included in the Zenodo repository associated with this study (<https://doi.org/10.5281/zenodo.14008084>).

**Comment 5:**
Please include the URLs of your funders in the Financial Disclosure statement.

**Response:**
We have updated the Financial Disclosure statement to include the URLs of the relevant funding agencies.

**Comment 6:**
We note that you have a large number of references in the supplementary material; these citations will not receive recognition unless they are also in the main references.

**Response:**

Thank you for bringing this to our attention. The supplementary methods previously provided an expanded description of the methodological procedures already summarized in the main text, and therefore most of the references cited in the supplementary material were redundant with those already included in the main reference list. To avoid duplication and ensure that all citations are properly indexed, we removed the reference list from the supplementary material and retained all relevant citations in the main manuscript. The only references that were not previously cited in the main text were those associated with R packages used in the analyses (e.g., *phangorn*, *phytools*, and *terra*). These references have now been incorporated into the relevant sections of the Methods in the main manuscript, ensuring that all cited works appear in the primary reference list.

**Reviewer comments**

**Reviewer #1**

**Comment:**
I appreciate the hard work the authors have put into these revisions. I was one of the reviewers who came on board at R1, and I know it can be annoying to get a new set of comments at that stage. I think this paper is very well written and exceptionally thorough. The authors ability to conduct a large number of robustness checks (removing paraphyletic clades, using molecular-only species in the phylogeny, choice of evolutionary-time metric, speciation rate metric, and effect of spatial autocorrelation) requested by myself and others gives me a lot of additional confidence in the results. This is going above-and-beyond and the authors deserve a lot of credit for this. For example, repeating the analysis on the molecular-only data gives me a lot more confidence in the Laplacian spectrum approach and softens my anxiety that the megaphylogenies used in this study might not be the best datasets for the question.

I'm also glad the authors liked the suggestion of the model selection for the SEM analysis. I feel like this does nicely streamline that section and make it easier to interpret as a reader.

I don't think I agree with the authors that non-identifiability propagates to net-diversification rates in the same way as for speciation and extinction rates, but I do think the additional text the authors have contributed is sufficient here, and the authors have done more than enough to satisfy any concerns I had.

**Response:**
We thank the reviewer for their positive evaluation of the revised manuscript and for their constructive suggestions throughout the review process. We are pleased that the additional robustness analyses and clarifications have strengthened confidence in the results. Thank you!

**Reviewer #4 (Richard Field)**

**Comment:**
I have read through all the previous reviews and the authors' responses to them, as well as the revised manuscript. As well as doing everything I asked of them, in my opinion, the authors have replied very thoroughly to all the comments in the reviews, taking all of them seriously, making appropriate revisions to the paper and doing a lot of new sensitivity analyses even though it seemed clear to me that the results would not change meaningfully - as has turned out to be the case.

In my view, this is ready to publish, pending a good copy edit, including correction of a typo in the title: change 'governs' to 'govern'.

**Response:**
We thank the reviewer for their careful reading of the manuscript and for their positive assessment of the revisions. We also appreciate the suggestion regarding the title. In the original formulation, the phrase *“equilibrium dynamics”* was used in the sense commonly adopted in the literature, where it refers to a conceptual framework and can therefore be treated as a singular entity. However, we recognize that the term *dynamics* may also be interpreted as plural, which could introduce ambiguity in subject–verb agreement. To avoid any potential confusion, we slightly revised the structure of the title. The original title, *“Equilibrium dynamics governs diversity patterns in terrestrial tetrapod clades,”* has been changed to *“Diversity patterns in terrestrial tetrapod clades are governed by equilibrium dynamics.”* This modification preserves the intended meaning of the title while avoiding possible ambiguity in grammatical interpretation.
